# Efficient Mimics for Elucidating Zaxinone Biology and Promoting Agricultural Applications

**DOI:** 10.1016/j.molp.2020.08.009

**Published:** 2020-11-02

**Authors:** Jian You Wang, Muhammad Jamil, Pei-Yu Lin, Tsuyoshi Ota, Valentina Fiorilli, Mara Novero, Randa A. Zarban, Boubacar A. Kountche, Ikuo Takahashi, Claudio Martínez, Luisa Lanfranco, Paola Bonfante, Angel R. de Lera, Tadao Asami, Salim Al-Babili

**Affiliations:** 1 King Abdullah University of Science and Technology, Division of Biological and Environmental Science and Engineering, the BioActives Lab, Thuwal 23955-6900, Saudi Arabia; 2 Graduate School of Agricultural and Life Sciences, The University of Tokyo, Tokyo, Japan; 3 Department of Life Sciences and Systems Biology, University of Torino, Torino, Italy; 4 Universidade de Vigo, Facultade de Química and CINBIO, Vigo, Spain

**Keywords:** apocarotenoids, zaxinone, zaxinone mimics, strigolactone, *Striga*, root parasitic plants

## Abstract

Zaxinone is an apocarotenoid regulatory metabolite required for normal rice growth and development. In addition, zaxinone has a large application potential in agriculture, due to its growth-promoting activity and capability to alleviate infestation by the root parasitic plant *Striga* through decreasing strigolactone (SL) production. However, zaxinone is poorly accessible to the scientific community because of its laborious organic synthesis that impedes its further investigation and utilization. In this study, we developed easy-to-synthesize and highly efficient mimics of zaxinone (MiZax). We performed a structure–activity relationship study using a series of apocarotenoids distinguished from zaxinone by different structural features. Using the obtained results, we designed several phenyl-based compounds synthesized with a high-yield through a simple method. Activity tests showed that MiZax3 and MiZax5 exert zaxinone activity in rescuing root growth of a zaxinone-deficient rice mutant, promoting growth, and reducing SL content in roots and root exudates of wild-type plants. Moreover, these compounds were at least as efficient as zaxinone in suppressing transcript level of SL biosynthesis genes and in alleviating *Striga* infestation under greenhouse conditions, and did not negatively impact mycorrhization. Taken together, MiZax are a promising tool for elucidating zaxinone biology and investigating rice development, and suitable candidates for combating *Striga* and increasing crop growth.

## Teaser

Zaxinone is an apocarotenoid growth regulator and plant hormone candidate that promotes rice growth and acts as a negative regulator of strigolactone biosynthesis. Wang et al. performed a structure–activity relationship study and used obtained results to generate a series of easy-to-synthesize mimics of zaxinone (MiZax) that were as efficient as zaxinone in promoting rice growth, repressing strigolactone biosynthesis, and alleviating infestation by the parasitic weed *Striga*. The developed MiZax will significantly support zaxinone research and are promising candidates for agricultural applications.

## Introduction

Chemical signals and hormones are involved in literally all aspects of a plant's life. These small molecules are key regulators of plant development and response to environmental stimuli, and the means of communication between plants and surrounding organisms (Chaiwanon et al., 2016; Guerrieri et al., 2019). Strigolactones (SLs) are an intriguing example for signaling molecules that fulfill both functions. They act as a hormone that determines diverse processes within plant, which include shoot branching, growth of primary, lateral and adventitious roots, and biotic and abiotic stress responses (Al-Babili and Bouwmeester, 2015; Waters et al., 2017; Jia et al., 2018). In addition, SLs are released into the rhizosphere, particularly under phosphate starvation, as signaling molecules that facilitate the recruitment of arbuscular mycorrhizal (AM) fungi for establishing the beneficial AM symbiosis (Bonfante and Genre, 2008; Gutjahr and Parniske, 2013; Lanfranco et al., 2018). However, obligate root parasitic plants of the *Orobanchaceae* family have evolved specific receptors that trigger the germination of their seeds upon perceiving rhizospheric SLs. This mechanism enables synchronizing the germination with the availability of a host in close neighborhood, which ensures the survival of the arising parasite seedling (Xie and Yoneyama, 2010). Root parasitic plants, such as *Striga* spp., are a severe agricultural problem in warm and temperate zones (Parker, 2012). Indeed, *Striga hermonthica* that infests cereals, such as rice, sorghum, pearl millet, and maize, is one of the major threats to global food security, as it causes enormous yield losses in different regions of Africa (Pennisi, 2010; Parker, 2012).

SLs consist of a butenolide ring (D-ring) that is connected by an enol bridge of (*R*)-configuration to a less structurally conserved second moiety (Al-Babili and Bouwmeester, 2015; Jia et al., 2018). SLs derive from carotenoids, essential isoprenoid photosynthetic pigments equipped with conjugated double bonds varying in their stereo-configuration (Moise et al., 2014). The enzyme DWARF27 in rice and orthologs from other plants initiate SL biosynthesis by isomerizing all-*trans*- to 9-*cis*-β-carotene (Bruno and Al-Babili, 2016; Abuauf et al., 2018), which is subjected in the next step to a stereospecific cleavage catalyzed by the carotenoid cleavage dioxygenase 7 (CCD7) that forms the volatile β-ionone and a 9-*cis*-configured apocarotenoid intermediate (Bruno et al., 2014). The latter is then converted by CCD8 via a combination of repeated oxygenation and other less understood reactions into the central SL biosynthesis intermediate carlactone (Alder et al., 2012; Bruno et al., 2017). In the next steps, cytochrome P450s, such as the *Arabidopsis* MAX1 or the rice carlactone oxidase (CO), together with other enzymes transform carlactone into different SLs, giving rise to the structural diversity of these compounds (Abe et al., 2014; Zhang et al., 2014; Brewer et al., 2016; Yoneyama et al., 2018; Wakabayashi et al., 2019).

Besides SLs and abscisic acid, carotenoids are the precursor of several regulatory metabolites, including cyclocitral, zaxinone, and anchorene (Dickinson et al., 2019; Jia et al., 2019; Wang et al., 2019). Recently, we showed that the apocarotenoid, i.e., carotenoid cleavage product zaxinone, is a common plant metabolite that determines rice growth and development (Wang et al., 2019). Zaxinone biosynthesis is catalyzed in rice by the zaxinone synthase (ZAS), a member of a less-characterized plant CCD subfamily (Wang et al., 2019). The rice *zas* mutant shows growth retardation, lower zaxinone levels in roots, and higher SL content in roots and root exudates. These phenotypes could be rescued, to a large extent, by exogenous application of synthetic zaxinone that promoted root growth and reduced SL content and release also in wild-type plants. Expression analysis of treated *zas* and wild-type plants suggested that zaxinone suppressed the transcript level of SL biosynthetic genes under phosphate starvation. Moreover, application of zaxinone to rice plants under greenhouse conditions significantly decreased *Striga* emergence, likely by lowering SL release. These results demonstrate the importance of zaxinone for basic plant science as well its application potential for improving crop growth, regulating shoot branching, and controlling *Striga*. However, further investigation of the biological functions of zaxinone, its interaction with plant hormones, as well as its application potential are hampered by the laborious synthesis (see Supplemental Figure 1) of this compound, which makes it poorly accessible to the scientific community.

Analogs and mimics of hormones are frequently used in basic research as well as in agricultural and horticultural applications (Rigal et al., 2014; Koprna et al., 2016; Screpanti et al., 2016). This is particularly the case if the bioactivity of the authentic metabolite is short living (Rigal et al., 2014; Vaidya et al., 2019) or if its natural sources are restricted and organic synthesis is complicated. SLs are a best example for the latter case. The scarcity of SLs has prompted researchers to use mimics and analogs, mainly GR24, which have been decisive in elucidating SL biology and even in the discovery of the SL hormonal function (Gomez-Roldan et al., 2008; Umehara et al., 2008; Al-Babili and Bouwmeester, 2015). Similarly, agricultural applications of SLs, such as inducing suicidal germination of root parasitic weeds, rely on different analogs (Samejima et al., 2016; Vurro et al., 2016; Jamil et al., 2018, 2019, 2020; Kountche et al., 2019).

In this work, we developed the first reported series of zaxinone mimics. For this purpose, we first performed a structure–activity relationship study that allowed us to identify structural features required for zaxinone activity. Next, we designed easy-to-synthesize mimics of zaxinone (MiZax) and characterized their biological activities in regulating SL biosynthesis and rice growth, and alleviating *Striga* infestation. Results obtained demonstrate the efficiency of these MiZax and their utility for zaxinone-related studies and applications.

## Results and Discussion

### Chain Length, Stereo-Configuration, and the Ketone Functional Group Are Essential for Zaxinone Activity

**Figure 1:**
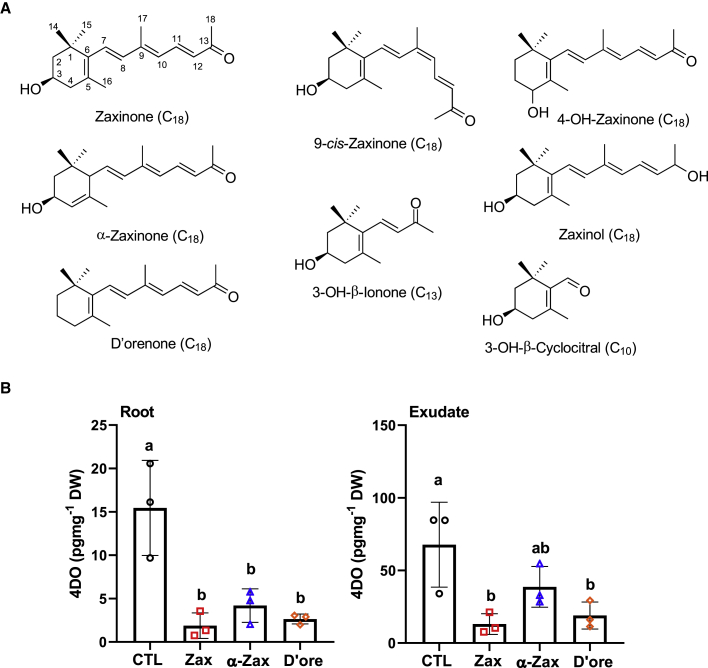
Structure and Effect of Apocarotenoids on SL Content in Root Tissues and Exudates. **(A)** Structures of apocarotenoids used in the structure–activity relationship study. **(B)** SL quantification (4-deoxyorobanchol [4-DO]) in wild-type root tissues and exudates in response to zaxinone (Zax), α-zaxinone (α-Zax), and D'orenone (D'ore) treatment (5 μM) under Pi starvation. Bars represent mean ± SD; *n* = 3 biological replicates; statistical analysis was performed using one-way analysis of variance (ANOVA) and Tukey's *post hoc* test. Different letters denote significant differences (*P* < 0.05). CTL, control; Zax, zaxinone; α-Zax, α-zaxinone; and D'ore, D'orenone.

Identifying structural elements required for activity is a crucial step in rational design of hormone analogs/mimics. Zaxinone is a C_18_-ketone consisting of a linear, all-*trans*-configured isoprenoid polyene linked to a β-ionone ring carrying a hydroxy group at the C3 position (Figure 1A). The functional ketone group of zaxinone is separated from the β-ionone ring by a chain with a length of six C atoms. To perform the structure–activity relationship study, we synthesized a series of apocarotenoids that differ from zaxinone in the polyene length, its stereo-configuration, the type of the ionone ring, or the position of the hydroxy group. We also synthesized zaxinol, in which we replaced the ketone of zaxinone by a hydroxy group, and D'orenone that lacks the hydroxy group at C3 position (Figure 1A). Next, we applied these compounds to hydroponically grown wild-type seedlings exposed to 1 week Pi starvation, and quantified 4-deoxyorobanchol, a major rice SL, in roots and root exudates, using liquid chromatography-tandem mass spectrometry (LC-MS/MS). The shorter apocarotenoids 3-OH-β-cyclocitral and 3-OH-β-ionone, the *cis*-configured 9-*cis*-zaxinone, zaxinol, and 4-OH-β-apo-13-carotenone did not significantly impact 4-deoxyorobanchol content in roots or root exudates (Supplemental Figure 2), suggesting that chain length, stereo-configuration, the presence of the ketone group, and the position of the hydroxy group are important for exerting zaxinone activity. In contrast, the application of α-zaxinone and, particularly, D'orenone decreased SL content to levels comparable with those observed upon treatment with zaxinone, with the latter being the most efficient compound followed by D'orenone (Figure 1B). These data suggest that all-*trans*-C_13_-apocarotenones (C_18_-ketones) can generally repress SL production and that the presence of the hydroxy group and the position of the double bond in the ionone ring have less impact on this activity.

### Synthesis and Screening of Zaxinone Mimics

**Figure 2:**
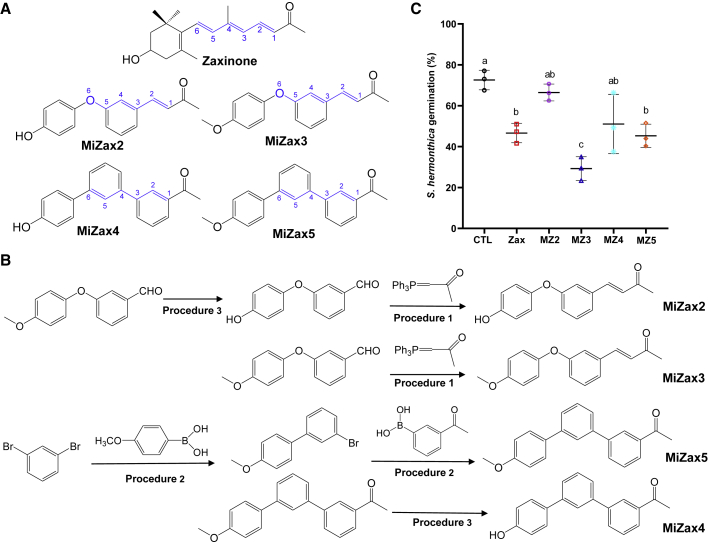
Synthesis and Screening of MiZax. **(A)** Chemical structure of zaxinone and MiZax2-5. **(B)** Synthesis scheme of MiZax, (*E*)-4-(3-(4-hydroxyphenoxy)phenyl)but-3-en-2-one (MiZax2), (*E*)-4-(3-(4- methoxyphenoxy)phenyl)but-3-en-2-one (MiZax3), 1-(4″-hydroxy-[1,1':3′,1″-terphenyl]-3-yl)ethan-1-one (MiZax4), and 1-(4″-methoxy-[1,1':3′,1″-terphenyl]-3-yl)ethan-1-one (MiZax5). Numbers in blue indicate the distance between phenyl ring and the ketone group. The detailed synthetic methods are provided in Supplemental Document S3. **(C)***Striga* seed germination activity of rice root exudates isolated from plants treated with zaxinone or MiZax2-5. Bars represent means ± SD; *n* = 3 biological replicates. Statistical analysis was performed using ANOVA and Tukey's *post hoc* test. Different letters denote significant differences (*P* < 0.05). CTL, control; Zax, zaxinone; MZ2, MiZax2; MZ3, MiZax3; MZ4, MiZax4; MZ5, MiZax5.

Zaxinone is a natural apocarotenoid characterized by a conjugated isoprenoid chain. The synthesis of zaxinone requires five steps and has a moderate yield (47% or less; Supplemental Figure 1). We aimed at the development of efficient MiZax, which can be synthesized in significantly fewer steps and at higher yield. To achieve this goal, we relied on the results of the structure–activity relationship study and decided to substitute the conjugated isoprenoid chain of zaxinone by aromatic structures. This was inspired by several successful examples, such as the development of the fungicides azoxystrobin and metominostrobin (Bartlett et al., 2002) and the insecticide fenoxycarb from natural isoprenoid bioactive compounds (Thind and Edwards, 1986). We also chose the replacement of the β-ionone ring of zaxinone by a phenyl ring, which is a common approach in designing SL analogs (Boyer et al., 2014; Jia et al., 2016; Takeuchi et al., 2018). To evaluate the biological activity of the designed mimics, we determined their effect on SL content in root exudates, using LC-MS/MS quantification or *Striga* seed germination as a bioassay. In a first attempt, we designed MiZax1 that contains phenyl rings instead of the β-ionone ring and part of the conjugated chain (Supplemental Figure 3A). MiZax1 was synthesized in only two steps. However, application of this compound did not significantly impact the SL level in root exudates of treated rice plants (Supplemental Figure 3B). The distance between the ketone group and the phenyl ring in MiZax1 is five C atoms, i.e., one C atom shorter than in zaxinone. Hence, we hypothesized that the missing activity of this mimic might be a result of the short chain length. Therefore, we designed further four mimics (Figure 2A) in which the phenyl ring and the ketone group are separated by a chain of six atoms, and the zaxinone isoprenoid chain is substituted by two phenyl rings (MiZax4 and -5) or partially replaced by a phenoxy ring (MiZax2 and -3). The hydroxy group in MiZax3 and MiZax5 was methylated, to increase their hydrophobicity and account for methylation as a possible zaxinone modification *in planta* (Figure 2A). The four mimics were synthesized in one or two steps (Figure 2B), with yield rates ranging from 11% (MiZax4) to 81% (MiZax3).

To test the hypothesis on the effect of the chain length, we measured the SL content in root exudates of rice plants treated with MiZax1, MiZax2, or MiZax4. In comparison with MiZax1 and mock control and supporting our hypothesis, application of MiZax2 led to a significant decrease in SL level and *Striga* germination rate, while MiZax4 showed a tendency to reduce SLs, particularly orobanchol, release (Supplemental Figure 4). Besides a common chain length, MiZax3 and MiZax5 contain a methoxy group instead of the hydroxy group at C3 in zaxinone and the corresponding position in MiZax2 and MiZax4. Comparison of the effect of MiZax2 and MiZax3, and of MiZax4 and MiZax5, on *Striga* seed-germinating activity demonstrated that this methylation has a significant positive effect on the activity of zaxinone mimics (Figure 2C). Hence, we speculated that zaxinone is converted into methyl-zaxinone *in planta.* To test this possibility, we synthesized methyl-zaxinone and checked its presence *in planta* as well as its biological activity. However, we could not detect methyl-zaxinone in rice plants (data not shown). In addition, the biological efficiency of methyl-zaxinone in inducing *Striga* seed germination was similar to that of zaxinone (Supplemental Figure 5), indicating that the presence of the methoxy group *per se* is not the reason of the increased activity of MiZax3 and MiZax5 and that direct zaxinone methylation might not take place *in planta*. Supporting the latter conclusion, we did not detect a conversion of MiZax2 or MiZax4 into MiZax3 or MiZax5, respectively, in rice plants fed with the former two mimics (Supplemental Figure 6). These data indicate that the higher activity observed with MiZax3 and MiZax5 could be a result of increased hydrophobicity caused by the methyl group, which may improve their uptake and transport. Indeed, we detected MiZax3 and MiZax5 in shoots of rice plants fed with these compounds through roots using LC-MS analysis (Supplemental Figure 7). We also observed a positive effect of the presence of a phenoxy group in MiZax3 instead of the unmodified phenyl ring in MiZax5. This difference might be due to an increased stability caused by a shorter conjugated double bond system and/or the ether bond. To check this assumption, we determined the stability of MiZax3 and MiZax5. For this purpose, we monitored the degradation of these compounds for up to 2 weeks by high-performance liquid chromatography quantification of corresponding aqueous samples kept at room temperature. This study showed that MiZax3 is much more stable than MiZax5 (Supplemental Figure 8), which might explain its higher activity.

### MiZax3 and MiZax5 Are Negative Regulators of Rice SL Biosynthesis and Release

**Figure 3:**
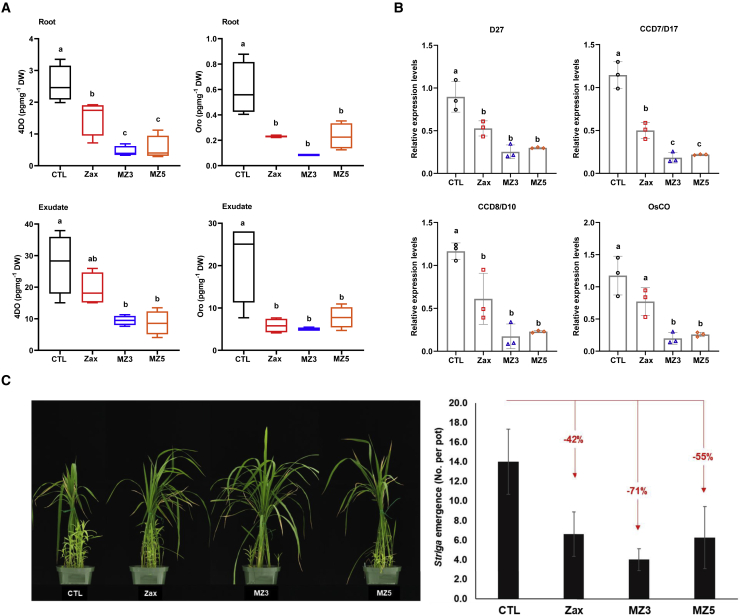
Effect of MiZax3 and MiZax5 on SL Biosynthesis and Release. **(A)** Quantification of the SLs 4-deoxyorobanchol (4-DO) and orobanchol (Oro) in rice roots and root exudates in response to zaxinone, MZ3, and MZ5 application (5 μM) under Pi starvation. Bars represent means ± SD, *n* = 4 biological replicates. **(B)** Relative transcript levels of SL biosynthesis genes (*D27*, *CCD7*, *CCD8*, and *CO*) in response to zaxinone, MZ3, and MZ5 application. Transcript levels in wild-type control samples were normalized to 1. Bars represent means ± SD, *n* = 3 biological replicates. **(C)***Striga* infestation in rice in response to zaxinone, MZ3, and MZ5 treatment (5 μM). Bars represent mean ± SE; *n* = 4 biological replicates. Statistical analysis was performed using ANOVA and Tukey's *post hoc* test. CTL, control; Zax, zaxinone; MZ3, MiZax3; MZ5, MiZax5.

**Figure 4:**
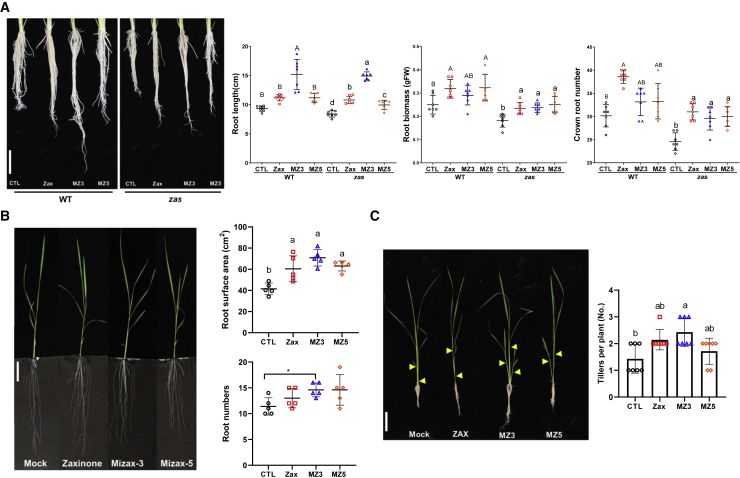
Effect of MiZax3 and MiZax5 on Rice Growth. **(A)** Effect of zaxinone, MZ3, and MZ5 application (2.5 μM) on root growth of wild-type and *zas* mutant seedlings grown under hydroponic conditions. Scale bars correspond to 2 cm. **(B)** Effect of zaxinone, MZ3, and MZ5 application (5 μM) on rice root growth under rhizotron conditions. Scale bars correspond to 8 cm. **(C)** Effect of zaxinone, MZ3, and MZ5 application (2.5 μM) on rice tillering. Tillers are indicated by yellow arrows points. Scale bars correspond to 6 cm. Each data point represents one plant (**A**) *n* = 6; (**B**) *n* = 5; (**C**) *n* = 7. Data represent mean ± SD. Statistical analysis was performed using ANOVA and Tukey's *post hoc* test or *t*-test. Different letters denote significant differences (*P* < 0.05). CTL, control; Zax, zaxinone; MZ3, MiZax3; MZ5, MiZax5.

We evaluated the zaxinone activity of the four mimics using *Striga* seed germination assay performed with root exudates of rice plants treated with 5 μM of each MiZax. Results obtained unraveled a significant negative impact of MiZax3 and MiZax5 treatment on *Striga* seed-germinating activity, which was not observed with MiZax2 and MiZax4 (Figure 2C). Exudates of MiZax3-treated plants showed lowest seed-germinating activity (29%) followed by zaxinone (45%) and MiZax5 (46%). Next, we measured orobanchol and 4-deoxyorobanchol content in root tissues and exudates of hydroponically grown, Pi-starved wild-type seedlings after treatment with 5 μM MiZax3 or MiZax5 for 6 h using LC-MS/MS. Application of both mimics decreased the level of the two SLs in both roots and root exudates (Figure 3A). The effect of MiZax3 or MiZax5 was similar to that of zaxinone and even significantly stronger in the case of 4-deoxyorobanchol. The two mimics, particularly MiZax3, rescued the high SL phenotype of the rice *zas* mutant (Figure 4A; Supplemental Figure 9), similar to zaxinone (Wang et al., 2019). Subsequently, we determined the transcript level of the SL biosynthetic genes *D27*, *CCD7*, *CCD8*, and *CO* in roots from the same experiment. Application of MiZax3 and MiZax5 led to a pronounced decrease in the transcript level of the four enzymes, which was—at least in the case of *CCD7* and *CO* transcripts—significantly lower than that observed with zaxinone (Figure 3B).

The high activity of MiZax3 and MiZax5 in suppressing SL biosynthesis and release indicated their potential in combating *Striga* and other root parasitic weeds, similar to zaxinone. To test this hypothesis, we applied the two mimics at a 5 μM concentration to the *Striga* susceptible cv. IAC-165 rice plants grown in *Striga*-infested soil under greenhouse conditions. Treatment with these compounds led to a clear reduction in the number of emerging *Striga* plants, with the highest reduction observed with MiZax3 (71%), followed by MiZax5 (55%) and zaxinone (42%) (Figure 3C). Considering the important role of SL in the establishment of the AM symbiosis (Fiorilli et al., 2019), we checked the impact of MiZax on AM spore germination and on the colonization process. For this purpose, we treated *Gigaspora margarita* spores with MiZax3 and MiZax5 at a concentration of 5 μM or 50 nM, using the SL analog GR24 (10 nM) as a positive control. After 3 days incubation, GR24 induced, as expected, the germination rate, while no effect was observed for the two mimics (Supplemental Figures 10 and 11), as with zaxinone treatment (Supplemental Figure 12). We also did not detect any alteration in intraradical fungal structures or colonization rate (Supplemental Figure 13A and 13B). In line with this result, the expression levels of the AM marker genes *OsPt11* and *OsLysM* (Fiorilli et al., 2015), and the fungal housekeeping gene (*Fm18S rRNA*) did not show any significant difference between control and 5 μM treated plants. The 50nM treatment even induced a slight upregulation of these AM marker genes (Supplemental Figure 13C). These results indicate that the two mimics would not have a negative side effect on AM fungi and mycorrhization if applied 10 days after inoculation (see Supplemental Methods).

### MiZax Exert Zaxinone Activity in Regulating Rice Growth and Development

Apart from regulating SL biosynthesis and release, zaxinone mimics should be able to rescue growth retardation of the rice *zas* mutant and promote the growth of wild-type plants. To check the capability of MiZax3 and MiZax5 in regulating rice growth, we exposed hydroponically grown *zas* and wild-type (cv. Nipponbare) seedlings to 2.5 μM MiZax3, MiZax5, or zaxinone for 3 weeks. Similar to zaxinone, treatment with the two mimics promoted root growth in wild-type seedlings, by increasing root length and number of crown roots, and rescued root-related *zas* phenotypes, including root biomass (Figure 4A). The two MiZax also triggered root growth of wild-type plants. Moreover, MiZax3 was more active than zaxinone in increasing the root length of wild-type plants in the hydroponic system (Figure 4A). Next, we investigated the effect of the two MiZax in soil at a 5 μM concentration, using the rhizotron system and in comparison with zaxinone. MiZax3 and MiZax5 increased root surface area and the number of crown roots in wild-type plants, similar to zaxinone (Figure 4B). Tillering is a SL-dependent developmental process affected by zaxinone, as shown for the *zas* mutant (Wang et al., 2019). To check if MiZax3 and MiZax5 can also regulate tillering, we exposed rice Nipponbare and IAC-165 (a high SL-producing cultivar) seedlings to these two mimics for 14 days and determined tillers number. We observed a clear promotion of tillers number in both cultivars upon treatment with MiZax3 and a tendency toward more tillers with zaxinone and MiZax5 (Figure 4C and Supplemental Figure 14), which is in line with decreased SL production (Figure 3B). Although MiZax are promising candidates for promoting crop growth and alleviating *Striga* infestation, further studies about their health safety and environmental impact are needed.

In summary, we have developed two high-efficient mimics of zaxinone, which will pave the way for a better understanding of rice growth and development, and the role of zaxinone in this complex process. Moreover, the pronounced activity, simple synthesis (one step, Figure 2B) and relative stability of MiZax3 make it an excellent candidate for different sustainable agricultural applications, including the use of the beneficial AM fungi and the control of *Striga* that severely threatens global food security.

## Methods

Detailed methods are available in Supplemental Document 1.

## Funding

This work was supported by the 10.13039/100000865Bill & Melinda Gates Foundation (grant no. OPP1194472) and a Competitive Research Grant (CRG2017) to S.A.-B. from 10.13039/501100004052King Abdullah University of Science and Technology (KAUST), and by grants from the 10.13039/501100003382Core Research for Evolutional Science and Technology (CREST) Program and the SATREPS Program of the 10.13039/501100002241Japan Science and Technology Agency (JST), and 10.13039/501100001691JSPS Grant-in-Aid for Scientific Research (grant no. 18H03939) to T.A.

## Author Contributions

S.A.-B., and T.A. proposed the concept and designed the experiments. J.Y.W., M.J., P.-Y.L., V.F., M.N., R.A.Z., and B.A.K. performed the experiments. T.A. designed and synthesized MiZax. T.O. and I.T. synthesized MiZax. C.M. and A.R.de.L. synthesized apocarotenoids used for the structure–activity relationship experiments. J.Y.W., V.F., L.L., P.B., T.A., and S.A.-B. analyzed the data. S.A.-B. and J.Y.W. wrote the manuscript. All authors read and approved the manuscript.

## Supplemental Information

Document S1. Supplemental Methods, Supplemental Figures 1–14, Supplemental Table 1 and Supplemental References

Document S2. Synthesis of Enantiopure Zaxinone and Analogs. Supplemental References

Document S3. Protocols for the Synthesis of MiZax

Document S4. Article plus Supplemental Information

## References

[bib1] Abe S., Sado A., Tanaka K., Kisugi T., Asami K., Ota S., Kim H.I., Yoneyama K., Xie X., Ohnishi T. (2014). Carlactone is converted to carlactonoic acid by MAX1 in *Arabidopsis* and its methyl ester can directly interact with AtD14 in vitro. Proc. Natl. Acad. Sci. U S A.

[bib2] Abuauf H., Haider I., Jia K.P., Ablazov A., Mi J., Blilou I., Al-Babili S. (2018). The *Arabidopsis* DWARF27 gene encodes an all-*trans*-/9-*cis*-β-carotene isomerase and is induced by auxin, abscisic acid and phosphate deficiency. Plant Sci..

[bib3] Al-Babili S., Bouwmeester H.J. (2015). Strigolactones, a novel carotenoid-derived plant hormone. Annu. Rev. Plant Biol..

[bib4] Alder A., Jamil M., Marzorati M., Bruno M., Vermathen M., Bigler P., Ghisla S., Bouwmeester H., Beyer P., Al-Babili S. (2012). The path from beta-carotene to carlactone, a strigolactone-like plant hormone. Science.

[bib6] Bartlett D.W., Clough J.M., Godwin J.R., Hall A.A., Hamer M., Parr-Dobrzanski B. (2002). The strobilurin fungicides. Pest Manag. Sci..

[bib7] Bonfante P., Genre A. (2008). Plants and arbuscular mycorrhizal fungi: an evolutionary-developmental perspective. Trends Plant Sci..

[bib8] Boyer F.D., de Saint Germain A., Pouvreau J.B., Clavé G., Pillot J.P., Roux A., Rasmussen A., Depuydt S., Lauressergues D., Frei Dit Frey N. (2014). New strigolactone analogs as plant hormones with low activities in the rhizosphere. Mol. Plant.

[bib9] Brewer P.B., Yoneyama K., Filardo F., Meyers E., Scaffidi A., Frickey T., Akiyama K., Seto Y., Dun E.A., Cremer J.E. (2016). LATERAL BRANCHING OXIDOREDUCTASE acts in the final stages of strigolactone biosynthesis in *Arabidopsis*. Proc. Natl. Acad. Sci. U S A.

[bib10] Bruno M., Vermathen M., Alder A., Wüst F., Schaub P., Van-der-Steen R., Beyer P., Ghisla S., Al-Babili S. (2017). Insights into the formation of carlactone from in-depth analysis of the CCD8-catalyzed reactions. FEBS Lett..

[bib11] Bruno M., Al-Babili S. (2016). On the substrate specificity of the rice strigolactone biosynthesis enzyme DWARF27. Planta.

[bib12] Bruno M., Hofmann M., Vermathen M., Alder A., Beyer P., Al-Babili S. (2014). On the substrate- and stereospecificity of the plant carotenoid cleavage dioxygenase 7. FEBS Lett..

[bib13] Chaiwanon J., Wang W., Zhu J.Y., Oh E., Wang Z.Y. (2016). Information integration and communication in plant growth regulation. Cell.

[bib15] Dickinson A.J., Lehner K., Mi J., Jia K.P., Mijar M., Dinneny J., Al-Babili S., Benfey P.N. (2019). β-Cyclocitral is a conserved root growth regulator. Proc. Natl. Acad. Sci. U S A.

[bib16] Fiorilli V., Wang J.W., Bonfante P., Lanfranco L., Al-Babili S. (2019). Apocarotenoids: old and new mediators of the arbuscular mycorrhizal symbiosis. Front. Plant Sci..

[bib17] Fiorilli V., Vallino M., Biselli C., Faccio A., Bagnaresi P., Bonfante P. (2015). Host and non-host roots in rice: cellular and molecular approaches reveal differential responses to arbuscular mycorrhizal fungi. Front. Plant Sci*.*.

[bib18] Gomez-Roldan V., Fermas S., Brewer P.B., Puech-Pages V., Dun E.A., Pillot J.P., Letisse F., Matusova R., Danoun S., Portais J.C. (2008). Strigolactone inhibition of shoot branching. Nature.

[bib19] Guerrieri A., Dong L., Bouwmeester H.J. (2019). Role and exploitation of underground chemical signaling in plants. Pest Manag. Sci..

[bib20] Gutjahr C., Parniske M. (2013). Cell and developmental biology of arbuscular mycorrhiza symbiosis. Annu. Rev. Cell Dev. Biol..

[bib21] Jamil M., Kountche B.A., Haider I., Guo X., Ntui V.O., Jia K.-P., Ali S., Hameed U.S., Nakamura H., Lyu Y. (2018). Methyl phenlactonoates are efficient strigolactone analogs with simple structure. J. Exp. Bot..

[bib22] Jamil M., Kountche B.A., Haider I., Wang J.Y., Aldossary F., Zarban R.A., Jia K.P., Yonli D., Shahul Hameed U.F., Takahashi I. (2019). Methylation at the C-3'in D-ring of strigolactone analogs reduces biological activity in root parasitic plants and rice. Front. Plant Sci..

[bib23] Jamil M., Kountche B.A., Wang J.Y., Haider I., Jia K.-P., Takahashi I., Ota T., Asami T., Al-Babili S. (2020). A new series of carlactonoic acid based strigolactone analogs for fundamental and applied research. Front. Plant Sci..

[bib24] Jia K.P., Kountche B.A., Jamil M., Guo X.J., Ntui V.O., Rufenacht A., Rochange S., Al-Babili S. (2016). Nitro-phenlactone, a carlactone analog with pleiotropic strigolactone activities. Mol. Plant.

[bib25] Jia K.P., Baz L., Al-Babili S. (2018). From carotenoids to strigolactones. J. Exp. Bot..

[bib26] Jia K.P., Dickinson A.J., Mi J., Cui G., Kharbatia N.M., Guo X., Sugiono E., Aranda M., Blilou I., Rueping M. (2019). Anchorene is a carotenoid-derived regulatory metabolite required for anchor root formation in *Arabidopsis*. Sci. Adv..

[bib27] Koprna R., De Diego N., Dundálková L., Spíchal L. (2016). Use of cytokinins as agrochemicals. Bioorg. Med. Chem..

[bib28] Kountche B.,A., Jamil M., Yonli D., Nikiema M.P., Blanco-Ania D., Asami T., Zwanenburg B., Al-Babili S. (2019). Suicidal germination as a control strategy for *Striga hermonthica* (Benth.) in smallholder farms of sub-Saharan Africa. Plants People Planet..

[bib29] Lanfranco L., Fiorilli V., Venice F., Bonfante P. (2018). Strigolactones cross the kingdoms: plants, fungi, and bacteria in the arbuscular mycorrhizal symbiosis. J. Exp. Bot..

[bib30] Moise A.R., Al-Babili S., Wurtzel E.T. (2014). Mechanistic aspects of carotenoid biosynthesis. Chem. Rev..

[bib31] Parker C. (2012). Parasitic weeds: a world challenge. Weed Sci..

[bib32] Pennisi E. (2010). Armed and dangerous. Science.

[bib33] Rigal A., Ma Q., Robert S. (2014). Unraveling plant hormone signaling through the use of small molecules. Front. Plant Sci..

[bib34] Samejima H., Babiker A.G., Takikawa H., Sasaki M., Sugimoto Y. (2016). Practicality of the suicidal germination approach for controlling *Striga hermonthica*. Pest Manag. Sci..

[bib35] Screpanti C., Fonné-Pfister R., Lumbroso A., Rendine S., Lachia M., De Mesmaeker A. (2016). Strigolactone derivatives for potential crop enhancement applications. Bioorg. Med. Chem. Lett..

[bib36] Takeuchi J., Jiang K., Hirabayashi K., Imamura Y., Wu Y., Xu Y., Miyakawa T., Nakamura H., Tanokura M., Asami T. (2018). Rationally designed strigolactone analogs as antagonists of the D14 receptor. Plant Cell Physiol..

[bib37] Thind B.B., Edwards J.P. (1986). Laboratory evaluation of the juvenile hormone analogue fenoxycarb against some insecticide-susceptible and resistant stored product beetles. J. Stored Prod. Res..

[bib38] Umehara M., Hanada A., Yoshida S., Akiyama K., Arite T., Takeda-Kamiya N. (2008). Inhibition of shoot branching by new terpenoid plant hormones. Nature.

[bib39] Vaidya A.S., Helander J.D.M., Peterson F.C., Elzinga D., Dejonghe W., Kaundal A., Park S.Y., Xing Z., Mega R., Takeuchi J. (2019). Dynamic control of plant water use using designed ABA receptor agonists. Science.

[bib40] Vurro M., Prandi C., Baroccio F. (2016). Strigolactones: how far is their commercial use for agricultural purposes?. Pest Manag. Sci..

[bib41] Wakabayashi T., Hamana M., Mori M., Akiyama R., Ueno K., Osakabe K., Osakabe Y., Suzuki H., Takikawa H., Mizutani M. (2019). Direct conversion of carlactonoic acid to orobanchol by cytochrome P450 CYP722C in strigolactone biosynthesis. Sci. Adv..

[bib42] Wang J.Y., Haider I., Jamil M., Fiorilli V., Saito Y., Mi J., Baz L., Kountche B.A., Jia K.P., Guo X. (2019). The apocarotenoid metabolite zaxinone regulates growth and strigolactone biosynthesis in rice. Nat. Commun..

[bib43] Waters M.T., Gutjahr C., Bennett T., Nelson D.C. (2017). Strigolactone signaling and evolution. Annu. Rev. Plant Biol..

[bib44] Xie X., Yoneyama K. (2010). The strigolactone story. Annu. Rev. Phytopathol..

[bib45] Yoneyama K., Mori N., Sato T., Yoda A., Xie X., Okamoto M., Iwanaga M., Ohnishi T., Nishiwaki H., Asami T. (2018). Conversion of carlactone to carlactonoic acid is a conserved function of MAX1 homologs in strigolactone biosynthesis. New Phytol..

[bib46] Zhang Y., van Dijk A.D., Scaffidi A., Flematti G.R., Hofmann M., Charnikhova T., Verstappen F., Hepworth J., van der Krol S., Leyser O. (2014). Rice cytochrome P450 MAX1 homologs catalyze distinct steps in strigolactone biosynthesis. Nat. Chem. Biol..

