# Supplementary material for: Efficient Mimics for Elucidating Zaxinone Biology and Promoting Agricultural Applications
Source: Mol Plant. 2020 Nov 2;13(11):1654–61. doi: 10.1016/j.molp.2020.08.009 (PMC7656291; doi:10.1016/j.molp.2020.08.009)
Supplement: Document S1. Supplemental Methods, Supplemental Figures 1–14, Supplemental Table 1 and Supplemental References [file mmc1.pdf]

Molecular Plant, Volume *13*

Supplemental Information

Efficient Mimics for Elucidating Zaxinone Biology and Promoting Agricultural Applications

Jian You Wang, Muhammad Jamil, Pei-Yu Lin, Tsuyoshi Ota, Valentina Fiorilli, Mara Novero, Randa A. Zarban, Boubacar A. Kountche, Ikuo Takahashi, Claudio Martínez, Luisa Lanfranco, Paola Bonfante, Angel R. de Lera, Tadao Asami, and Salim Al-Babili

Efficient Mimics for Elucidating Zaxinone Biology and Promoting Agricultural Applications

Jian You Wang, Muhammad Jamil, Pei-Yu Lin, Tsuyoshi Ota, Valentina Fiorilli, Mara Novero, Randa A. Zarban, Boubacar A. Kountche, Ikuo Takahashi, Claudio Martínez, Luisa Lanfranco, Paola Bonfante, Angel R. de Lera, Tadao Asami and Salim Al-Babili

Supplemental Document 1

Supplemental Methods

Plant material and growth conditions

Rice plants were grown according to Wang et al., 2019 under controlled conditions (a 12 h photoperiod, 200-µmol photons m^{-2} s^{-1} and day/night temperature of 27/25 ^{o}C) with half-strength modified Hoagland nutrient solution, which consisted of 5.6 mM NH_4NO_3, 0.8 mM $MgSO_4{\cdot}7H_2O$, 0.8 mM K_2SO_4, 0.18 mM $FeSO_4{\cdot}7H_2O$, 0.18 mM $Na_2EDTA{\cdot}2H_2O$, 1.6 mM $CaCl_2{\cdot}2H_2O$, 0.8 mM KNO_3, 0.023 mM H_3BO_3, 0.0045 mM $MnCl_2{\cdot}4H_2O$, 0.0003 mM $CuSO_4{\cdot}5H_2O$, 0.0015 mM $ZnCl_2$, 0.0001 mM $Na_2MoO_4{\cdot}2H_2O$ and with or without 0.4 mM $K_2HPO_4{\cdot}2H_2O$, resulting in the +Pi and -Pi conditions, respectively with adjusted pH to 5.8.

For treatment of hydroponically grown plants with MiZax, we exposed one week-old seedlings were grown hydroponically containing 2.5 µM compounds (dissolved in 0.1% acetone) for 21 days. The solution was changed every other day, adding the chemical at each renewal.

Rhizotron (48cm x 24 cm x 5cm) experiments were conducted as following: three-day-old seedlings were grown in soil and treated with the compounds at a 5 µM concentration and dissolved in 1 µL/mL emulsifier (Atlas G1086, CRODA, Gouda, The Netherlands; prepared in cyclohexanone according to Kountche et al., 2019) with half-strength modified Hoagland nutrient solution for two weeks. The solution was added twice per week. Root surface area was analyzed with the ImageJ software.

For SL and transcript analysis, one-week-old seedlings were transferred into 50 mL falcon tubes (two seedlings per tube), containing half-strength Hoagland nutrient solution with $K_2HPO_4.2H_2O$ (+Pi), for one week. Rice seedlings were then subjected to phosphate deficiency (-Pi) for another one week. On the day of root exudates collection, rice seedlings were first treated with 5 µM compounds for 6 h, and then root exudates and root tissues were collected for analysis separately.

Synthesis of enantiopure zaxinone and analogues

The synthesis of the (iso)-zaxinone compounds followed the general strategies used for the synthesis of retinoids and carotenoids as described in Supplemental Document S2.

Synthetic methods for zaxinone-mimics (MiZax)

The chemical synthesis procedures and NMR characterization of MiZax are listed in Supplemental Document S3.

Quantitative analysis of SLs in rice root exudates and root tissues

Quantification of SLs in rice root exudates was followed by the protocol described previously by Wang et al., 2019. Briefly, 50 mL of root exudates spiked with 0.672 ng of D_6–5-deoxystrigol, was brought on a 500 mg/3 mL fast SPE C18 column preconditioned with 6 mL of methanol and 6 mL of water. After washing with 6 mL of water, SLs were eluted with 5 mL of acetone. The SLs fraction (acetone-water solution) was concentrated to SL aqueous solution (~1 mL), followed by the extraction with 1 mL of ethyl acetate. 750 µL of 4-deoxyorobanchol enriched organic phase was then transferred to 1.5 mL tube and evaporated to dryness under vacuum. The dried extract was dissolved in 100 µL of acetonitrile: water (25:75, v:v) and filtered through a 0.22 µm filter for LC-MS/MS analysis.

Quantification of SLs in rice root was followed the protocol described previously by Wang et al., 2019. 25 mg of lyophilized plant spiked with 0.672 ng of D_6–5-deoxystrigol were extracted twice with 2 mL of ethyl acetate in an ultrasound bath (Branson 3510 ultrasonic bath) for 15 min, followed by centrifugation for 8 min at 3800 rpm at 4 °C. The two supernatants were combined and dried under vacuum. The residue was dissolved in 100 µL of ethyl acetate and 2 mL of hexane following a Silica gel SPE column (500 mg/3 mL) purification and elution with 3 mL of ethyl acetate and evaporated to dryness under vacuum. The residue was re-dissolved in 200 µL of

acetonitrile: water (25:75, v:v) and filtered through a 0.22 μm filter for LC-MS/MS analysis. The characteristic MRM transitions (precursor ion → product ion) were 331→216, 331→97 for 4-deoxyorobanchol; 347.1→233, 347.1→97 for orobanchol; 337→222, 337→97 for D_6-5-deoxystrigol.

Striga hermonthica **seed germination bioassays**

Striga hermonthica seed germination bioassays were conducted according to Jamil et al., 2012. Briefly, the root exudates collected from treated and untreated rice, was applied at 50 μL on each six discs containing preconditioned *Striga* seeds. Sterile MilliQ water and standard SL analog GR24 (2.5 μM) were included as a negative and positive control, respectively. After application, *Striga* seeds were incubated in dark at 30 ºC for 24 hours. Germination (seeds with radicle emerging through seed coat) was scored under a binocular microscope, and germination rate (%) was calculated.

Striga hermonthica **infection in rice**

Striga pot experiment was adapted from Kountche et al., 2019. About 20 mg *Striga* seeds were thoroughly mixed in 1.5 L sand and soil mixture (1:1) and added in 3 L perforated plastic pot containing 0.5 L clean soil in the bottom. The pots were kept in greenhouse at 35 °C under moisture to precondition *Striga* seeds for 10 days. On the 11th day, five one-week old rice seedlings (cv IAC165) were planted in each pot. After three days of rice planting, each pot was irrigated with 250 mL phosphorus deficient Hoaglands nutrient solution, followed after four hours by irrigation with 25 mL formulated MiZax3, MiZax5 and Zaxinone (100 μM) were sprayed. Next day, each pot was again supplied with 250 mL nutrient solution to adjust the final concentration of each compound to 5 μM and to move the compound to rice seedlings. Compounds were applied twice a week up to four weeks, and after three weeks the number of emerged *Striga* plants in each pot were counted.

Gene expression analysis

Rice roots were ground and homogenized in liquid nitrogen, and total RNA was isolated using a Direct-zol RNA Miniprep Plus Kit following the manufacturer's instructions (ZYMO RESEARCH; USA). cDNA was synthesized from 1 µg of total RNA using iScript cDNA Synthesis Kit (BIO-RAD Laboratories, Inc, 2000 Alfred Nobel Drive, Hercules, CA; USA). The

gene expression level was detected by real-time quantitative RT-PCR (qRT-PCR) which was performed using SYBR Green Master Mix (Applied Biosystems; www.lifetechnologies.com) in a CFX384 Touch™ Real-Time PCR Detection System (BIO-RAD Laboratories, Inc, 2000 Alfred Nobel Drive, Hercules, CA; USA). Primers used for qRT-PCR analysis are listed in Supplemental Table1. The gene expression level was calculated by normalization of a housekeeping gene in rice, Ubiquitin (OsUBQ). The relative gene expression level was calculated according to $2^{-\Delta\Delta}$CT method (Livak and Schmittgen, 2001).

Chemical stability

MiZax3 and MiZax5 were tested for their chemical stability at 21±1°C with pH 5.0-6.0 aqueous solution following the protocol described by Jamil et al., 2019. 50 µL of each compound solution (1mg $mL^{-}1$) was prepared with 175 µL ethanol and 750 µL Mili-Q water. Thereafter, 25 µL Indanol (1mg $mL^{-}1$, internal standard) was spiked in 975 µL previous prepared solution. The degradation was monitored by UPLC using an Agilent HPLC ZORBAX Eclipse XDB-C_{18} column (3.5 µm, 4.6 × 150 mm), eluted first by 5% acetonitrile in water for 0.5min then by a gradient flow from 5% to 100% acetonitrile within 18 min, and by 100% acetonitrile for 5 min. The column was operated at 40°C at 0.35 mL $min^{-}1$ flow rate. Compounds eluted from the column were detected, and the relative quantity of non-degraded amount was calculated with Indanol.

Detection of MiZax in rice root and shoot tissues

Two-week-old rice plants were treated with the compounds at a 10 µM concentration for 6 h. Plant fresh tissues were then washed and grinded under liquid nitrogen. Separated root and shoot tissues were extracted with 1.5 mL of ethyl acetate in an ultrasound bath (Branson 3510 ultrasonic bath) for 15 min, followed by centrifugation for 8 min at 3800 rpm at 4 °C. The supernatants were transferred to new Eppendorf tubes and dried under vacuum. The residue was re-dissolved in 150 µL of acetonitrile and filtered through a 0.22 µm filter for LC-MS/MS analysis. The analysis of MiZax in rice tissues was performed by using UHPLC- Triple-Stage Quadrupole Mass Spectrometer (Thermo Scientific™ Altis™) with MRM mode. Chromatographic separation was achieved on a ZORBAX Eclipse plus C_{18} column (150 × 2.1 mm; 3.5 µm; Agilent) with mobile phases consisting of water (A) and acetonitrile (B), both containing 0.1% formic acid, and the following linear gradient (flow rate, 0.6 ml/min): 0–6 min, 5%–100 % B, 6–17.5 min, 100 % B,

followed by washing with 100 % B and equilibration with 5 % B. The injection volume was 10 µl, and the column temperature was maintained at 35 °C for each run. The MS parameters were set as follows: positive ion mode, ion source of H-ESI, ion spray voltage of 5000 V, sheath gas of 40 arb, aux gas of 15 arb, sweep gas of 20 arb, ion transfer tube gas temperature of 350 °C, vaporizer temperature of 350 °C, collision energy of 17 eV, CID gas of 2 mTorr. The characteristic MRM transitions (precursor ion → product ion) were 255.1→197.05, 255.1→133.1 for MiZax2; 269.1→211.1, 269.1→133.1 for MiZax3; 289.1→247.2, 289.1→226.9 for MiZax4; 303.1→261.1, 303.1→216.1 for MiZax5

Effect of zaxinone-mimics on *Gigaspora margarita* spores germination

Spores were sterilized in a solution of Streptomycine sulphate (0.03% W/V) and Chloramine T (3% W/V) and germinated in 200 µL of zaxinone, MiZax3, and MiZax5 at 5 µM or 50 nM, GR24 $10^{-}7$ M or upon a mixed solution of MiZax3 (5 µM) + GR24 (10 nM) or MiZax5 (5 µM) + GR24 (10 nM) (12.5 µL acetone in 25 mL water). For each treatments 96 sterilized spores were placed individually in the wells of a multi-well plate and treated with freshly prepared solutions at the beginning of the experiment . Spores were germinated in the dark at 30°C and the germination rate was evaluated after 3 days.

Plant and fungal material and treatments

Seeds of WT plants cv Nipponbare were germinated in pots containing sand and incubated for 10 days in a growth chamber under a 14 h light (23°C)/10 h dark (21°C). Plants used for mycorrhization were inoculated with *Funneliformis mosseae* (BEG 12, MycAgroLab, France). Fungal inoculum (25%) were mixed with sterile quartz sand and used for colonization. Plants were watered with a modified Long-Ashton (LA) solution containing 3.2 µM $Na_2HPO_4 \cdot 12\ H_2O$ (Hewitt, 1966). Ten days after mycorrhizal inoculation, a set of WT plants were treated with 5 µM or 50 nM MiZax3 or MiZax5, by applying molecules twice a week directly in the nutrient solution.

Wild-type and WT treated mycorrhizal roots were stained with 0.1 % cotton blue in lactic acid and the estimation of mycorrhizal parameters was performed by Trouvelot method (Trouvelot et al., 1986). Four parameters were considered: F%, percentage of segments showing internal colonization (frequency of mycorrhization); M%, average percentage of colonization of root segments (intensity of mycorrhization); a%, percentage of arbuscules within infected areas; A%,

percentage of arbuscules in the whole root system. For the molecular analyses, roots were immediately frozen in liquid nitrogen and stored at -80 °C.

Gene expression analysis of mycorrhizal plants

Total genomic DNA was extracted from *F. mosseae* sporocarps and *O. sativa* roots using the DNeasy Plant Mini Kit (Qiagen), according to the manufacturer's instructions. Genomic DNAs were used to test each primers pair designed for real-time PCR to exclude cross hybridization. Total RNA was extracted from rice roots using the Plant RNeasy Kit (Qiagen), according to the manufacturer's instructions. Samples were treated with TURBO™ DNase (Ambion) according to the manufacturer's instructions. The RNA samples were routinely checked for DNA contamination by means of PCR analysis, using primers for OsRubQ1 (Güimil et al. 2005). For single-strand cDNA synthesis about 1000 ng of total RNA was denatured at 65°C for 5 min and then reverse-transcribed at 25°C for 10 min, 42°C for 50 min, and 70°C for 15 min. The reaction was carried out in a final volume of 20 μL containing 10 μM random primers, 0.5 mM dNTPs, 4 μL 5X buffer, 2 μL 0.1 M DTT and 1 μL Super-Script II (Invitrogen). Quantitative RT-PCR (qRT-PCR) was performed using a Rotor-Gene Q 5plex HRM Platform (Qiagen). Each PCR reaction was carried out in a total volume of 15 μL containing 2 μL diluted cDNA (about 10 ng), 7.5 μL 2X SYBR Green Reaction Mix, and 2.75 μL of each primer (3 μM). The following PCR program was used: 95°C for 90 sec, 40 cycles of 95°C for 15 sec, 60°C for 30 sec. A melting curve (80 steps with a heating rate of 0.5°C per 10 sec and a continuous fluorescence measurement) was recorded at the end of each run to exclude the generation of non-specific PCR products. All reactions were performed on at least three biological and three technical replicates. Baseline range and take off values were automatically calculated using Rotor-Gene Q 5plex software. Transcript level of *OsPT11* (Güimil et al. 2005); *OsLysM* (Fiorilli et al., 2015) and *Fm18S* (Balestrini et al., 2007) were normalized using OsRubQ1 housekeeping gene (Güimil et al. 2005). Primers used for qRT-PCR analysis are listed in Supplemental Table1. Only take off values leading to a Ct mean with a standard deviation below of 0.5 were considered. Statistical tests were carried out through one-way analysis of variance (one-way ANOVA) and Tukey's post hoc test, using a probability level of $p<0.05$. All statistical elaborations were performed using PAST statistical (version 2.16; Hammer et al. 2001). To analyze the fungal intraradical morphology root apparata were stained in

Cotton Blue (0,1% W/V) in lactic acid, cut in pieces 1cm long and observed under an optical microscope.

Supplemental Figures

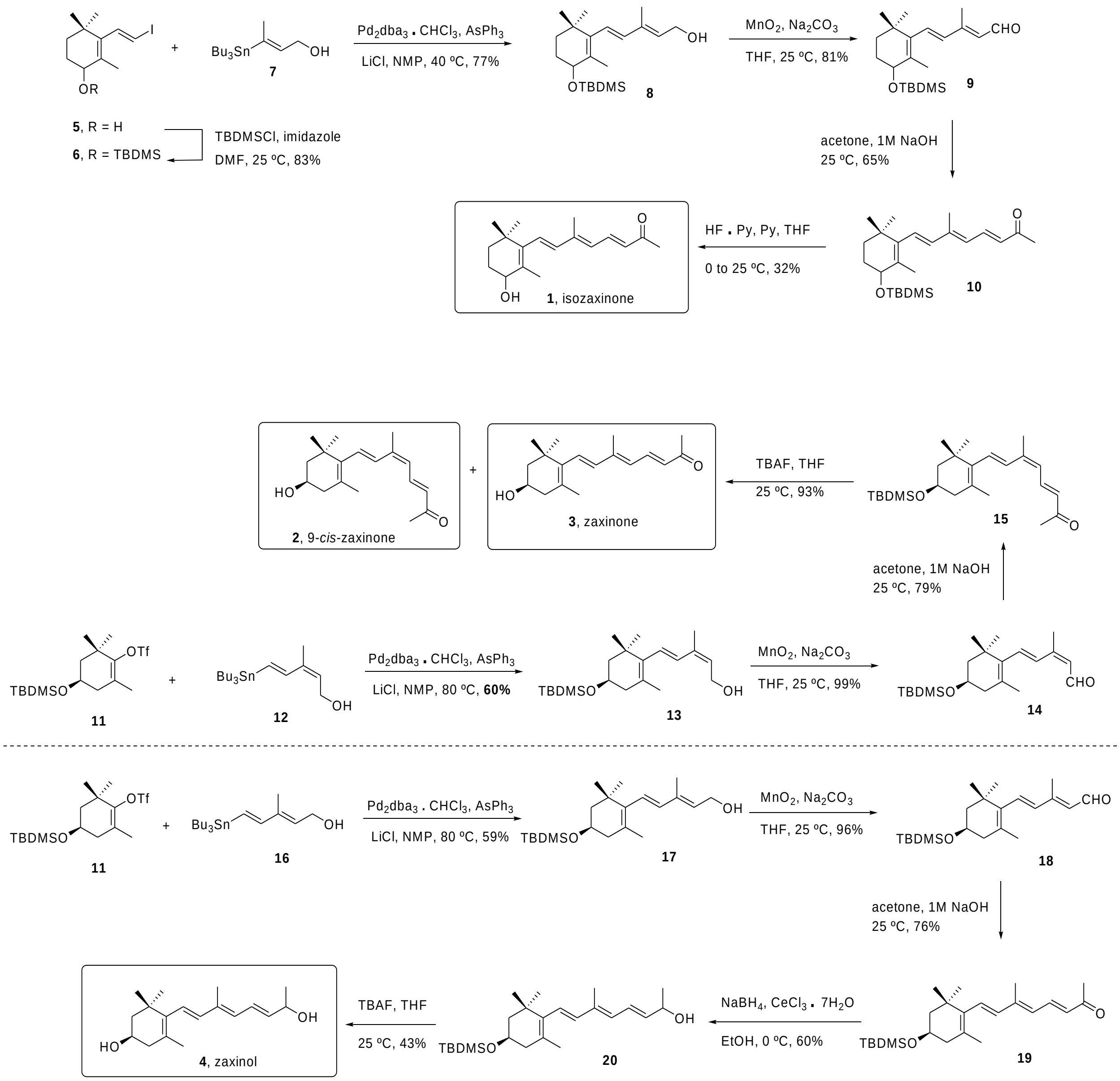

Supplemental Figure 1. Chemical Synthesis of Zaxinone.

The synthesis of the zaxinone and its analogs followed the general strategies used for the synthesis of retinoids and carotenoids as described in Supplemental Document S2.

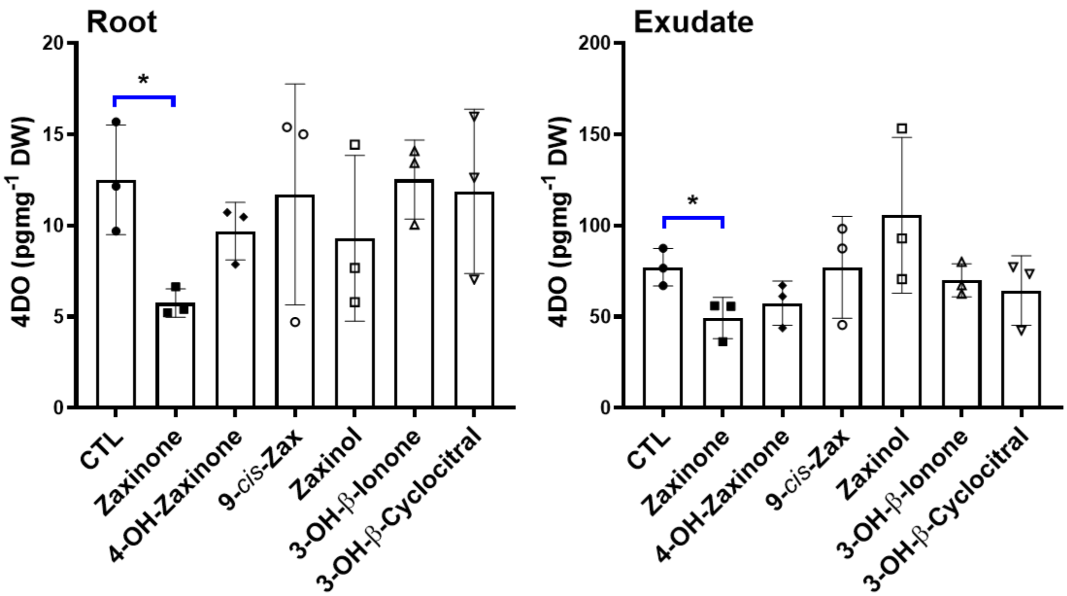

Supplemental Figure 2. Effect of Apocarotenoids on SL Content in Root Tissues and Exudates.

SL, 4-deoxyorobanchol (4-DO), quantification in wild-type roots and root exudates in response to zaxinone, 4-OH-zaxinone, 9-*cis*-Zaxinone, zaxinol, 3-OH-β-Ionone, and 3-OH-β-Cyclocitral at 5 µM under Pi starvation. Bars represent mean ± SD; n=3 biological replicates; Statistical analysis was performed using a two-tailed t-test. *, $P < 0.05$.

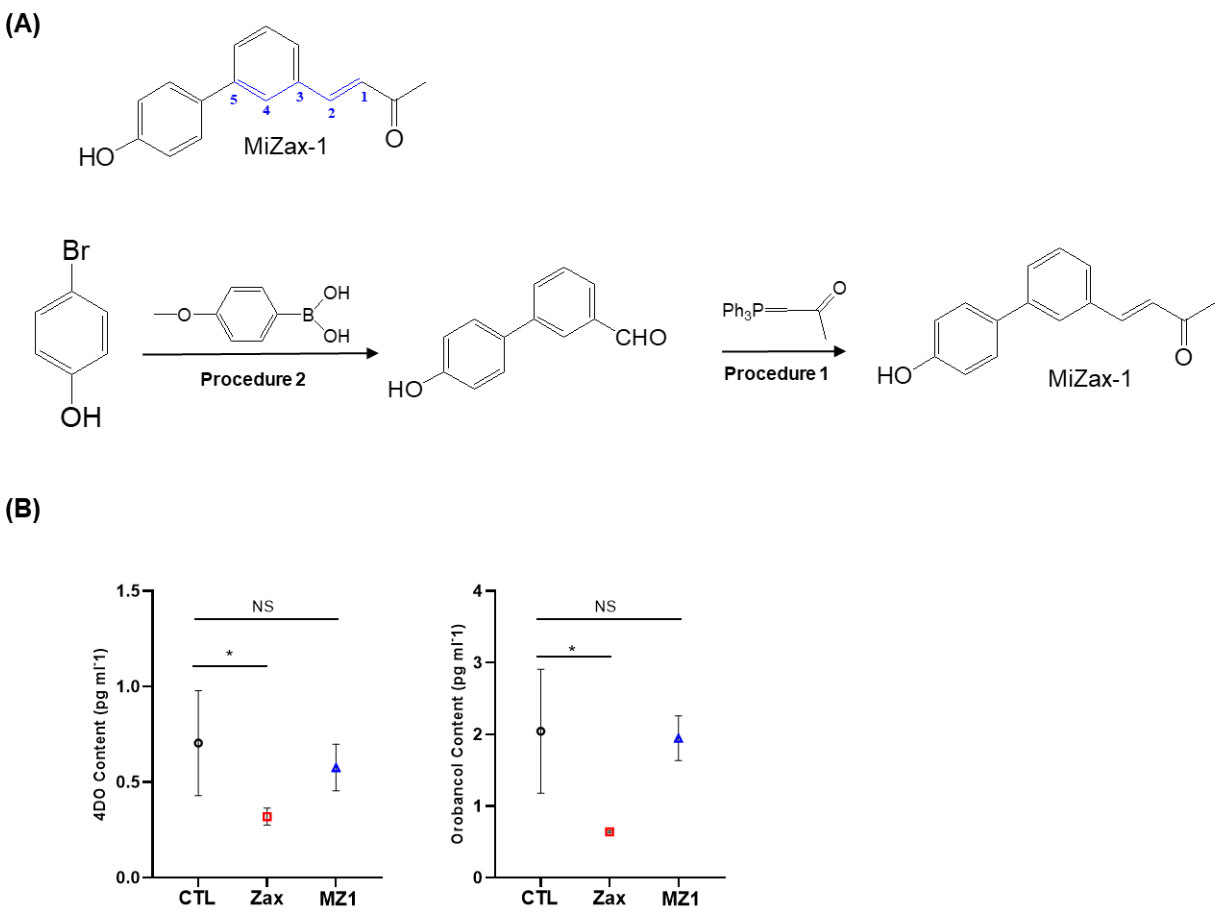

Supplemental Figure 3. Effect of MiZax1 on Rice SL Release.

(A) Chemical structure of MiZax1 and the corresponding synthesis scheme. Numbers in blue indicate the distance between phenyl ring and the ketone group.

(B) Quantification of SLs, 4-deoxyorobanchol (4-DO) and Orobanchol (Oro) , in wild-type root exudates in response to zaxinone, and MiZax1 at 5 µM under Pi starvation. Bars represent mean ± SD; *n*=3 biological replicates; statistical analysis was performed using a two-tailed *t*-test. *, P < 0.05. NS, non-significant; CTL, Control; Zax, Zaxinone; MZ1, MiZax1

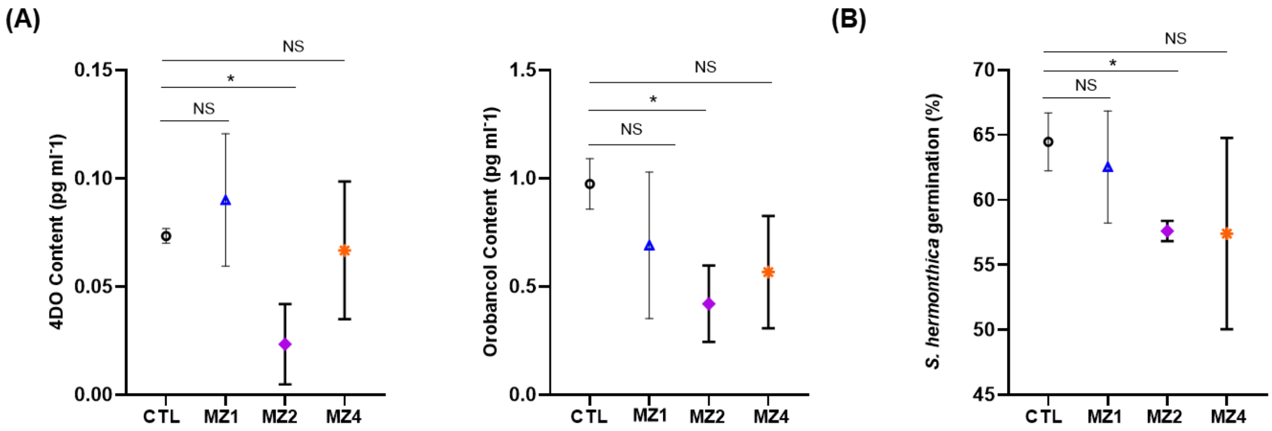

Supplementary Figure 4. Comparison of MiZax on rice SL release.

(A) Quantification of SLs, 4-deoxyorobanchol (4-DO) and Orobanchol (Oro) , in wild-type root exudates in response to MiZax1, MiZax2, or MiZax4 at 5 µM under Pi starvation.

(B) *Striga* seed germination activity of rice root exudates isolated from plants treated with MiZax1, MiZax2, or MiZax4 at 5 µM under Pi starvation.

Bars represent mean ± SD; *n*=3 biological replicates; statistical analysis was performed using *t*-test. *, P < 0.05. NS, non-significant;CTL, Control; Zax, Zaxinone; MZ1, MiZax1; MZ2, MiZax2; MZ4, MiZax4

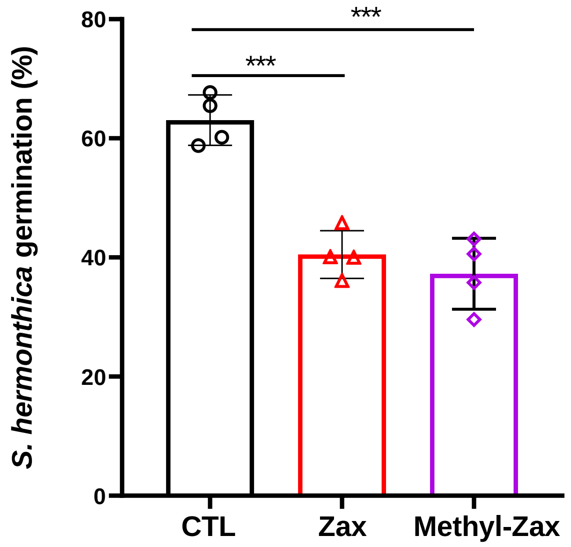

Supplementary Figure 5. Bioactivity of Methyl-Zax in rice.

Striga seed germination activity of rice root exudates isolated from plants treated with Zaxinone, Methyl-Zaxinone at 5 µM under Pi starvation. Bars represent mean ± SD; *n*=4 biological replicates; statistical analysis was performed using *t*-test. ***, P < 0.001. CTL, Control; Zax, Zaxinone; Methyl-Zax, Methyl-Zaxinone.

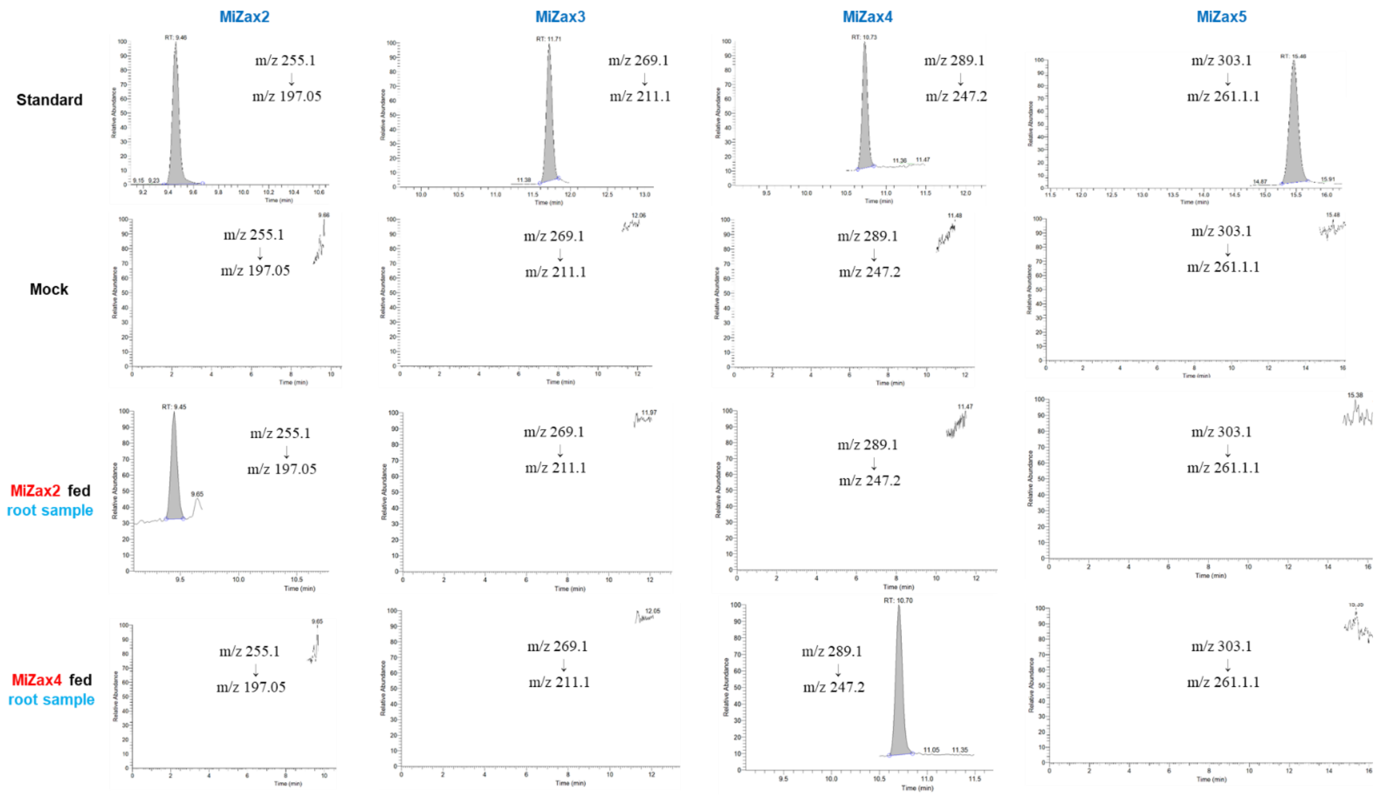

Supplementary Figure 6. LC-MS detection of MiZax2/4 in root tissues.

Roots of rice plants fed with MiZax2 or MiZax4 accumulated both mimics but did not convert them into MiZax3 or MiZax5, respectively.

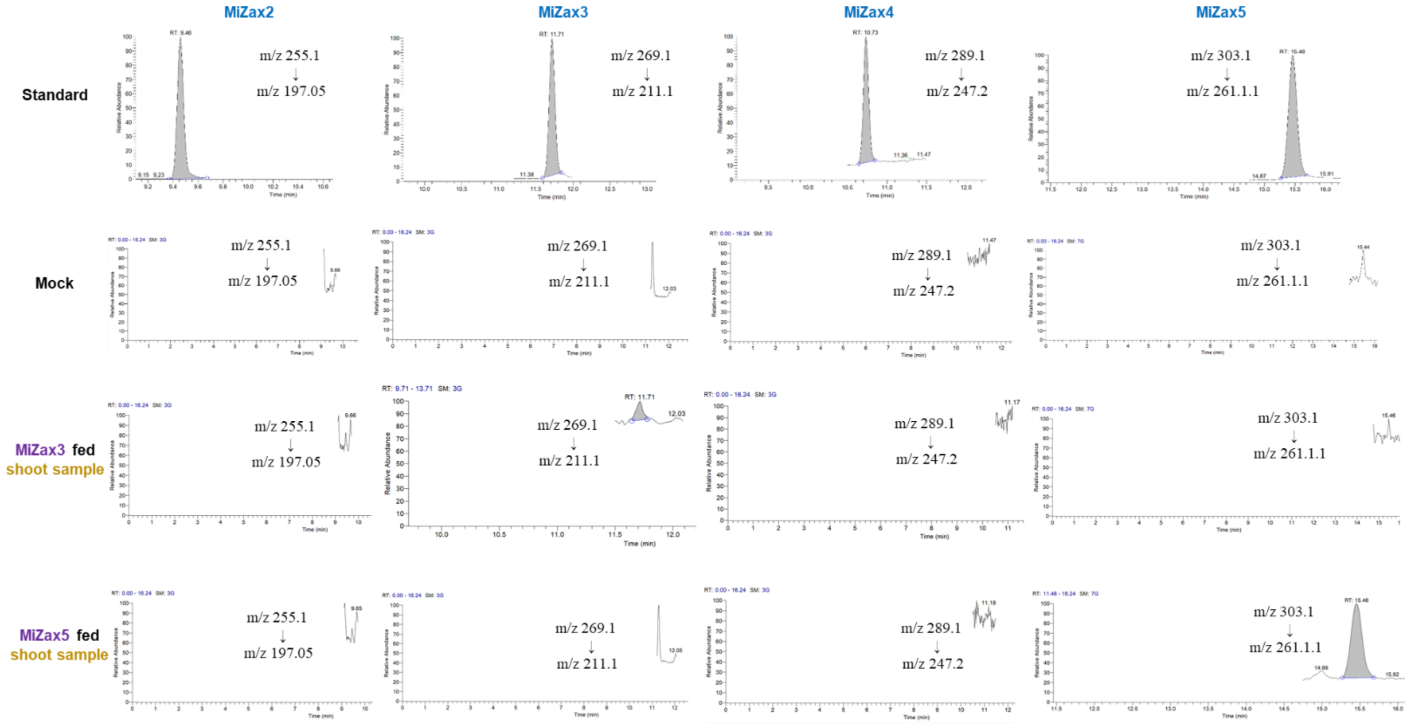

Supplementary Figure 7. LC-MS detection of MiZax3/5 in shoot tissues.

MiZax3 or MiZax5 fed through rice roots is transported to their shoots.

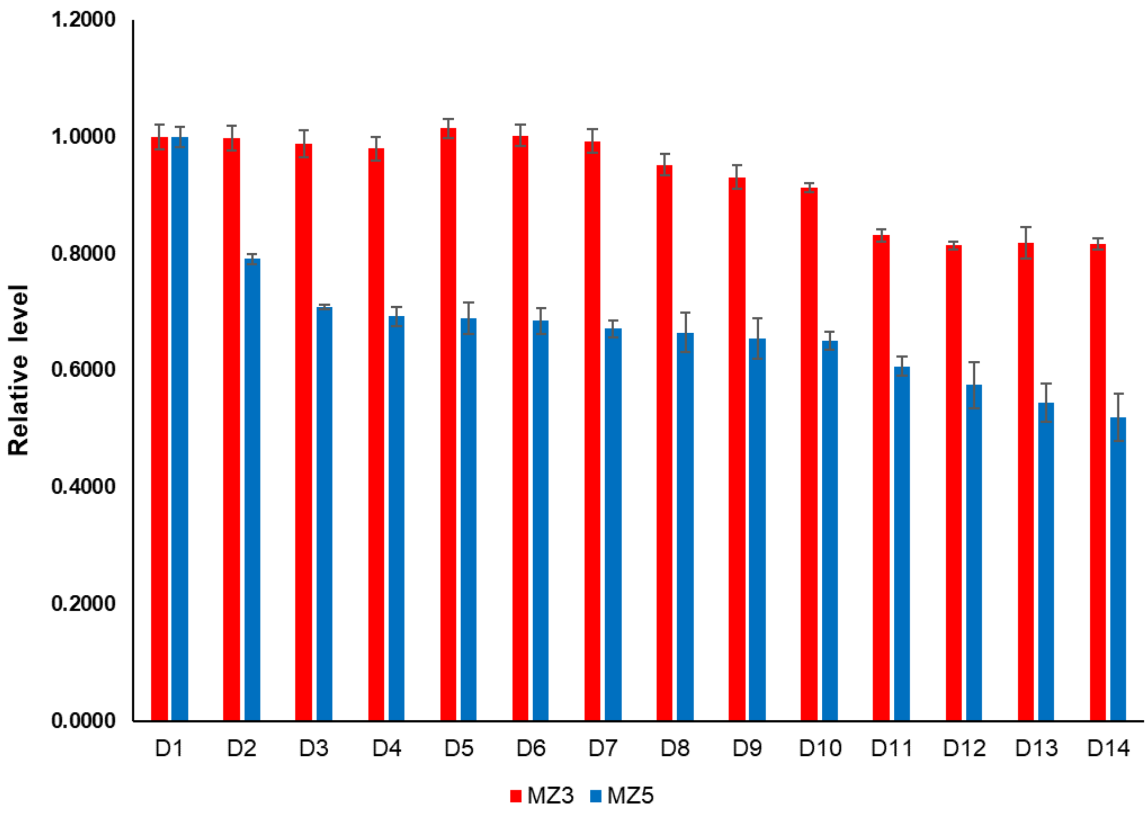

Supplemental Figure 8. Stability of MiZax by HPLC Analysis.

The relative amount of non-degraded analogs was monitored in HPLC for 14 days. Data are means ± SE (n = 3). X-axis: time (days); Y-axis: relative levels. MZ3, MiZax3; MZ5, MiZax5.

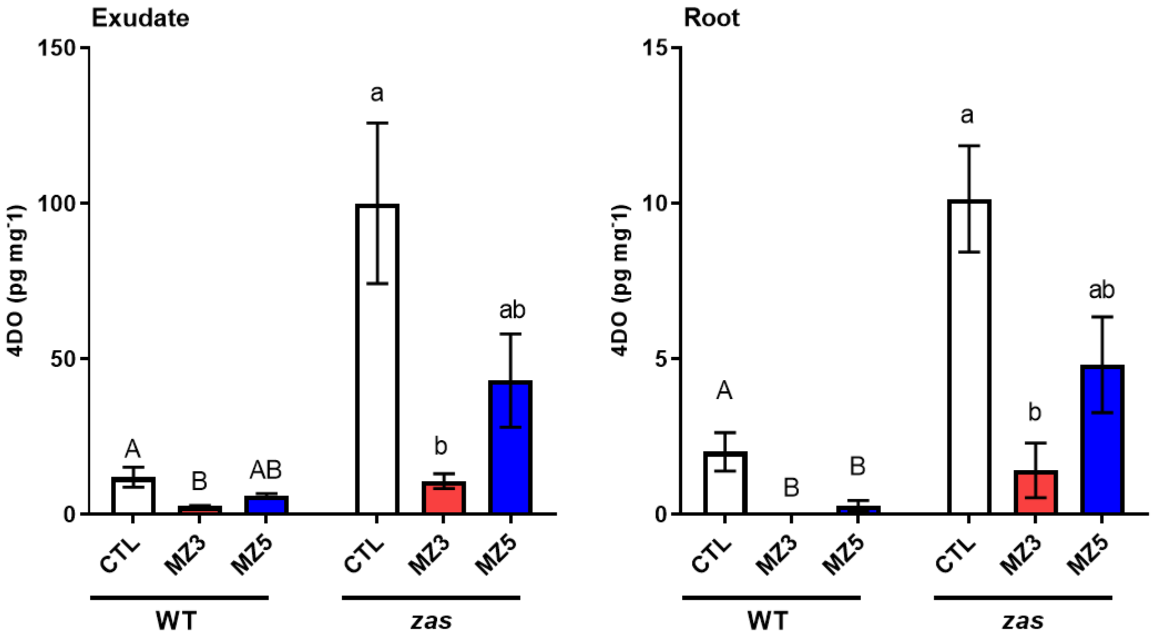

Supplemental Figure 9. Effect of MiZax on SL Content in Root Tissues and Exudates.

SL, 4-deoxyorobanchol (4-DO), quantification in wild-type and *zas* mutant root exudates (left) and roots (right) in response to MZ3 and MZ5 at 5 µM under Pi starvation. Bars represent mean ± SD; *n*=4 biological replicates; statistical analysis was performed using one-way analysis of variance (ANOVA) and Tukey's *post hoc* test. Different letters denote significant differences ($P < 0.05$). CTL, Control; MZ3, MiZax3; MZ5, MiZax5.

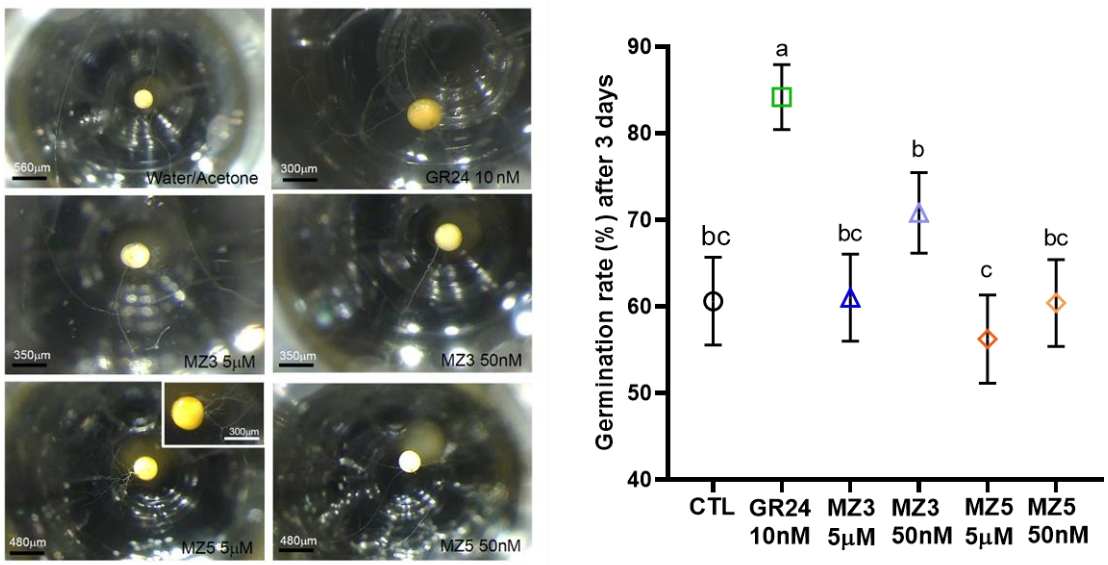

Supplemental Figure 10. Effect of MiZax on *Gigaspora margarita* Spores Germination.

Bars represent mean ± SE; n=96 biological replicates; Data indicated with different letters are statistically different according to the non-parametric Kruskal-Wallis test. Different letters denote significant differences ($P < 0.05$). CTL, Control (acetone); MZ3, MiZax3; MZ5, MiZax5; GR24, a synthetic SL analog.

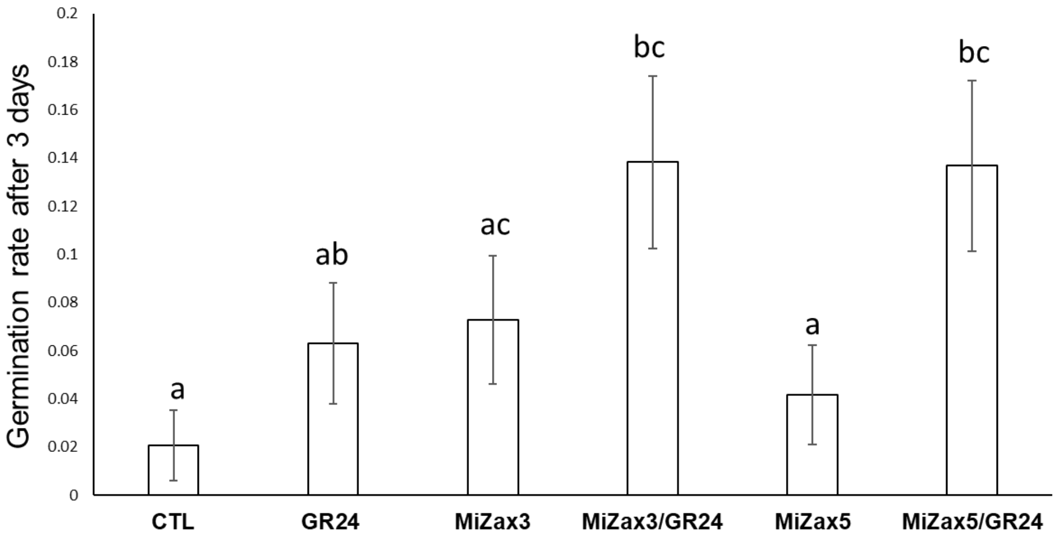

Supplemental Figure 11. Effect of MiZax on SL-mediated *Gigaspora margarita* spore germination. Bars represent mean ± SE; *n*=96 biological replicates; Data indicated with different letters are statistically different according to the non-parametric Kruskal-Wallis test ($p<0.05$). CLT: spores treated with acetone; GR24 (SL analog): spores treated with GR24 (10 nM); MiZax3: spores treated with MiZax3 (5 µM); MiZax3/GR24: spores treated with MiZax3 (5 µM) and GR24 (10 nM); MiZax5: spores treated with MiZax5 (5 µM); MiZax5/GR24: spores treated with MiZax5 (5 µM) and GR24 (10 nM).

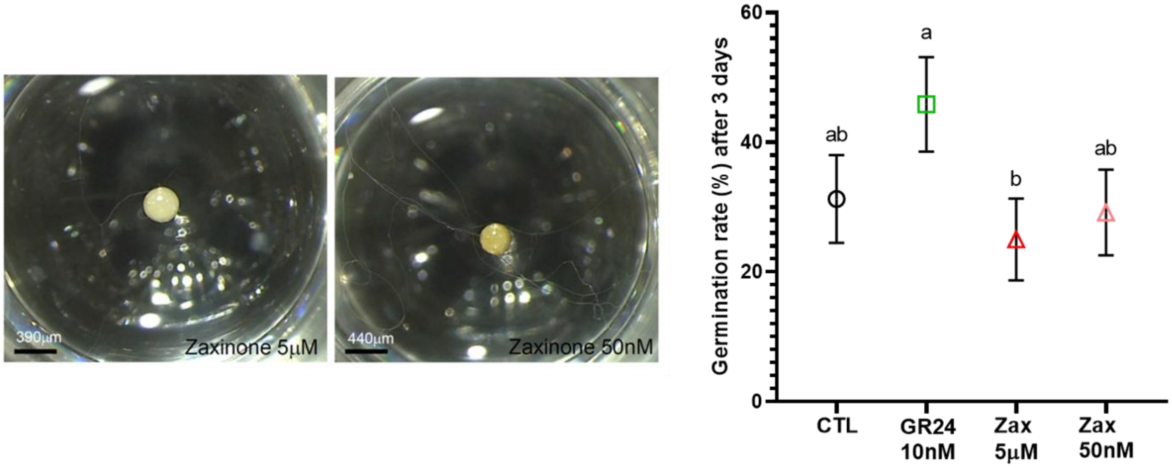

Supplemental Figure 12. Effect of Zaxinone on *Gigaspora margarita* Spores Germination. Bars represent mean ± SE; *n*=96 biological replicates; Data indicated with different letters are statistically different according to the non-parametric Kruskal-Wallis test ($p<0.05$). CTL, Control (acetone); Zax, Zaxinone; GR24, a synthetic SL analog.

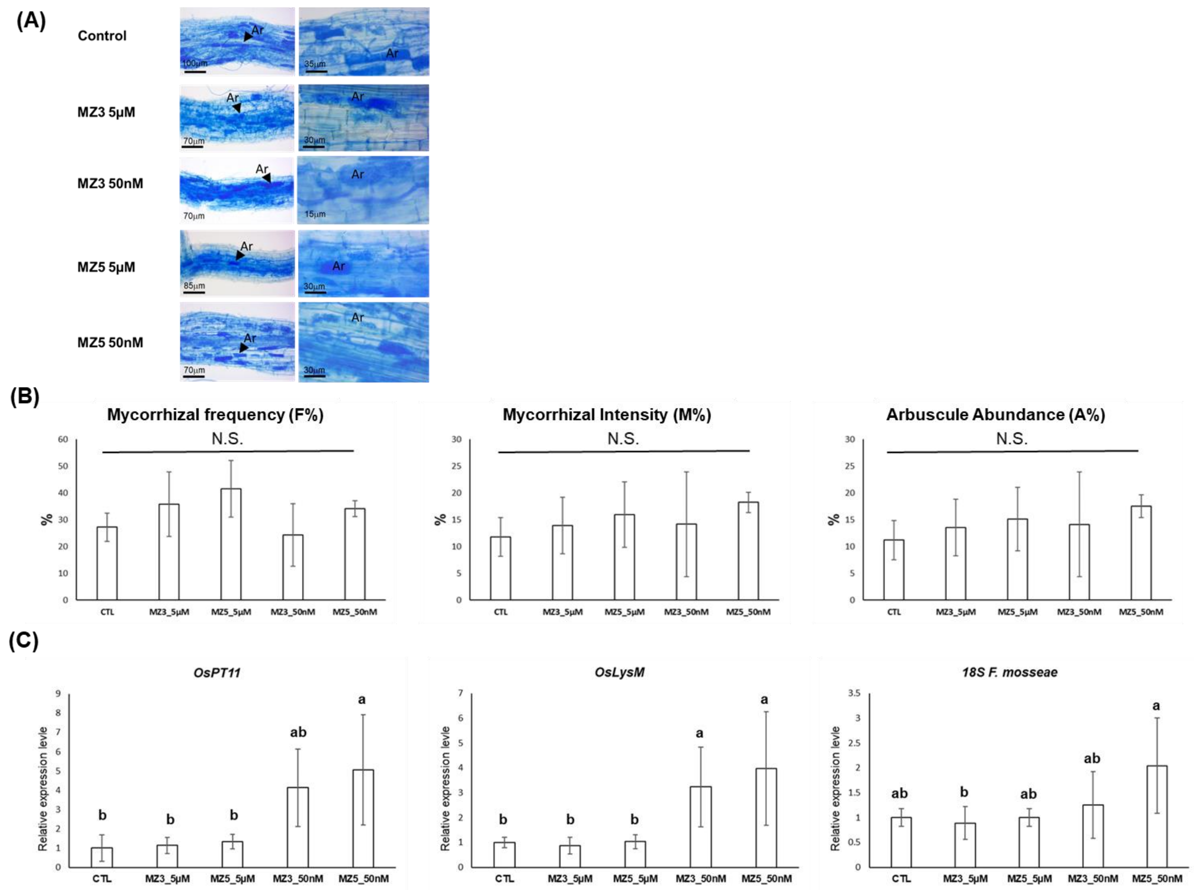

Supplemental Figure 13. Evaluation of MiZax Effects on the AM Symbiosis.

(A) Details of arbuscule morphology in roots stained with cotton blue. CTL, Control; MZ3, MiZax; MZ5, MiZax5. ; Ar, Arbuscule- containing cells.

(B) Mycorrhizal colonization in non-treated and treated plants by the AM fungi *Funneliformis mosseae* at 35 dpi. Degree of colonization expressed as mycorrhizal frequency (F %), intensity (M %) and arbuscule abundance (A %) in the root system of WT plants non treated and treated with 5µM and 50nM Mizax3 or Mizax5. Data are the average of four biological replicates ± SE. CTL, Control; MZ3, MiZax; MZ5, MiZax5; N.S., non-significant.

(C) Molecular evaluation of mycorrhization by qRT-PCR analysis of mRNA abundance of plant AM-responsive genes (*OsPT11*, *OsLysM*) and fungal housekeeping gene (*18S F. mosseae*) on mycorrhizal roots considering the whole root system. Bars represent mean ± SD; n=4 biological replicates. Statistical analysis was performed using one-way analysis of variance (ANOVA) and Tukey's *post hoc* test.

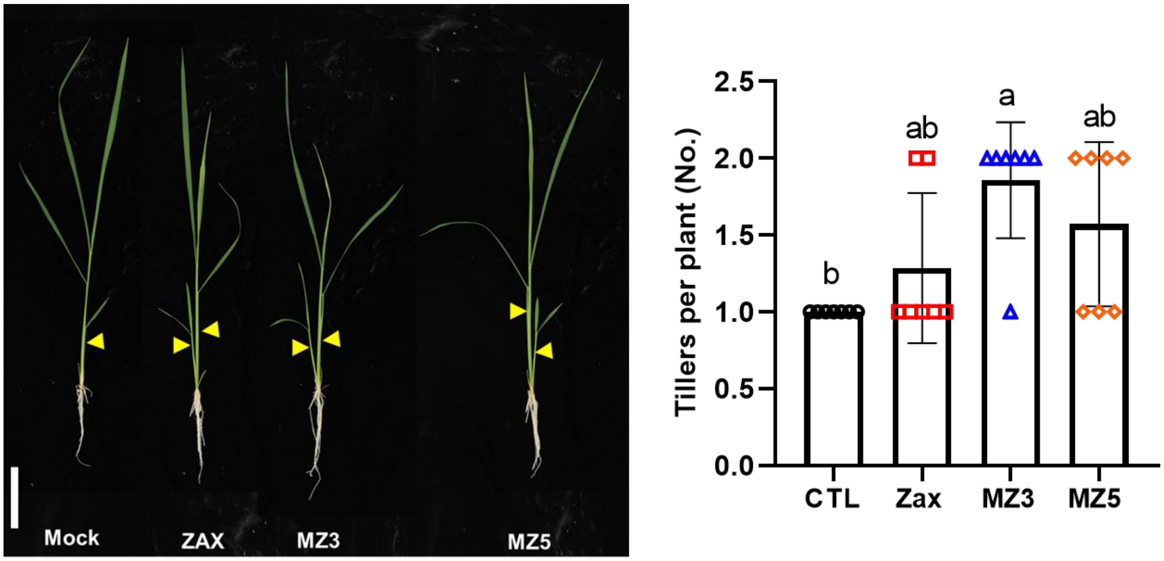

Supplemental Figure 14. Effect of Zaxinone, MZ3, and MZ5 at 2.5 µM on tillers of IAC165 seedlings grown hydroponically.

Tillers are indicated by yellow arrows points. Each data point represents one plant, *n*=7. Data represent mean ± SD. Statistical analysis was performed using one-way analysis of variance (ANOVA) and Tukey's *post hoc* test. Different letters denote significant differences ($P < 0.05$). Scale bars=8 cm. CTL, Control; Zax, Zaxinone; MZ3, MiZax3; MZ5, MiZax5.

Supplemental Table 1. Primer sequences used in this study

Experiment	Primer name	Sequence (5′–3′)
qRT-PCR	OsUbi-Q F	GCCCAAGAAGAAGATCAAGAAC
	OsUbi-Q R	AGATAACAACGGAAGCATAAAAG
	OsD27 F	CTTCCAAGCTACATCCTCAC
	OsD27 R	CCCAACCAACCAAGGAAA
	OsCCD7 F	CAGTCTCCAAGCACAGATG
	OsCCD7 R	GTTCTTTGGCACCTCTAGTT
	OsCCD8b F	TGGCGATATCGATGGTGA
	OsCCD8b R	GACCTCCTCGAACGTCTT
	OsMax1-900 F	ATTGTCAGCGATCCACTTC
	OsMax1-900 R	GCGCCGTTCTTGAAATTG
	OsRubQ1 F	GGGTTCACAAGTCTGCCTATTTG
	OsRubQ1 R	ACGGGACACGACCAAGGA
	OSPt11 F	GAGAAGTTCCCTGCTTCAAGCA
	OSPt11 R	CATATCCCAGATGAGCGTATCATG
	OsLysM F	CGCTGACATGCAACAAGGTG
	OsLysM R	CTTCGCGCAGTTGATGTTTGG
	18SF.m F	CCTTTTGAGCTCGGTCTCGTG
	18SF.m R	TGGTCCGTGTTTCAAGACG

Supplemental References

Balestrini, R., Go´mez-Ariza, J., Lanfranco, L., Bonfante, P. (2007). Laser microdissection reveals that transcripts for five plant and one fungal phosphate transporter genes are contemporaneously present in arbusculated cells. Mol. Plant Microbe. In. 20: 1055-1062.

Fiorilli, V., Vallino, M., Biselli, C., Faccio, A., Bagnaresi, P., and Bonfante, P. (2015).Host and non-host roots in rice: cellular and molecular approaches reveal differential responses to arbuscular mycorrhizal fungi. Front. Plant Sci. **6**:636.

Güimil, S., Chang, H.S., Zhu, T., Sesma, A., Osbourn, A., Roux, C., Ioannidis, V., Oakeley, E.J., Docquier, M., Descombes, P. et al. (2005). Comparative transcriptomics of rice reveals an ancient pattern of response to microbial colonization. Proc. Natl Acad. Sci. USA **102**: 8066-8070.

Hammer, Ø., Harper, D.A.T., and Ryan, P.D. (2001).PAST: paleontological statistics software package for education and data analysis. Palaeontol.Electron. **4**:9.

Hewitt, E.J. (1966). Sand and water culture methods used in the study of plant nutrition. Farnham Royal, UK: Commonwealth Agricultural Bureaux.

Trouvelot, A., Kough, J. L. and Gianinazzi-Pearson, V. in Mycorrhizae. Physiology and Genetics (eds Gianinazzi-Pearson, V. & Gianinazzi, S.) 217–221 (INRA Press, Paris, 1986)

Jamil, M., Kanampiu, F. K., Karaya, H., Charnikhova, T., and Bouwmeester, H. J. (2012). Striga hermonthica parasitism in maize in response to N and P fertilisers. Field Crops Res. **134**:1-10

Jamil, M., Kountche, B.A., Haider, I., Wang, J.Y., Aldossary, F., Zarban, R.A., et al (2019). Methylation at the C-3'in D-ring of strigolactone analogs reduces biological activity in root parasitic plants and rice. Front. Plant Sci. **10**: 353.

Kountche, B, A., Jamil, M., Yonli, D., Nikiema, M.P., Blanco-Ania, D., Asami, T., Zwanenburg, B., Al-Babili, S. (2019). Suicidal germination as a control strategy for *Striga* hermonthica (Benth.) in smallholder farms of sub-Saharan Africa. Plants, People, Planet doi.org/10.1002/ppp3.32

Livak, K. J. and Schmittgen, T. D. (2001). Analysis of relative gene expression data using real-time quantitative PCR and the $2^{\Delta\Delta}$CT Method. Methods **25(4)**:402-408.

Wang, J.Y., Haider, I., Jamil, M., Fiorilli, V., Saito, Y. et al. (2019). The apocarotenoid metabolite zaxinone regulates growth and strigolactone biosynthesis in rice. Nat. Commun. **10**:810. https://doi.org/10.1038/s41467-019-08461-1
