# Supplementary material for: Efficient Mimics for Elucidating Zaxinone Biology and Promoting Agricultural Applications
Source: Mol Plant. 2020 Nov 2;13(11):1654–61. doi: 10.1016/j.molp.2020.08.009 (PMC7656291; doi:10.1016/j.molp.2020.08.009)
Supplement: Document S2. Synthesis of Enantiopure Zaxinone and Analogs. Supplemental References [file mmc2.pdf]

Efficient Mimics for Elucidating Zaxinone Biology and Promoting Agricultural Applications

Jian You Wang, Muhammad Jamil, Pei-Yu Lin, Tsuyoshi Ota, Valentina Fiorilli, Mara Novero, Randa A. Zarban, Boubacar A. Kountche, Ikuo Takahashi, Claudio Martínez, Luisa Lanfranco, Paola Bonfante, Angel R. de Lera, Tadao Asami and Salim Al-Babili

Supplemental Document 2

The synthesis of the (iso)zaxinone compounds **1-4** followed the general strategies used for the synthesis of retinoids and carotenoids as recently reviewed. (Alvarez et al., 2014)

Synthesis of isozaxinone 1 (4-dihydrotrisporin B). (Gessler et al., 2002)**.**

(*E*)-Iodoalkenylcyclohexenol **5** was protected as silyl ether **6** in 83% yield upon treatment with TBDMSCl and imidazole,(Fontán et al., 2011) and the dienyliodide was subjected to a Stille cross-coupling reaction with (*E*)-alkenylstannane **7** catalyzed by $Pd_2dba_3 \cdot CHCl_3$ and $AsPh_3$ in the presence of LiCl and using NMP as solvent to provide, in 77% yield, trienol **8**.(Ito et al., 1990) Oxidation of the allylic alcohol with MnO_2 and Na_2CO_3 as described for other analogs(Fontán et al., 2011) gave rise to trienal **9**. Treatment of trienal **9** with acetone in basic media afforded the aldol condensation product trienylketone **10** in 65% yield,(Tanumihardjo, 2001) which was deprotected to provide the secondary alcohol **1** (isoxaxinone, 4-dihydrotrisporin B) (Gessler et al., 2002) upon treatment with HF · py. Having a hydroxyl group at the allylic position contributed to the instability of this compound, which requires careful handling.

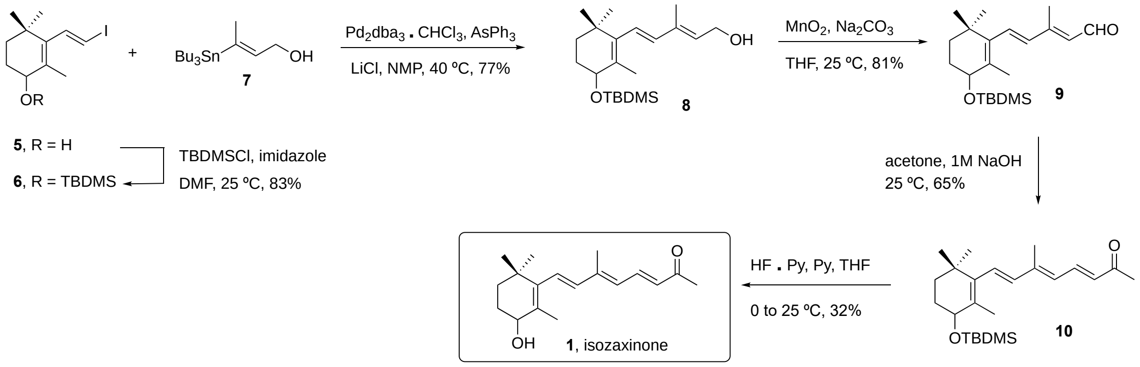

Scheme 2. Total synthesis of racemic isozaxinone.

Synthesis of isozaxinone 1 and analogues:

Enantiopure cyclohexenyltriflate **11** was prepared from TBS-protected enantiopure actinol,(Fontán et al., 2011; Ito et al., 1990; Yamano and Ito, 1993; Yamano et al., 1995) and subjected to Stille cross-coupling reaction with (2*Z*,4*E*)-stannanedienol **12** to afford trienol **13**. Allylic oxidation using MnO_2 and Na_2CO_3 took place uneventfully and provided trienal **14** in quantitative yield.(Domínguez et al., 2006) Aldol condensation with acetone as described above (Tanumihardjo, 2001) led in 79% yield the corresponding tetraenone **15**, which proved to be highly unstable and partly isomerized to the all-*trans* isomer. In fact, upon deprotection with TBAF at room temperature, a mixture of **2** and **3** in a 1:1 ratio was obtained.

A more efficient route to the trans isomer present in zaxinol **4** was followed along the same steps, using instead the (*E*,*E*)-dienylstannane **16**, with the intermediacy of **17** and trienal **18** obtained in 96% yield from trienol **17** with MnO_2 and Na_2CO_3 (Fontán et al., 2011). Aldol condensation as for the Z isomer (Tanumihardjo, 2001) provided all-*E*-tetraenone **19**. Reduction with $NaBH_4$ in the presence of $CeCl_3 \cdot 7H_2O$ afforded tetraenol **20** in 60% yield, the deprotection of which (TBAF, THF, rt, 43%) gave rise to unstable tetraene-diol zaxinol **4**.

Synthesis of O-Methylzaxinone

Enantiopure (*R*)-2-iodo-5-methoxy-1,3,3-trimethylcyclohex-1-ene **21** was prepared from enantiopure actinol and subjected to Stille cross-coupling reaction with (2*E*,4*E*)-dienylstannane **18** to afford trienol **22**. Allylic oxidation using MnO_2 and Na_2CO_3 took place uneventfully and provided trienal **23** in 53% yield.(Domínguez et al., 2006) Aldol condensation with acetone as described above (Tanumihardjo, 2001) led in 74% yield to the corresponding *O*-methylzaxinone **24.**

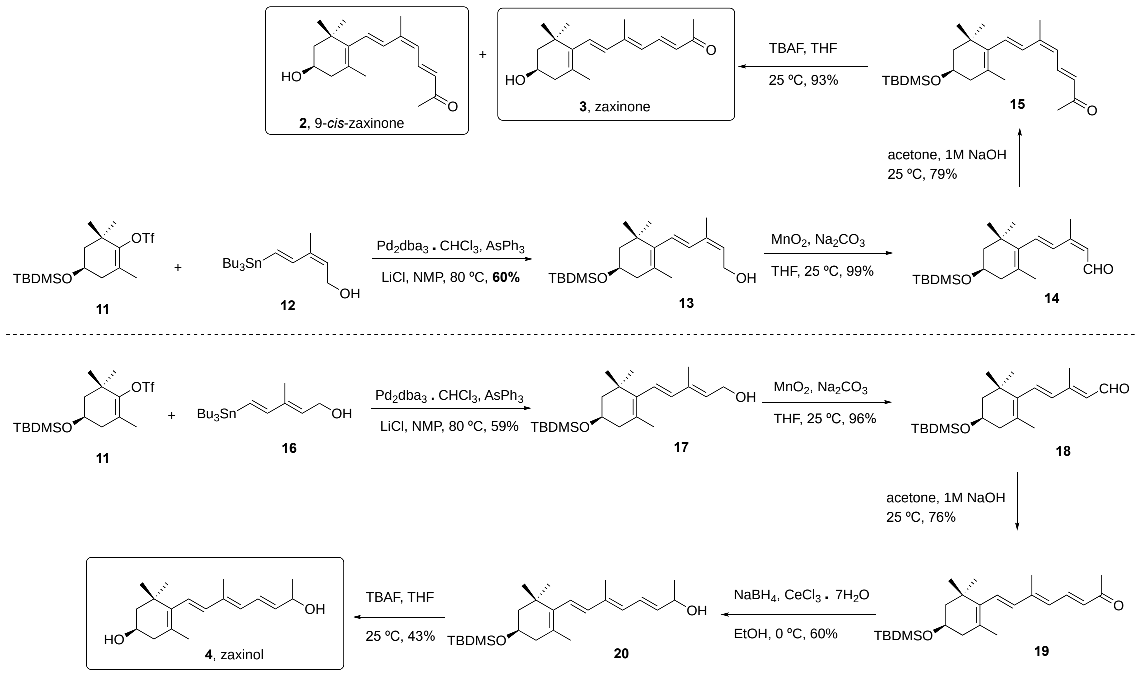

Scheme 2. Total synthesis of enantiopure zaxinone and analogues.

(*E*)-*tert*-Butyl((3-(2-iodovinyl)-2,4,4-trimethylcyclohex-2-en-1-yl)oxy)dimethylsilane 6.(Fontán et al., 2011)

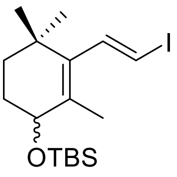

To a solution of (*E*)-3-(2-iodovinyl)-2,4,4-trimethylcyclohex-2-en-1-ol **5** (0.61 g, 2.10 mmol) in DMF (10 mL) were added imidazole (0.17 g, 2.52 mmol) and TBSCl (0.38 g, 2.52 mmol). The resulting mixture was stirred at 25 ºC for 12h. H_2O was added and the mixture was extracted with EtOAc (3x). The combined organic layers were dried over Na_2SO_4 and the solvent was evaporated. The residue was purified by column chromatography (silica gel, from 97:3 *n*-hexane/Et_3N to 98:2 *n*-hexane/EtOAc), to afford 0.66 g (77%) of a yellow oil identified as (*E*)-*tert*-butyl((3-(2-iodovinyl)-2,4,4-trimethylcyclohex-2-en-1-yl)oxy)dimethylsilane **6**. **^{1}H NMR** (400.13 MHz, C_6D_6): δ 6.92 (d, *J* = 14.9 Hz, 1H), 5.83 (d, *J* = 14.9 Hz, 1H), 3.80 (appt, *J* = 5.5 Hz, 1H), 1.67 (s, 3H), 1.63 – 1.51 (m, 3H), 1.23 – 1.17 (m, 1H), 0.98 (s, 9H), 0.85 (s, 3H), 0.80 (s, 3H), 0.07 (s, 3H), 0.06 (s, 3H) ppm. **^{13}C NMR** (100.62 MHz, C_6D_6): δ 143.9 (d), 141.4 (s),

132.9 (s), 79.8 (d), 71.1 (d), 35.2 (t), 34.3 (s), 29.5 (t), 28.3 (q), 28.0 (q), 26.1 (q, 3x), 18.7 (q), 18.3 (s), -4.0 (q), -4.5 (q) ppm.

(2*E*,4*E*)-5-(3-((*tert*-Butyldimethylsilyl)oxy)-2,6,6-trimethylcyclohex-1-en-1-yl)-3-methylpenta-2,4-dien-1-ol 8. (Haag and Eugster, 1982)

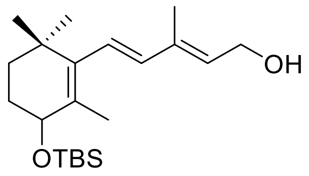

A solution of (*E*)-tert-butyl((3-(2-iodovinyl)-2,4,4-trimethylcyclohex-2-en-1-yl)oxy)dimethylsilane **6** (0.25 g, 0.61 mmol) in degassed NMP (5 mL) was added to a solution of $Pd_2(dba)_3 \cdot CHCl_3$ (31 mg, 0.03 mmol) and $AsPh_3$ (75 mg, 0.24 mmol) in NMP (1 mL). After stirring for 10 min, a solution of (*E*)-3-(tributylstannyl)but-2-en-1-ol **7** (285 mg, 0.79 mmol) in NMP (1 mL) and LiCl (78 mg, 1.83 mmol) were added and the mixture was stirred for 23h at 40 ºC. An aqueous solution of KF was added and the mixture was stirred for 10 min and then extracted with Et_2O (3x). The combined organic layers were washed with H_2O (3x), dried over Na_2SO_4 and the solvent was evaporated. The residue was purified by column chromatography (silica gel, from 97:3 *n*-hexane/Et_3N to 80:20 *n*-hexane/EtOAc), to afford 139 mg (65%) of a yellow oil identified as (2*E*,4*E*)-5-(3-((*tert*-butildimethylsilyl)oxy)-2,6,6-trimethylcyclohex-1-en-1-yl)-3-methylpenta-2,4-dien-1-ol **8**. **^{1}H NMR** (400.13 MHz, C_6D_6): δ 6.17 (d, *J* = 16.3 Hz, 1H), 6.11 (d, *J* = 16.3 Hz, 1H), 5.33 (t, *J* = 6.7 Hz, 1H), 4.06 - 3.94 (m, 3H), 1.95 (s, 3H), 1.83 – 1.68 (m, 3H), 1.62 (s, 3H), 1.44 – 1.31 (m, 1H), 1.07 (s, 3H), 1.04 (s, 3H), 1.02 (s, 9H), 0.13 (s, 3H), 0.12 (s, 3H) ppm. **^{13}C NMR** (100.62 MHz, C_6D_6): δ 140.6 (s), 138.7 (d), 135.5 (s), 131.1 (d), 128.2 (s), 126.2 (d), 71.5 (d), 59.4 (t), 35.5 (t), 34.9 (s), 29.8 (t), 28.8 (q), 28.4 (q), 26.2 (q, 3x), 18.9 (q), 18.4 (s), 12.4 (q), -3.9 (q), -4.4 (q) ppm.

(2*E*,4*E*)-5-(3-((*tert*-Butyldimethylsilyl)oxy)-2,6,6-trimethylcyclohex-1-en-1-yl)-3-methylpenta-2,4-dienal 9. (Katsuta et al., 1994)

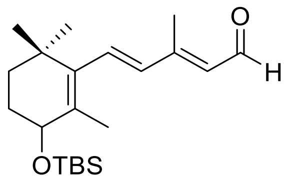

To a solution of MnO_2 (0.63 g, 7.2 mmol) and Na_2CO_3 (0.76 g, 7.2 mmol) in THF (6 mL) was added (2*E*,4*E*)-5-(3-((*tert*-butyldimethylsilyl)oxy)-2,6,6-trimethylcyclohex-1-en-1-yl)-3-methylpenta-2,4-dien-1-ol **8** (0.14 g, 0.40 mmol). The mixture was stirred for 1h at room temperature, and then filtered through Celite® and the solvent was evaporated. The mixture was purified by column chromatography (silica gel, from 97:3 *n*-hexane/Et_3N to 95:5 *n*-hexane/EtOAc), to afford 0.112 g (81%) of a yellow oil identified as (2*E*,4*E*)-5-(3-((*tert*-butyldimethylsilyl)oxy)-2,6,6-trimethylcyclohex-1-en-1-yl)-3-methylpenta-2,4-dienal **9**. **^{1}H NMR** (400.13 MHz, C_6D_6): δ 9.98 (d, *J* = 7.7 Hz, 1H), 6.38 (d, *J* = 16.0 Hz, 1H), 5.97 (d, *J* = 16.2 Hz, 1H), 5.86 (d, *J* = 7.7 Hz, 1H), 3.94 (appt, *J* = 5.2 Hz, 1H), 1.79 (s, 3H), 1.75 – 1.64 (m, 6H), 1.32 (ddd, *J* = 11.9, 7.9, 3.3 Hz, 1H), 1.01 (s, 9H), 0.96 (s, 3H), 0.92 (s, 3H), 0.12 (s, 3H), 0.11 (s, 3H) ppm. **^{13}C NMR** (100.62 MHz, C_6D_6): δ 190.1 (d), 152.7 (s), 139.7 (s), 137.4 (d), 134.2 (d), 133.3 (s), 129.9 (d), 71.3 (d), 35.5 (t), 34.9 (s), 29.6 (t), 28.7 (q), 28.3 (q), 26.4 (q, 3x), 18.8 (q), 18.4 (s), 12.4 (q), -3.9 (q), -4.5 (q) ppm.

(3*E*,5*E*,7*E*)-8-(3-((*tert*-Butyldimethylsilyl)oxy)-2,6,6-trimethylcyclohex-1-en-1-yl-6-methylocta-3,5,7-trien-2-one 10.

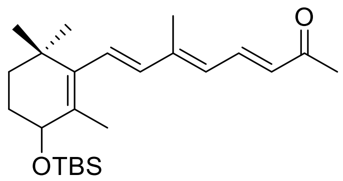

To a solution of (2*E*,4*E*)-5-(3-((*tert*-butyldimethylsilyl)oxy)-2,6,6-trimethylcyclohex-1-en-1-yl)-3-methylpenta-2,4-dienal **9** (0.11 g, 0.32 mmol) in acetone (1.6 mL) was added NaOH (0.028 mL, 1M in H_2O, 0.028 mmol) and the mixture was stirred for 12h at 25 ºC. Then, water was added and the mixture was extracted with Et_2O (3x). The combined organic layers were dried over Na_2SO_4 and the solvent was evaporated. The residue was purified by column chromatography (silica gel, from 97:3 *n*-hexane/Et_3N to 95:5 *n*-hexane/EtOAc), to afford 81 mg (65%) of a yellow oil identified as (3*E*,5*Z*,7*E*)-8-(3-((*tert*-butyldimethylsilyl)oxy)-2,6,6-trimethylcyclohex-1-en-1-yl)-6-methylocta-3,5,7-

trien-2-one **10**. ^{1}**H NMR** (400.13 MHz, C_6D_6): δ 7.52 (dd, *J* = 15.2, 11.8 Hz, 1H), 6.27 (d, *J* = 16.1 Hz, 1H), 6.17 (d, *J* = 16.1 Hz, 1H), 6.06 (d, *J* = 15.2 Hz, 1H), 5.95 (d, *J* = 11.8 Hz, 1H), 4.01 (appt, *J* = 5.3 Hz, 1H), 1.92 (s, 3H), 1.91 (s, 3H), 1.81 – 1.70 (m, 3H), 1.68 (s, 3H), 1.40 – 1.32 (m, 1H), 1.04 (s, 3H), 1.02 (s, 9H), 1.01 (s, 3H), 0.14 (s, 3H), 0.12 (s, 3H) ppm. ^{13}**C NMR** (100.62 MHz, C_6D_6): δ 196.2 (s), 144.1 (s), 140.3 (s), 138.3 (d), 138.0 (d), 132.3 (s), 130.3 (d), 130.2 (d), 129.1 (d), 71.5 (d), 35.6 (t), 34.9 (s), 29.7 (t), 28.8 (q), 28.4 (q), 27.7 (q), 26.2 (q, 3x), 19.1 (q), 18.4 (s), 12.8 (q), -3.9 (q), -4.5 (q) ppm. **HRMS (ESI$^+$)**: Calcd. for $C_{24}H_{40}NaO_2Si$ [$(M+Na)^+$], 411.2687, found 411.2690. **IR** (NaCl): υ 2955 (s, C=C), 2922 (s, C=C), 2856 (s, C=C), 1663 (s, C=O), 1580 (s), 1360 (w), 1252 (s), 1154 (w), 1005 (w) cm^{-1}.

(3*E*,5*E*,7*E*)-8-(3-Hydroxy-2,6,6-trimethylcyclohex-1-en-1-yl)-6-methylocta-3,5,7-trien-2-one 1 (4-dihydrotrisporin B). (Gessler et al., 2002)

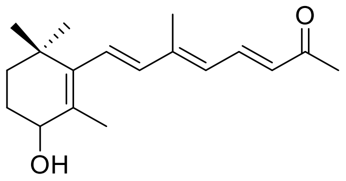

To a solution of (3*E*,5*Z*,7*E*)-8-(3-((*tert*-butyldimethylsilyl)oxy)-2,6,6-trimethylcyclohex-1-en-1-yl)-6-methylocta-3,5,7-trien-2-one **10** (46 mg, 0.12 mmol) in THF (8.4 mL), pyridine (7.0 mL, 865.5 mmol) was added. Then, the mixture was cooled down to 0 ºC and HF·Py (6.4 mL) was added, then was stirred at 25 ºC for 24h. EtOAc and phosphate buffer were added and the mixture was extracted with EtOAc (3x). The combined organic layers were dried over Na_2SO_4 and the solvent was evaporated. The residue was purified by column chromatography (silica gel, from 97:3 *n*-hexane/Et_3N to 60:40 *n*-hexane/EtOAc), to afford 10 mg (32%) of a yellow oil identified as (3*E*,5*E*,7*E*)-8-(3-hydroxy-2,6,6-trimethylcyclohex-1-en-1-yl)-6-methylocta-3,5,7-trien-2-one **1**. ^{1}**H NMR** (400.13 MHz, C_6D_6): δ 7.53 (dd, *J* = 15.2, 11.8 Hz, 1H), 6.24 (d, *J* = 16.1 Hz, 1H), 6.15 (d, *J* = 16.1 Hz, 1H), 6.05 (d, *J* = 15.1 Hz, 1H), 5.98 (d, *J* = 11.8 Hz, 1H), 3.80 (appt, *J* = 5.2 Hz, 1H), 1.92 (s, 3H), 1.85 (s, 3H), 1.68 (s, 3H), 1.52 – 1.40 (m, 4H), 1.01 (s, 3H), 0.96 (s, 3H) ppm.

(2*Z*,4*E*)-5-((*R*)-4-((*tert*-Butyldimethylsilyl)oxy)-2,6,6-trimethylcyclohex-1-en-1-yl)-3-methylpenta-2,4-dien-1-ol 13. (Ito et al., 1988)

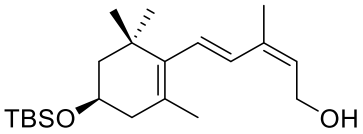

Following the general procedure for the Stille reaction described above, the reaction of (*R*)-4-((*tert*-butyldimethylsilyl)oxy)-2,6,6-trimethylcyclohex-1-en-1-yl trifluoromethanesulfonate **11** (200 mg, 0.50 mmol), $Pd_2(dba)_3 \cdot CHCl_3$ (23 mg, 0.025 mmol), $AsPh_3$ (61 mg, 0.20 mmol), LiCl (63 mg, 1.5 mmol) and (2*Z*,4*E*)-3-methyl-5-(tributylstannyl)penta-2,4-dien-1-ol **12** (230 mg, 0.60 mmol) in NMP (6.5 mL) afford, after purification by column chromatography (silica gel, from 97:3 *n*-hexane/Et_3N to 80:20 *n*-hexane/EtOAc), 105 mg (60%) of a yellow oil identified as (2*Z*,4*E*)-5-((*R*)-4-((*tert*-butyldimethylsilyl)oxy)-2,6,6-trimethylcyclohex-1-en-1-yl)-3-methylpenta-2,4-dien-1-ol **13**. **^{1}H NMR** (400.13 MHz, C_6D_6): δ 6.52 (d, *J* = 16.1 Hz, 1H), 6.17 (d, *J* = 16.1 Hz, 1H), 5.46 (t, *J* = 6.9 Hz, 1H), 4.10 (d, *J* = 6.9 Hz, 2H), 4.08 – 4.04 (m, 1H), 2.36 – 2.18 (m, 2H), 1.80 (s, 3H), 1.78 – 1.69 (m, 1H), 1.71 (s, 3H), 1.70 – 1.69 (m, 1H), 1.10 (s, 3H), 1.06 (s, 3H), 1.04 (s, 9H), 0.15 (s, 3H), 0.14 (s, 3H) ppm. **^{13}C NMR** (100.62 MHz, C_6D_6): δ 137.8 (s), 134.5 (s), 130.9 (d), 129.3 (d), 128.2 (d), 127.1 (s), 66.0 (d), 58.5 (t), 49.3 (t), 43.5 (t), 37.1 (s), 30.4 (q), 28.8 (q), 26.2 (q, 3x), 21.7 (d), 20.4 (d), 18.4 (s), -4.3 (q, 2x) ppm.

(2*Z*,4*E*)-5-((*R*)-4-((*tert*-Butyldimethylsilyl)oxy)-2,6,6-trimethylcyclohex-1-en-1-yl)-3-methylpenta-2,4-dienal 14.

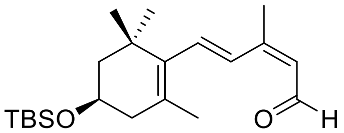

Following the general procedure for the alcohol oxidation, the reaction of (2*Z*,4*E*)-5-((*R*)-4-((*tert*-butyldimethylsilyl)oxy)-2,6,6-trimethylcyclohex-1-en-1-yl)-3-methylpenta-2,4-dien-1-ol **13** (50 mg, 0.14 mmol), MnO_2 (120 mg, 1.4 mmol) and Na_2CO_3 (150 mg, 1.4 mmol) in THF (1.5 mL) afford, after purification by column chromatography (silica gel, from 97:3 *n*-hexane/Et_3N to 95:5 *n*-hexane/EtOAc), 49 mg (99%) of a yellow oil identified as (2*Z*,4*E*)-5-((*R*)-4-((*tert*-butyldimethylsilyl)oxy)-2,6,6-trimethylcyclohex-1-en-1-yl)-3-methylpenta-2,4-dienal **14**. $[\alpha]_D^{24}$ -50.0 (*c* 0.43, MeOH). **^{1}H NMR** (400.13 MHz, C_6D_6): δ 10.13 (d, *J* = 7.4 Hz, 1H), 7.02 (d, *J* = 16.1 Hz, 1H), 6.31 (d, *J* = 16.1

Hz, 1H), 5.78 (d, *J* = 7.3 Hz, 1H), 4.03 (dddd, *J* = 11.4, 9.6, 5.8, 3.7 Hz, 1H), 2.33 – 2.09 (m, 2H), 1.75 (ddd, *J* = 12.4, 3.8, 1.9 Hz, 1H), 1.65 – 1.60 (m, 1H), 1.59 (s, 3H), 1.56 (s, 3H), 1.04 (s, 9H), 1.02 (s, 3H), 0.97 (s, 3H), 0.15 (s, 3H), 0.14 (s, 3H) ppm. **^{13}C NMR** (100.62 MHz, C_6D_6): δ 188.8 (d), 152.8 (s), 136.9 (s), 135.0 (d), 129.7 (s), 129.2 (d), 128.6 (d), 65.7 (d), 49.2 (t), 43.6 (t), 37.0 (s), 30.2 (q), 28.7 (q), 26.2 (q, 3x), 21.7 (q), 20.6 (q), 18.4 (s), -4.3 (q, 2x) ppm. **HRMS (ESI$^+$)**: Calcd. for $C_{21}H_{37}O_2Si$ [$(M+H)^+$], 349.2554, found 349.2557. **IR** (NaCl): υ 2955 (s, C=C), 2926 (s, C=C), 2855 (s, C=C), 1669 (s, C=O), 1613 (w), 1361 (w), 1255 (w), 1115 (w), 1084 (s), 836 (w) cm^{-1}.

(3*E*,5*Z*,7*E*)-8-((*R*)-4-((*tert*-Butyldimethylsilyl)oxy)-2,6,6-trimethylcyclohex-1-en-1-yl)-6-methylocta-3,5,7-trien-2-one 15.

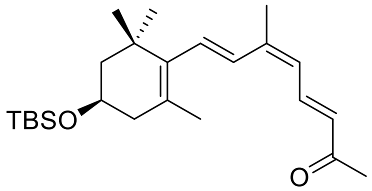

Following the general procedure for the aldol reaction, the reaction of (2*Z*,4*E*)-5-((*R*)-4-((*tert*-butyldimethylsilyl)oxy)-2,6,6-trimethylcyclohex-1-en-1-yl)-3-methylpenta-2,4-dienal **14** (48 mg, 0.14 mmol) with NaOH (0.013 mL, 0.013 mmol, 1M) in acetone (0.70 mL) afford, after purification by column chromatography (silica gel, from 97:3 *n*-hexane/Et_3N to 95:5 *n*-hexane/EtOAc), 43 mg (79%) of a yellow oil identified as (3*E*,5*Z*,7*E*)-8-((*R*)-4-((*tert*-butyldimethylsilyl)oxy)-2,6,6-trimethylcyclohex-1-en-1-yl)-6-methylocta-3,5,7-trien-2-one **15**. **$[\alpha]_D^{24}$** -90.7 (*c* 0.26, MeOH). **^{1}H NMR** (400.13 MHz, C_6D_6): δ 7.75 (dd, *J* = 15.2, 11.8 Hz, 1H), 6.84 (d, *J* = 16.0 Hz, 1H), 6.29 (d, *J* = 16.0 Hz, 1H), 6.00 (d, *J* = 15.1 Hz, 1H), 5.87 (d, *J* = 11.8 Hz, 1H), 4.14 – 3.96 (m, 1H), 2.35 – 2.12 (m, 2H), 1.86 (s, 3H), 1.79 (s, 3H), 1.75 (dd, *J* = 3.7, 1.8 Hz, 1H), 1.69 – 1.67 (m, 1H), 1.65 (s, 3H), 1.06 (s, 3H), 1.04 (s, 9H), 1.03 (s, 3H), 0.14 (s, 3H), 0.13 (s, 3H) ppm. **^{13}C NMR** (100.62 MHz, C_6D_6): δ 196.3 (s), 142.9 (s), 137.5 (s), 136.6 (d), 131.3 (d), 130.1 (d), 129.8 (d), 128.6 (s), 127.4 (d), 65.8 (d), 49.2 (t), 43.5 (t), 37.1 (s), 30.3 (q), 28.8 (q), 27.6 (q), 26.2 (q, 3x), 21.7 (q), 20.9 (q), 18.4 (s), -4.3 (q, 2x) ppm. **HRMS (ESI$^+$)**: Calcd. for $C_{24}H_{41}O_2Si$ [$(M+H)^+$], 389.2868, found 389.2870. **IR** (NaCl): υ 2955 (s, C=C), 2927 (s, C=C), 2856 (s, C=C), 1661 (s, C=O), 1588 (s), 1462 (w), 1361 (w), 1252 (s), 1083 (s), 973 (w) cm^{-1}.

(3*E*,5*Z*,7*E*)-8-((*R*)-4-Hydroxy-2,6,6-trimethylcyclohex-1-en-1-yl)-6-methylocta-3,5,7-trien-2-one 2 and (3*E*,5*E*,7*E*)-8-((*R*)-4-Hydroxy-2,6,6-trimethylcyclohex-1-en-1-yl)-6-methylocta-3,5,7-trien-2-one 3. (Maoka et al., 2001)

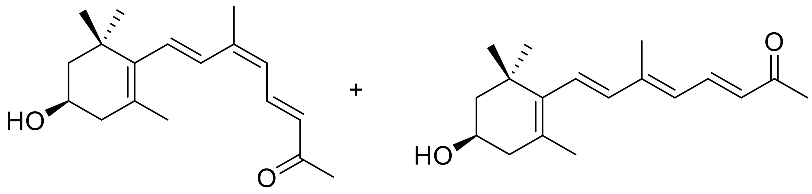

To a cooled (0 ºC) solution of (3*E*,5*Z*,7*E*)-8-((*R*)-4-((*tert*-butyldimethylsilyl)oxy)-2,6,6-trimethylcyclohex-1-en-1-yl)-6-methylocta-3,5,7-trien-2-one **15** (43 mg, 0.11 mmol) in THF (1.0 mL), TBAF (0.17 mL, 1M in THF, 0.17 mmol) was added. The mixture was stirred for 3.5h at 25 ºC. Then, Et_2O was added and washed with an aqueous solution of $NaHCO_3$. The aqueous layer was extracted with Et_2O (3x) and the combined organic layers were washed with brine, dried over Na_2SO_4 and the solvent was evaporated. The residue was purified by column chromatography (silica gel, from 97:3 *n*-hexane/Et_3N to 40:60 *n*-hexane/EtOAc), to afford 14 mg (47%) of a yellow oil identified as (3*E*,5*Z*,7*E*)-8-((*R*)-4-hydroxy-2,6,6-trimethylcyclohex-1-en-1-yl)-6-methylocta-3,5,7-trien-2-one **2** and 14 mg (47%) of a yellow oil identified as (3*E*,5*E*,7*E*)-8-((*R*)-4-hydroxy-2,6,6-trimethylcyclohex-1-en-1-yl)-6-methylocta-3,5,7-trien-2-one **3**.

Data for **(3*E*,5*Z*,7*E*)-8-((*R*)-4-Hydroxy-2,6,6-trimethylcyclohex-1-en-1-yl)-6-methylocta-3,5,7-trien-2-one 2.** $[\alpha]_D^{26}$ -16.9 (*c* 0.09, MeOH). **^{1}H NMR** (400.13 MHz, C_6D_6): δ 7.75 (dd, *J* = 15.1, 11.9 Hz, 1H), 6.82 (d, *J* = 16.0 Hz, 1H), 6.27 (d, *J* = 16.0 Hz, 1H), 5.99 (d, *J* = 15.1 Hz, 1H), 5.86 (d, *J* = 11.9 Hz, 1H), 3.80 – 3.73 (m, 1H), 2.20 – 2.14 (m, 1H), 1.96 - 1.89 (m, 1H), 1.86 (s, 3H), 1.77 (s, 3H), 1.62 (s, 3H), 1.60 – 1.50 (m, 1H), 1.43 – 1.37 (m, 1H), 1.01 (s, 3H), 0.99 (s, 3H) ppm. **^{13}C NMR** (100.62 MHz, C_6D_6): δ 196.4 (s), 143.1 (s), 137.9 (s), 137.4 (s), 136.6 (d), 131.3 (d), 130.1 (d), 129.7 (d), 127.4 (d), 64.5 (d), 48.7 (t), 42.8 (t), 37.0 (s), 30.4 (q), 28.8 (q), 27.7 (q), 21.7 (q), 20.9 (q) ppm.

Data for **(3*E*,5*E*,7*E*)-8-((*R*)-4-Hydroxy-2,6,6-trimethylcyclohex-1-en-1-yl)-6-methylocta-3,5,7-trien-2-one 3**. $[\alpha]_D^{25}$ +5.2 (*c* 0.22, MeOH). **^{1}H NMR** (400.13 MHz,

C_6D_6): δ 7.54 (dd, *J* = 15.2, 11.7 Hz, 1H), 6.23 (d, *J* = 16.0 Hz, 1H), 6.12 (d, *J* = 16.1 Hz, 1H), 6.05 (d, *J* = 15.2 Hz, 1H), 5.99 (d, *J* = 11.5 Hz, 1H), 3.81 – 3.73 (m, 1H), 2.24 – 2.17 (m, 1H), 1.99 – 1.93 (m, 1H), 1.92 (s, 3H), 1.68 (s, 3H), 1.64 (s, 3H), 1.62 – 1.58 (m, 2H), 1.02 (s, 6H) ppm. **^{13}C NMR** (100.62 MHz, C_6D_6): δ 196.2 (s), 144.2 (s), 138.0 (d), 137.9 (d), 137.5 (s), 130.2 (d), 129.9 (d), 128.9 (d), 128.7 (s), 64.5 (d), 48.8 (t), 42.9 (t), 37.1 (s), 30.4 (q), 28.8 (q), 27.8 (q), 21.7 (q), 12.7 (q) ppm.

(2*E*,4*E*)-5-(1*R*)-4-((*tert*-Butyldimethylsilyl)oxy-2,6,6-trimethylcyclohex-1-en-1-yl)-3-methylpenta-2,4-dien-1-ol 17. (Domínguez et al., 2006)

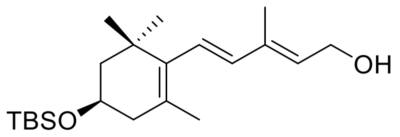

Following the general procedure for the Stille reaction, the reaction of (*R*)-4-((*tert*-butyldimethylsilyl)oxy)-2,6,6-trimethylcyclohex-1-en-1-yl trifluoromethanesulfonate **11** (0.40 g, 1.0 mmol), (2*E*,4*E*)-3-methyl-5-(tributylstannyl)penta-2,4-dien-1-ol **16** (0.46 g, 1.2 mmol), $Pd_2(dba)_3 \cdot CHCl_3$ (0.005 g, 0.05 mmol), $AsPh_3$ (0.12 g, 0.4 mmol) and LiCl (0.13 g, 3.0 mmol) in NMP (13 mL) afford, after purification by column chromatography (silica gel, from 97:3 *n*-hexane/Et_3N to *n*-hexane/EtOAc 80:20), 0.206 g (59%) of a yellow oil identified as (2*E*,4*E*)-5-(1*R*)-4-((*tert*-butyldimethylsilyl)oxy-2,6,6-trimethylcyclohex-1-en-1-yl)-3-methylpenta-2,4-dien-1-ol **17**. **^{1}H NMR** (400.13 MHz, C_6D_6): δ 6.16 – 6.07 (m, 2H), 5.60 – 5.52 (m, 1H), 4.09 (dddd, *J* = 11.6, 9.5, 5.8, 3.7 Hz, 1H), 4.02 (d, *J* = 6.7 Hz, 2H), 2.42 – 2.19 (m, 2H), 1.80 (ddd, *J* = 12.3, 3.7, 1.9 Hz, 1H), 1.72 (s, 3H), 1.69 – 1.65 (m, 1H), 1.63 (s, 3H), 1.11 (s, 3H), 1.08 (s, 3H), 1.05 (s, 9H), 0.15 (s, 3H), 0.14 (s, 3H) ppm. **^{13}C NMR** (100.62 MHz, C_6D_6): δ 138.5 (d), 137.6 (s), 135.6 (s), 130.8 (d), 126.7 (s), 125.9 (d), 66.1 (d), 59.4 (t), 49.3 (t), 43.4 (t), 37.2 (s), 30.4 (q), 28.8 (q), 26.2 (q, 3x), 21.7 (q), 18.4 (s), 12.4 (q), -4.3 (q, 2x) ppm.

(2*E*,4*E*)-5-((*R*)-4-((*tert*-Butyldimethylsilyl)oxy)-2,6,6-trimethylcyclohex-1-en-1-yl)-3-methylpenta-2,4-dienal 18. (Domínguez et al., 2006)

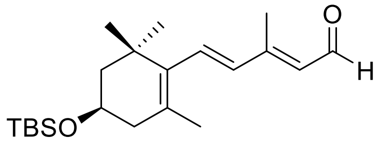

Following the general procedure for the alcohol oxidation, the reaction of (2*E*,4*E*)-5-(1*R*)-4-((*tert*-butyldimethylsilyl)oxy-2,6,6-trimethylcyclohex-1-en-1-yl)-3-methylpenta-2,4-dien-1-ol **17** (0.055 g, 0.16 mmol), MnO_2 (0.14 g, 1.6 mmol) and Na_2CO_3 (0.17 g, 1.6 mmol) in THF (1.5 mL) afford, after purification by column chromatography (silica gel, from 97:3 *n*-hexane/Et_3N to 95:5 *n*-hexane/EtOAc), 0.053 g (96%) of a yellow oil identified as (2*E*,4*E*)-5-((*R*)-4-((*tert*-butyldimethylsilyl)oxy)-2,6,6-trimethylcyclohex-1-en-1-yl)-3-methylpenta-2,4-dienal **18**. **^{1}H NMR** (400.13 MHz, C_6D_6): δ 10.0 (d, *J* = 7.7 Hz, 1H), 6.38 (d, *J* = 16.1 Hz, 1H), 5.96 (d, *J* = 16.1 Hz, 1H), 5.91 (d, *J* = 7.8 Hz, 1H), 4.01 (dddd, *J* = 12.6, 9.3, 5.8, 3.6 Hz, 1H), 2.27 (dd, *J* = 17.1, 5.6 Hz, 1H), 2.15 (dd, *J* = 17.4, 9.2 Hz, 1H), 1.77 -1.74 (m, 1H), 1.72 (s, 3H), 1.63 – 1.56 (m, 1H), 1.54 (s, 3H), 1.04 (s, 9H), 1.0 (s, 3H), 0.97 (s, 3H), 0.14 (s, 3H), 0.13 (s, 3H) ppm. **^{13}C NMR** (100.62 MHz, C_6D_6): δ 189.9 (d), 152.8 (s), 137.0 (d), 136.9 (s), 133.8 (d), 129.7 (d), 129.5 (s), 65.7 (d), 49.2 (t), 43.5 (t), 37.1 (s), 30.2 (q), 28.7 (q), 26.2 (q, 3x), 21.6 (q), 18.4 (s), 12.4 (q), -4.3 (q, 2x) ppm.

(3*E*,5*E*,7*E*)-8-((*R*)-4-((*tert*-Butyldimethylsilyl)oxy)-2,6,6-trimethylcyclohex-1-en-1-yl)-6-methylocta-3,5,7-trien-2-one 19.

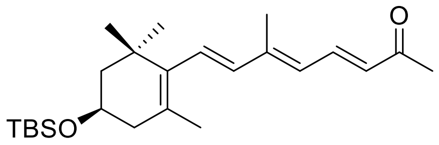

Following the general procedure for the aldol reaction, the reaction of 2*E*,4*E*)-5-((*R*)-4-((tert-butyldimethylsilyl)oxy)-2,6,6-trimethylcyclohex-1-en-1-yl)-3-methylpenta-2,4-dienal **18** (0.17 g, 0.50 mmol), NaOH (0.045 mL, 1M in H_2O, 0.045 mmol) in acetone (2.5 mL) afford, after purification by column chromatography (silica gel, from 97:3 *n*-hexane/Et_3N to 95:5 *n*-hexane/EtOAc), 0.15 g (76%) of a yellow oil identified as (3*E*,5*E*,7*E*)-8-((*R*)-4-((*tert*-butyldimethylsilyl)oxy)-2,6,6-trimethylcyclohex-1-en-1-yl)-6-methylocta-3,5,7-trien-2-one **19**. $[\alpha]_D^{25}$ -80.1 (*c* 0.44, MeOH). **^{1}H NMR** (400.13 MHz, C_6D_6): δ 7.54 (dd, *J* = 15.2, 11.7 Hz, 1H), 6.25 (d, *J* = 16.2 Hz, 1H), 6.14 (d, *J* = 16.2 Hz, 1H), 6.05 (d, *J* = 15.2 Hz, 1H), 5.99 (d, *J* = 11.7 Hz, 1H), 4.16 – 4.0 (m, 1H),

2.33 (dd, J = 17.3, 5.8 Hz, 1H), 2.28 – 2.16 (m, 1H), 1.92 (s, 3H), 1.82 – 1.75 (m, 1H), 1.68 (s, 3H), 1.67 (s, 3H), 1.43 – 1.33 (m, 1H), 1.08 (s, 3H), 1.04 (s, 12H, Si-$(CH_3)_3$ + CH_3), 0.15 (s, 3H), 0.14 (s, 3H) ppm. **^{13}C NMR** (100.62 MHz, C_6D_6): δ 196.3 (s), 144.3 (s), 138.0 (d), 137.9 (d), 137.5 (s), 130.2 (d), 129.9 (d), 128.9 (d), 127.9 (s), 65.9 (d), 49.3 (t), 43.6 (t), 37.2 (s), 30.4 (q), 28.8 (q), 27.8 (q), 26.2 (q, 3x), 21.8 (q), 18.4 (s), 12.5 (q), -4.1 (q), -4.2 (q) ppm. **HRMS (ESI$^+$)**: Calcd. for $C_{24}H_{41}O_2Si$ [$(M+H)^+$], 389.2868, found 389.2870. **IR** (NaCl): υ 2955 (s, C=C), 2927 (s, C=C), 2856 (s, C=C), 1661 (s, C=O), 1589 (s), 1361 (w), 1251 (s), 1083 (s), 974 (w), 836 (w) cm^{-1}.

(3*E*,5*E*,7*E*)-8-((*R*)-4-((*tert*-Butyldimethylsilyl)oxy)-2,6,6-trimethylcyclohex-1-en-1-yl)-6-methylocta-3,5,7-trien-2-ol 20.

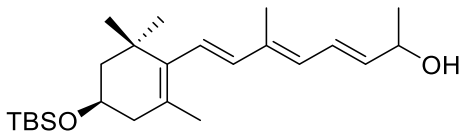

To a cooled (0 ºC) solution of (3*E*,5*E*,7*E*)-8-((*R*)-4-((*tert*-butyldimethylsilyl)oxy)-2,6,6-trimethylcyclohex-1-en-1-yl)-6-methylocta-3,5,7-trien-2-one **19** (0.05 g, 0.13 mmol) and $CeCl_3 \cdot 7H_2O$ (0.06 g, 0.16 mmol) in ethanol (1.30 mL), $NaBH_4$ (0.005 g, 0.13 mmol) was added. The mixture was stirred at 0 ºC for 1h. Then, a saturated solution of NH_4Cl was added and the mixture was extracted with Et_2O (3x). The combined organic layers were dried over Na_2SO_4, filtered and concentrated under reduced pressure. The residue was purified by column chromatography (silica gel, from 97:3 *n*-hexane/Et_3N to 80:20 *n*-hexane/EtOAc), to afford 0.030 g (60%) of a yellow oil identified as (3*E*,5*E*,7*E*)-8-((*R*)-4-((*tert*-butyldimethylsilyl)oxy)-2,6,6-trimethylcyclohex-1-en-1-yl)-6-methylocta-3,5,7-trien-2-ol **20**. $[\alpha]_D^{25}$ -33.7 (*c* 0.48, MeOH). **^{1}H NMR** (400.13 MHz, C_6D_6): δ 6.57 (dd, J = 15.2, 11.3 Hz, 1H), 6.25 (d, J = 16.1 Hz, 1H), 6.17 (d, J = 16.1 Hz, 1H), 6.10 (d, J = 11.3 Hz, 1H), 5.64 (dd, J = 15.0, 6.1 Hz, 1H), 4.17 – 4.05 (m, 2H), 2.39 – 2.32 (m, 2H), 1.83 (s, 3H), 1.80 – 1.78 (m, 1H), 1.75 (s, 3H), 1.72 – 1.65 (m, 1H), 1.14 – 1.13 (m, 6H), 1.10 (s, 3H), 1.09 (s, 9H), 0.15 (s, 3H), 0.14 (s, 3H) ppm. **^{13}C NMR** (100.62 MHz, C_6D_6): δ 138.9 (d), 138.6 (d), 137.9 (s), 135.4 (s), 130.4 (d), 126.9 (s), 126.1 (d), 125.7 (d), 68.5 (d), 66.1 (d), 49.4 (t), 43.6 (t), 37.3 (s), 30.4 (q), 28.8 (q), 26.2 (q, 3x), 23.8 (q), 21.8 (q), 18.4 (s), 12.6 (q), -4.3 (q, 2x) ppm. **IR** (NaCl): υ 3600-3000 (s, OH), 2955 (s, C=C), 2928 (s, C=C), 2856 (s, C=C), 1462 (w), 1254 (w), 1083 (w) cm^{-1}.

(*R*)-4-((1*E*,3*E*,5*E*)-7-Hydroxy-3-methylocta-1,3,5-trien-1-yl)-3,5,5-trimethylcyclohex-3-en-1-ol 4.

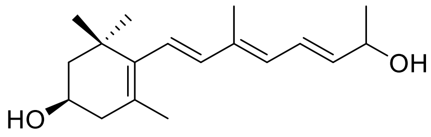

Following the general procedure for alcohol deprotection, the reaction of (3*E*,5*E*,7*E*)-8-((*R*)-4-((*tert*-butyldimethylsilyl)oxy)-2,6,6-trimethylcyclohex-1-en-1-yl)-6-methylocta-3,5,7-trien-2-ol **20** (0.030 g, 0.077 mmol) and TBAF (0.35 mL, 1M in THF, 0.35 mmol) in THF (1.0 mL) afford, after purification by column chromatography (silica gel, from 97:3 *n*-hexane/Et_3N to 60:40 *n*-hexane/EtOAc), 0.009 g (43%) of a yellow oil identified as (*R*)-4-((1*E*,3*E*,5*E*)-7-hydroxy-3-methylocta-1,3,5-trien-1-yl)-3,5,5-trimethylcyclohex-3-en-1-ol **4**. $[\alpha]_D^{25}$ -61.5 (*c* 0.1, MeOH). **^{1}H NMR** (400.13 MHz, C_6D_6): δ 6.56 (dd, *J* = 15.0, 11.2 Hz, 1H), 6.22 (d, *J* = 16.1 Hz, 1H), 6.15 (d, *J* = 16.1 Hz, 1H), 6.09 (d, *J* = 11.2 Hz, 1H), 5.64 (dd, *J* = 15.1, 6.2 Hz, 1H), 4.13 (t, *J* = 6.5 Hz, 1H), 3.89 – 3.76 (m, 1H), 2.23 (dd, *J* = 17.0, 5.8 Hz, 1H), 1.98 (dd, *J* = 17.0, 9.7 Hz, 1H), 1.82 (s, 3H), 1.71 (s, 3H), 1.69 – 1.58 (m, 1H), 1.49 – 1.36 (m, 1H), 1.14 (d, *J* = 6.4 Hz, 3H), 1.07 (s, 3H), 1.06 (s, 3H) ppm. **^{13}C NMR** (100.62 MHz, C_6D_6): δ 138.9 (d), 138.6 (d), 137.9 (s), 135.4 (s), 130.4 (d), 126.6 (s), 126.1 (d), 125.7 (d), 68.5 (d), 64.7 (d), 48.9 (t), 42.9 (t), 37.2 (s), 30.4 (q), 28.8 (q), 23.8 (q), 21.7 (q), 12.6 (q) ppm. **HRMS (ESI$^+$)**: Calcd. for $C_{18}H_{28}NaO_2$ $[(M+Na)^+]$, 299.1982, found 299.1982. **IR** (NaCl): υ 3600-3000 (s, OH), 2962 (s, C=C), 2923 (s, C=C), 1451 (w), 1362 (w), 1140 (w), 969 (w) cm^{-1}.

(2*E*,4*E*)-5-((*R*)-4-Methoxy-2,6,6-trimethylcyclohex-1-en-1-yl)-3-methylpenta-2,4-dien-1-ol 22.

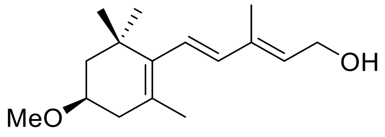

Following the general procedure for the Stille reaction, the reaction of (*R*)-2-iodo-5-methoxy-1,3,3-trimethylcyclohex-1-ene **21** (0.20 g, 0.71 mmol), (2*E*,4*E*)-3-methyl-5-(tributylstannyl)penta-2,4-dien-1-ol **16** (0.33 g, 0.86 mmol), $Pd_2(dba)_3 \cdot CHCl_3$ (0.037 g, 0.04 mmol), $AsPh_3$ (0.087 g, 0.28 mmol) and LiCl (0.12 g, 2.84 mmol) in NMP (9 mL)

afforded, after purification by column chromatography (silica gel, from 97:3 *n*-hexane/Et_3N to 80:20 *n*-hexane/EtOAc), 0.058 g (33%) of a yellow oil identified as (2*E*,4*E*)-5-((*R*)-4-methoxy-2,6,6-trimethylcyclohex-1-en-1-yl)-3-methylpenta-2,4-dien-1-ol **22**. **^{1}H NMR** (400.13 MHz, C_6D_6): δ 6.16 – 6.07 (m, 2H), 5.57 (t, *J* = 6.4 Hz, 1H), 4.03 (d, *J* = 6.7 Hz, 2H), 3.48 (dddd, *J* = 11.5, 9.2, 5.7, 3.5 Hz, 1H), 3.23 (s, 3H), 2.41 – 2.32 (m, 2H), 2.16 – 2.06 (m, 1H), 1.90 (ddd, *J* = 12.3, 3.5, 1.9 Hz, 1H), 1.71 (s, 3H), 1.63 (s, 3H), 1.08 (6, 6H) ppm.

(2*E*,4*E*)-5-((*R*)-4-Methoxy-2,6,6-trimethylcyclohex-1-en-1-yl)-3-methylpenta-2,4-dienal 23.

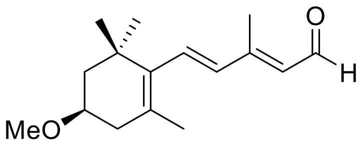

Following the general procedure for the oxidation of allylic alcohols, the reaction of (2*E*,4*E*)-5-((*R*)-4-methoxy-2,6,6-trimethylcyclohex-1-en-1-yl)-3-methylpenta-2,4-dien-1-ol **22** (0.058 g, 0.23 mmol), MnO_2 (0.36 g, 4.17 mmol) and Na_2CO_3 (0.44 g, 4.17 mmol) in THF (3.5 mL) afforded, after purification by column chromatography (silica gel, from 97:3 *n*-hexane/Et_3N to 95:5 *n*-hexane/EtOAc), 0.030 g (53%) of a yellow oil, which was identified as (2*E*,4*E*)-5-((*R*)-4-methoxy-2,6,6-trimethylcyclohex-1-en-1-yl)-3-methylpenta-2,4-dienal **23**. **^{1}H NMR** (400.13 MHz, C_6D_6): δ 10.0 (d, *J* = 7.7 Hz, 1H), 6.38 (d, *J* = 16.2 Hz, 1H), 5.96 (d, *J* = 16.2 Hz, 1H), 5.91 (d, *J* = 8.4 Hz, 1H), 3.40 (dddd, *J* = 11.3, 9.2, 5.6, 3.4 Hz, 1H), 3.21 (s, 3H), 2.29 (dd, *J* = 17.3, 5.7 Hz, 1H), 2.04 (dd, *J* = 17.5, 9.3 Hz, 1H), 1.83 (ddd, *J* = 12.4, 3.5, 1.9 Hz, 1H), 1.71 (s, 3H), 1.53m (s, 3H), 1.16 – 1.08 (m, 1H), 0.97 (s, 6H) ppm.

***O*-Methylzaxinone 24**

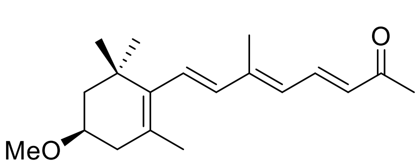

Following the general procedure for the aldol condensation, the reaction of (2*E*,4*E*)-5-((*R*)-4-methoxy-2,6,6-trimethylcyclohex-1-en-1-yl)-3-methylpenta-2,4-dienal **23** (0.030 g, 0.12 mmol), NaOH (0.011 mL, 1M in H_2O, 0.011 mmol) in acetone (1.5 mL) afforded, after purification by flash column chromatography (silica gel, from 97:3 *n*-hexane/Et_3N to 95:5 *n*-hexane/EtOAc), 0.025 g (74%) of a yellow oil, which was identified as *O*-methylzaxinone **24**. **^{1}H NMR** (400.13 MHz, C_6D_6): δ 7.54 (dd, *J* = 15.1, 11.7 Hz, 1H),

6.26 (d, J = 16.1 Hz, 1H), 6.14 (d, J = 16.1 Hz, 1H), 6.06 (d, J = 15.3 Hz, 1H), 6.00 (d, J = 11.9 Hz, 1H), 3.45 (dddd, J = 11.5, 9.1, 5.6, 3.4 Hz, 1H), 3.22 (s, 3H), 2.36 – 2.32 (m, 1H), 2.11 (dd, J = 17.2, 9.2 Hz, 1H), 1.93 (s, 3H), 1.91 – 1.84 (m, 1H), 1.69 (s, 3H), 1.66 (s, 3H), 1.51 – 1.48 (m, 1H), 1.05 (s, 6H) ppm.

Supplemental References

Alvarez, R., Vaz, B., Gronemeyer, H., and de Lera, A.R. (2014). Functions, therapeutic applications, and synthesis of retinoids and carotenoids. Chem. Rev. **114**:1-125.

Domínguez, M., Alvarez, R., Borrás, E., Farrés, J., Parés, X., and de Lera , A.R. (2006). Synthesis of Enantiopure C3- and C4-Hydroxyretinals and Their Enzymatic Reduction by ADH8 from Xeno pus laevis. Org. Biomol. Chem. **4**:155-164.

Fontán, N., Domínguez, M., Álvarez, R., and de Lera, Á.R. (2011). Synthesis of C40-Symmetrical Fully Conjugated Carotenoids by Olefin Metathesis. Eur. J. Org. Chem. 2011:6704-6712.

Gessler, N.N., Sokolov, A.V., and Belozerskaya, T.A. (2002). Initial Stages of Trisporic Acid Synthesis in Blakeslea trispora. App. Biochem. Microbiol. **38**:536-543.

Haag, A., and Eugster, C.H. (1982). Isozeaxanthine: Chiralität und enantioselektive Synthese von (4R,4′R)-Isozeaxanthin ((−)-(4R, 4′R)-β, β-Carotin-4,4′-diol). Helv. Chim. Acta **65**:1795-1803.

Ito, M., Hirata, Y., Shibata, Y., and Tsukida, K. (1990). Synthesis of the 4-alkylidenebutenolide carotenoids, peridinin and pyrrhoxanthin. J. Chem. Soc., Perkin Trans. **1**:197-199.

Ito, M., Matsuoka, N., Tsukida, K., and Seki, T. (1988). Retinoids and Related Compounds. X. : Synthesis of Geometrical Isomers of (±)-2- and (±)-3-Hydroxyretinals and Identification of the Chromophone of the Fly Visual Pigment. Chem. Pharm. Bull. **36**:78-86.

Katsuta, Y., et al. (1994). Synthesis of 9E- and 9Z-Locked Retinoic Acid Analogs as Ligands for RAR and RXR. Chem. Pharm. Bull **42**:2659-2661.

Maoka, T., Fujiwara, Y., Hashimoto, K., and Akimoto, N. (2001). Isolation of a Series of Apocarotenoids from the Fruits of the Red Paprika Capsicum annuum L. J. Agr. Food Chem. **49**:1601-1606.

Tanumihardjo, S.A. (2001). Synthesis of 10,11,14,15-13C4-and 14,15-13C2-retinyl acetate. J. Labelled Compd. Radiopharm. **44**:365-372.

Yamano, Y., and Ito, M. (1993). First total synthesis of (±)-peridinin, (±)-pyrrhoxanthin and the optically active peridinin. J. Chem. Soc. Perkin Trans. **1**:1599-1610.

Yamano, Y., Sumiya, S., and Ito, M. (1995). Carotenoids and related polyenes. Part 2. Photoisomerization of an allenic carotenoid, peridinin, and allenic model compounds. J. Chem. Soc, Perkin Trans. 1:167-176.
