# Supplementary material for: Efficient Mimics for Elucidating Zaxinone Biology and Promoting Agricultural Applications
Source: Mol Plant. 2020 Nov 2;13(11):1654–61. doi: 10.1016/j.molp.2020.08.009 (PMC7656291; doi:10.1016/j.molp.2020.08.009)
Supplement: Document S3. Protocols for the Synthesis of MiZax [file mmc3.pdf]

Efficient Mimics for Elucidating Zaxinone Biology and Promoting Agricultural Applications

Jian You Wang, Muhammad Jamil, Pei-Yu Lin, Tsuyoshi Ota, Valentina Fiorilli, Mara Novero, Randa A. Zarban, Boubacar A. Kountche, Ikuo Takahashi, Claudio Martínez, Luisa Lanfranco, Paola Bonfante, Angel R. de Lera, Tadao Asami and Salim Al-Babili

Supplementary Document 3

General procedure 1 (Wittig Reaction)

In a round-bottomed flask, the mixture of dimethylformamide (2.0 mL), aldehyde (1.0 mmol) and (acetylmethylene)triphenylphosphorane (4.0 mmol) was stirred for 2 h at 80ºC, then ethyl acetate (15 mL) was added to the reaction mixture, which was washed with water and brine. The organic layer was dried over anhydrous sodium sulfate and concentrated under reduced pressure, then purified by column chromatography on silica gel（Wakosil®C-300HG）, in which a mixture of hexane-ethylacetate was used as an eluent.

General procedure 2（Suzuki-Miyaura Cross-Coupling）

In a round-bottomed flask, the mixture of aryl bromide (2.0mmol), boronic acid (1.0 mmol), THF (15.0 mL), 2N Na_2CO_3 (4.5 mL) and tetrakis(triphenylphosphine)palladium(0) (0.01 mmol) were refluxed overnight with stirring, then THF was removed under reduced pressure. The resultant mixture was solved into ethyl acetate (15 mL), which was washed with water and brine. The organic layer was dried over anhydrous sodium sulfate and concentrated under reduced pressure, then purified by column chromatography on silica gel（Wakosil® C-300HG), in which a mixture of hexane-ethyl acetate was used as an eluent.

General procedure 3

In a round-bottomed flask, 1M boron tribromide in CH_2Cl_2 was added to the solution of methoxy-substituted aryl derivatives (1.0 mmol) in dichloromethane (5.0 mL) at 0 ºC and stirred for 1 h, then quenched with water (10 mL), diluted with CH_2Cl_2 (10 mL), and washed with brine. The organic layer was dried over anhydrous sodium sulfate and concentrated under reduced pressure, then purified by column chromatography on silica gel（Wakosil® C-300HG, in which a mixture of hexane-ethyl acetate was used as an eluent.

Chemical data

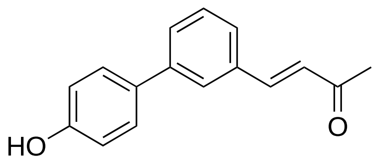

MiZax1. 3-Formylbenzeneboronic acid and 4-bromophenol were used as starting materials of procedure 2, then obtained compound was subjected to the procedure 1 to give MiZax-1 (47% yield).

^{1}H NMR (500MHz, CDCl3): δ 7.69(1H, s), 7.54-7.60(2H, m), 7.42-7.50(4H, m), 6.93(2H, d, J = 8.5Hz), 6.78(1H, d, J = 16.0Hz), 5.00(1H, s), 2.40(3H, s).

^{13}C NMR (126 MHz, $CDCl_3$) δ: 199.09, 155.79, 143.99, 141.66, 134.73, 132.75, 129.36, 128.99, 128.35, 128.35, 127.18, 126.67, 126.44, 115.86, 115.86, 27.52.

HRMS (ESI+) m/z = 239.1067 calculated for $C_{16}H_{15}O_2$ $[M+H]^+$, found: 239.1068. Melting point was 145-146°C.

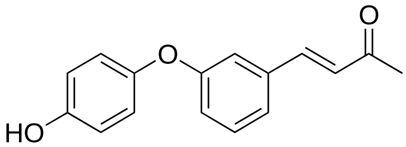

MiZax2. 3-(4-Methoxyphenoxy)benzaldehyde was used as a starting material of procedure 3, then obtained compound was subjected to the procedure 1 to give MiZax-2 (44% yield).

^{1}H NMR (500MHz, CDCl3): δ 7.43(1H, d, J = 16.5Hz), 7.32(1H, t, J = 8.0Hz), 7.21(1H, d, J = 8.0Hz), 7.07(1H, s), 6.98(1H, d, J = 8.0Hz), 6.94(2H, d, J = 8.5Hz), 6.84(2H, d, J = 8.5Hz), 6.63(1H, d, J = 16.5Hz), 4.88(1H, s), 2.36(3H, s).

^{13}C NMR (126 MHz, $CDCl_3$) δ: 199.61, 159.18, 152.69, 149.04, 143.80, 135.78, 130.13, 127.32, 122.42, 121.23, 121.23, 119.67, 116.54, 116.54, 116.45, 27.40.

HRMS (ESI+) m/z = 255.1016 calculated for $C_{16}H_{15}O_3$ $[M+H]^+$, found: 255.1011. Melting point was 92-95°C.

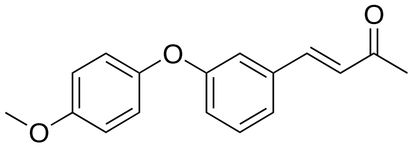

MiZax3. 3-(4-Methoxyphenoxy)benzaldehyde was used as a starting material of procedure 1 to give MiZax-3 (81% yield).

^{1}H NMR (500MHz, CDCl3): δ 7.42(1H, d, J = 16.5Hz), 7.30(1H, t, J = 8.0Hz), 7.19(1H, d, J = 8.0Hz), 7.06(1H, s), 6.95-7.00(3H, m), 6.89(2H, d, J = 7.0Hz), 6.61(1H, d, J = 16.5Hz), 6.63(1H, d, J = 16.5Hz), 3.81(1H, s), 2.34(3H, s).

^{13}C NMR (126 MHz, $CDCl_3$) δ: 198.11, 159.05, 156.11, 149.26, 142.71, 135.94, 130.01, 127.50, 122.31, 120.94, 120.94, 119.41, 116.22, 114.88, 114.88, 55.46, 27.32.

HRMS (ESI+) m/z = 269.1172 calculated for $C_{17}H_{17}O_3$ $[M+H]^+$, found: 269.1172. Melting point was 54-56°C.

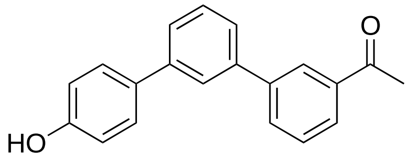

MiZax4. 1,3-Dibromobenzene was used as a starting material of procedure 2, in which 4-hydroxyphenylboronic acid and 3-acetylphenylboronic acid were sequentially subjected to the cross coupling to give MZ5, which was then subjected to the procedure 4 to give MiZax-4 (11% yield).

^{1}H NMR (500MHz, CDCl3): δ 8.23(1H, s), 7.96(1H, d, J = 8.0Hz), 7.40(1H, d, J = 8.0Hz), 7.76(1H, s), 7.49-7.59(6H, m), 6.94(2H, d, J = 9.0Hz), 2.68(3H, s).

^{13}C NMR (126 MHz, $CDCl_3$) δ: 199.06, 155.77, 141.79, 141.60, 140.52, 137.40, 133.18, 132.04, 129.27, 129.07, 128.40, 128.40, 127.34, 127.03, 126.18, 125.58, 125.43, 115.80, 115.80, 26.76.

HRMS (ESI+) m/z = 289.1223 calculated for $C_{20}H_{17}O_2$ $[M+H]^+$, found: 289.1232. Melting point was 47-50°C.

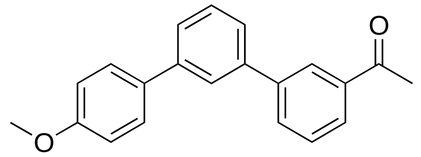

MiZax5. 1,3-Dibromobenzene was used as a starting material of procedure 2, in which 4-hydroxyphenylboronic acid and 3-acetylphenylboronic acid were sequentially subjected to the cross coupling to give MiZax-5 (75% yield).

^{1}H NMR (500MHz, CDCl3): δ 8.23(1H, s), 7.96(1H, d, J = 8.0Hz), 7.85(1H, d, J = 7.5Hz), 7.78(1H, s), 7.50-7.61(6H, m), 7.01(2H, d, J = 6.5Hz), 3.87(3H, s), 2.67(3H, s).

^{13}C NMR (126 MHz, $CDCl_3$) δ: 197.92, 159.25, 141.62, 141.45, 140.56, 137.53, 133.26, 131.70, 129.22, 128.96, 128.15, 128.15, 127.19, 126.86, 126.10, 125.53, 125.43, 114.18, 114.18, 55.21, 26.65.

HRMS (ESI+) m/z = 303.1380 calculated for $C_{21}H_{19}O_2$ $[M+H]^+$, found: 303.1376. Melting point was 80-81℃.
