# Supplementary material for: Efficient Mimics for Elucidating Zaxinone Biology and Promoting Agricultural Applications
Source: Mol Plant. 2020 Nov 2;13(11):1654–61. doi: 10.1016/j.molp.2020.08.009 (PMC7656291; doi:10.1016/j.molp.2020.08.009)
Supplement: Document S4. Article plus Supplemental Information [file mmc4.pdf]

CellPress
Partner Journal

Molecular Plant
Research Report

Efficient Mimics for Elucidating Zaxinone Biology and Promoting Agricultural Applications

Jian You Wang[1], Muhammad Jamil[1], Pei-Yu Lin[1], Tsuyoshi Ota[2], Valentina Fiorilli[3], Mara Novero[3], Randa A. Zarban[1], Boubacar A. Kountche[1], Ikuo Takahashi[2], Claudio Martínez[4], Luisa Lanfranco[3], Paola Bonfante[3], Angel R. de Lera[4], Tadao Asami[2,*] and Salim Al-Babili[1,*]

[1]King Abdullah University of Science and Technology, Division of Biological and Environmental Science and Engineering, the BioActives Lab, Thuwal 23955-6900, Saudi Arabia

[2]Graduate School of Agricultural and Life Sciences, The University of Tokyo, Tokyo, Japan

[3]Department of Life Sciences and Systems Biology, University of Torino, Torino, Italy

[4]Universidade de Vigo, Facultade de Química and CINBIO, Vigo, Spain

***Correspondence: Tadao Asami (asami@mail.ecc.u-tokyo.ac.jp), Salim Al-Babili (salim.babili@kaust.edu.sa)**

https://doi.org/10.1016/j.molp.2020.08.009

ABSTRACT

Zaxinone is an apocarotenoid regulatory metabolite required for normal rice growth and development. In addition, zaxinone has a large application potential in agriculture, due to its growth-promoting activity and capability to alleviate infestation by the root parasitic plant *Striga* through decreasing strigolactone (SL) production. However, zaxinone is poorly accessible to the scientific community because of its laborious organic synthesis that impedes its further investigation and utilization. In this study, we developed easy-to-synthesize and highly efficient mimics of zaxinone (MiZax). We performed a structure–activity relationship study using a series of apocarotenoids distinguished from zaxinone by different structural features. Using the obtained results, we designed several phenyl-based compounds synthesized with a high-yield through a simple method. Activity tests showed that MiZax3 and MiZax5 exert zaxinone activity in rescuing root growth of a zaxinone-deficient rice mutant, promoting growth, and reducing SL content in roots and root exudates of wild-type plants. Moreover, these compounds were at least as efficient as zaxinone in suppressing transcript level of SL biosynthesis genes and in alleviating *Striga* infestation under greenhouse conditions, and did not negatively impact mycorrhization. Taken together, MiZax are a promising tool for elucidating zaxinone biology and investigating rice development, and suitable candidates for combating *Striga* and increasing crop growth.

Key words: apocarotenoids, zaxinone, zaxinone mimics, strigolactone, *Striga*, root parasitic plants

Wang J.Y., Jamil M., Lin P.-Y., Ota T., Fiorilli V., Novero M., Zarban R.A., Kountche B.A., Takahashi I., Martínez C., Lanfranco L., Bonfante P., de Lera A.R., Asami T., and Al-Babili S. (2020). Efficient Mimics for Elucidating Zaxinone Biology and Promoting Agricultural Applications. Mol. Plant. **13**, 1654–1661.

INTRODUCTION

Chemical signals and hormones are involved in literally all aspects of a plant's life. These small molecules are key regulators of plant development and response to environmental stimuli, and the means of communication between plants and surrounding organisms (Chaiwanon et al., 2016; Guerrieri et al., 2019). Strigolactones (SLs) are an intriguing example for signaling molecules that fulfill both functions. They act as a hormone that determines diverse processes within plant, which include shoot branching, growth of primary, lateral and adventitious roots, and biotic and abiotic stress responses (Al-Babili and Bouwmeester, 2015; Waters et al., 2017; Jia et al., 2018). In addition, SLs are released into the rhizosphere, particularly under phosphate starvation, as signaling molecules that facilitate the recruitment of arbuscular mycorrhizal (AM) fungi for establishing the beneficial AM symbiosis (Bonfante and Genre, 2008; Gutjahr and Parniske, 2013; Lanfranco et al., 2018). However, obligate root parasitic plants of the *Orobanchaceae* family have evolved specific receptors that trigger the germination of their seeds upon perceiving rhizospheric SLs. This mechanism enables synchronizing the germination with the availability of a host in close neighborhood, which ensures the survival of the arising parasite

Published by the Molecular Plant Shanghai Editorial Office in association with Cell Press, an imprint of Elsevier Inc., on behalf of CSPB and IPPE, CAS.

This is an open access article under the CC BY license (http://creativecommons.org/licenses/by/4.0/).

seedling (Xie and Yoneyama, 2010). Root parasitic plants, such as *Striga* spp., are a severe agricultural problem in warm and temperate zones (Parker, 2012). Indeed, *Striga hermonthica* that infests cereals, such as rice, sorghum, pearl millet, and maize, is one of the major threats to global food security, as it causes enormous yield losses in different regions of Africa (Pennisi, 2010; Parker, 2012).

SLs consist of a butenolide ring (D-ring) that is connected by an enol bridge of (*R*)-configuration to a less structurally conserved second moiety (Al-Babili and Bouwmeester, 2015; Jia et al., 2018). SLs derive from carotenoids, essential isoprenoid photosynthetic pigments equipped with conjugated double bonds varying in their stereo-configuration (Moise et al., 2014). The enzyme DWARF27 in rice and orthologs from other plants initiate SL biosynthesis by isomerizing all-*trans*- to 9-*cis*-β-carotene (Bruno and Al-Babili, 2016; Abuauf et al., 2018), which is subjected in the next step to a stereospecific cleavage catalyzed by the carotenoid cleavage dioxygenase 7 (CCD7) that forms the volatile β-ionone and a 9-*cis*-configured apocarotenoid intermediate (Bruno et al., 2014). The latter is then converted by CCD8 via a combination of repeated oxygenation and other less understood reactions into the central SL biosynthesis intermediate carlactone (Alder et al., 2012; Bruno et al., 2017). In the next steps, cytochrome P450s, such as the *Arabidopsis* MAX1 or the rice carlactone oxidase (CO), together with other enzymes transform carlactone into different SLs, giving rise to the structural diversity of these compounds (Abe et al., 2014; Zhang et al., 2014; Brewer et al., 2016; Yoneyama et al., 2018; Wakabayashi et al., 2019).

Besides SLs and abscisic acid, carotenoids are the precursor of several regulatory metabolites, including cyclocitral, zaxinone, and anchorene (Dickinson et al., 2019; Jia et al., 2019; Wang et al., 2019). Recently, we showed that the apocarotenoid, i.e., carotenoid cleavage product zaxinone, is a common plant metabolite that determines rice growth and development (Wang et al., 2019). Zaxinone biosynthesis is catalyzed in rice by the zaxinone synthase (ZAS), a member of a less-characterized plant CCD subfamily (Wang et al., 2019). The rice *zas* mutant shows growth retardation, lower zaxinone levels in roots, and higher SL content in roots and root exudates. These phenotypes could be rescued, to a large extent, by exogenous application of synthetic zaxinone that promoted root growth and reduced SL content and release also in wild-type plants. Expression analysis of treated *zas* and wild-type plants suggested that zaxinone suppressed the transcript level of SL biosynthetic genes under phosphate starvation. Moreover, application of zaxinone to rice plants under greenhouse conditions significantly decreased *Striga* emergence, likely by lowering SL release. These results demonstrate the importance of zaxinone for basic plant science as well its application potential for improving crop growth, regulating shoot branching, and controlling *Striga*. However, further investigation of the biological functions of zaxinone, its interaction with plant hormones, as well as its application potential are hampered by the laborious synthesis (see Supplemental Figure 1) of this compound, which makes it poorly accessible to the scientific community.

Analogs and mimics of hormones are frequently used in basic research as well as in agricultural and horticultural applications (Rigal et al., 2014; Koprna et al., 2016; Screpanti et al., 2016). This is particularly the case if the bioactivity of the authentic metabolite is short living (Rigal et al., 2014; Vaidya et al., 2019) or if its natural sources are restricted and organic synthesis is complicated. SLs are a best example for the latter case. The scarcity of SLs has prompted researchers to use mimics and analogs, mainly GR24, which have been decisive in elucidating SL biology and even in the discovery of the SL hormonal function (Gomez-Roldan et al., 2008; Umehara et al., 2008; Al-Babili and Bouwmeester, 2015). Similarly, agricultural applications of SLs, such as inducing suicidal germination of root parasitic weeds, rely on different analogs (Samejima et al., 2016; Vurro et al., 2016; Jamil et al., 2018, 2019, 2020; Kountche et al., 2019).

In this work, we developed the first reported series of zaxinone mimics. For this purpose, we first performed a structure–activity relationship study that allowed us to identify structural features required for zaxinone activity. Next, we designed easy-to-synthesize mimics of zaxinone (MiZax) and characterized their biological activities in regulating SL biosynthesis and rice growth, and alleviating *Striga* infestation. Results obtained demonstrate the efficiency of these MiZax and their utility for zaxinone-related studies and applications.

RESULTS AND DISCUSSION

Chain Length, Stereo-Configuration, and the Ketone Functional Group Are Essential for Zaxinone Activity

Identifying structural elements required for activity is a crucial step in rational design of hormone analogs/mimics. Zaxinone is a C_{18}-ketone consisting of a linear, all-*trans*-configured isoprenoid polyene linked to a β-ionone ring carrying a hydroxy group at the C3 position (Figure 1A). The functional ketone group of zaxinone is separated from the β-ionone ring by a chain with a length of six C atoms. To perform the structure–activity relationship study, we synthesized a series of apocarotenoids that differ from zaxinone in the polyene length, its stereo-configuration, the type of the ionone ring, or the position of the hydroxy group. We also synthesized zaxinol, in which we replaced the ketone of zaxinone by a hydroxy group, and D'orenone that lacks the hydroxy group at C3 position (Figure 1A). Next, we applied these compounds to hydroponically grown wild-type seedlings exposed to 1 week Pi starvation, and quantified 4-deoxyorobanchol, a major rice SL, in roots and root exudates, using liquid chromatography-tandem mass spectrometry (LC-MS/MS). The shorter apocarotenoids 3-OH-β-cyclocitral and 3-OH-β-ionone, the *cis*-configured 9-*cis*-zaxinone, zaxinol, and 4-OH-β-apo-13-carotenone did not significantly impact 4-deoxyorobanchol content in roots or root exudates (Supplemental Figure 2), suggesting that chain length, stereo-configuration, the presence of the ketone group, and the position of the hydroxy group are important for exerting zaxinone activity. In contrast, the application of α-zaxinone and, particularly, D'orenone decreased SL content to levels comparable with those observed upon treatment with zaxinone, with the latter being the most efficient compound followed by D'orenone (Figure 1B). These data suggest that all-*trans*-C_{13}-apocarotenones (C_{18}-ketones) can generally repress SL production and that the presence of the hydroxy

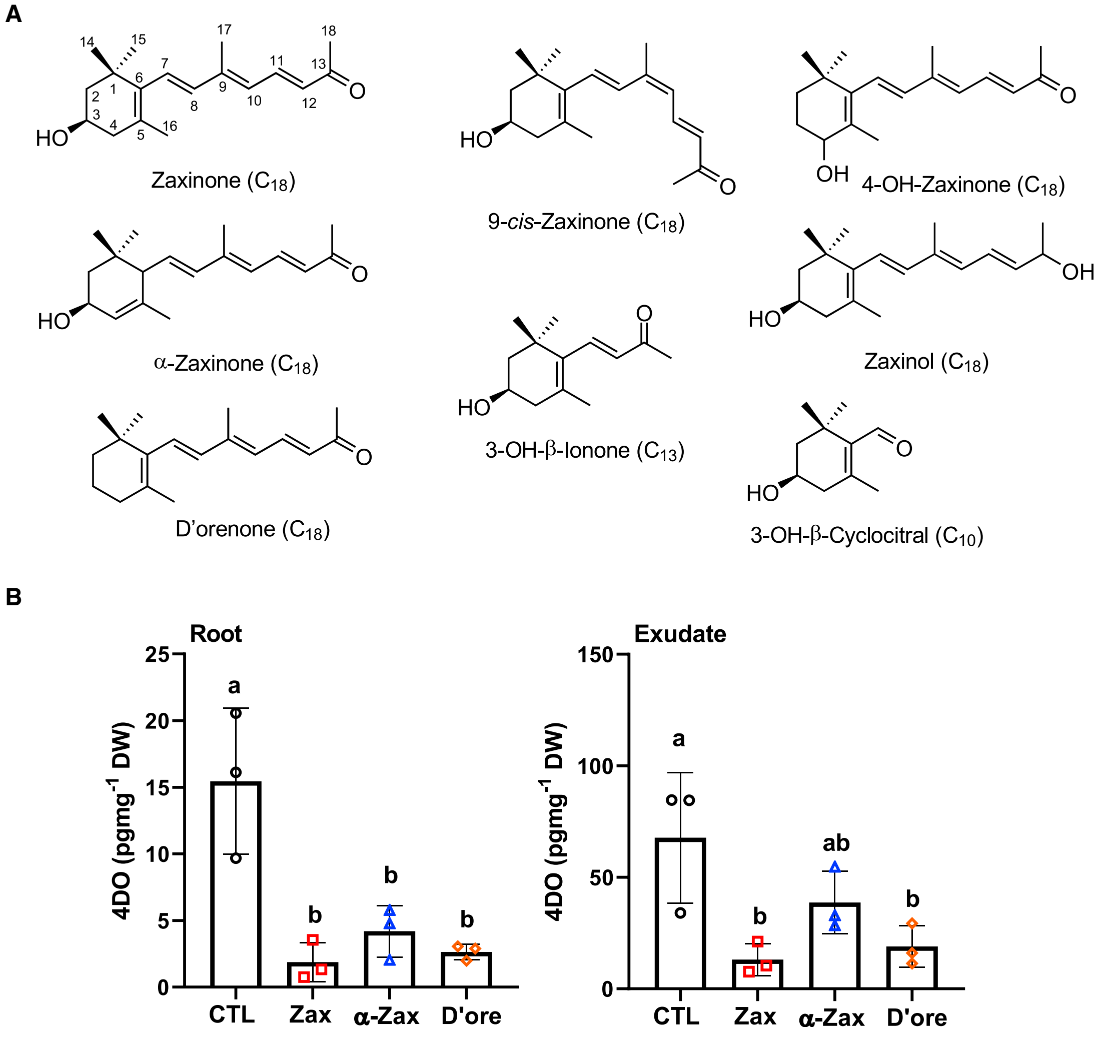

Figure 1. Structure and Effect of Apocarotenoids on SL Content in Root Tissues and Exudates.
(A) Structures of apocarotenoids used in the structure–activity relationship study.
(B) SL quantification (4-deoxyorobanchol [4-DO]) in wild-type root tissues and exudates in response to zaxinone (Zax), α-zaxinone (α-Zax), and D'orenone (D'ore) treatment (5 μM) under Pi starvation. Bars represent mean ± SD; $n = 3$ biological replicates; statistical analysis was performed using one-way analysis of variance (ANOVA) and Tukey's *post hoc* test. Different letters denote significant differences ($P < 0.05$). CTL, control; Zax, zaxinone; α-Zax, α-zaxinone; and D'ore, D'orenone.

group and the position of the double bond in the ionone ring have less impact on this activity.

Synthesis and Screening of Zaxinone Mimics

Zaxinone is a natural apocarotenoid characterized by a conjugated isoprenoid chain. The synthesis of zaxinone requires five steps and has a moderate yield (47% or less; Supplemental Figure 1). We aimed at the development of efficient MiZax, which can be synthesized in significantly fewer steps and at higher yield. To achieve this goal, we relied on the results of the structure–activity relationship study and decided to substitute the conjugated isoprenoid chain of zaxinone by aromatic structures. This was inspired by several successful examples, such as the development of the fungicides azoxystrobin and metominostrobin (Bartlett et al., 2002) and the insecticide fenoxycarb from natural isoprenoid bioactive compounds (Thind and Edwards, 1986). We also chose the replacement of the β-ionone ring of zaxinone by a phenyl ring, which is a common approach in designing SL analogs (Boyer et al., 2014; Jia et al., 2016; Takeuchi et al., 2018). To evaluate the biological activity of the designed mimics, we determined their effect on SL content in root exudates, using LC-MS/MS quantification or *Striga* seed germination as a bioassay. In a first attempt, we designed MiZax1 that contains phenyl rings instead of the β-ionone ring and part of the conjugated chain (Supplemental Figure 3A). MiZax1 was synthesized in only two steps. However, application of this compound did not significantly impact the SL level in root exudates of treated rice plants (Supplemental Figure 3B). The distance between the ketone group and the phenyl ring in MiZax1 is five C atoms, i.e., one C atom shorter than in zaxinone. Hence, we hypothesized that the missing activity of this mimic might be a result of the short chain length. Therefore, we designed further four mimics (Figure 2A) in which the phenyl ring and the ketone group are separated by a chain of six atoms, and the zaxinone isoprenoid

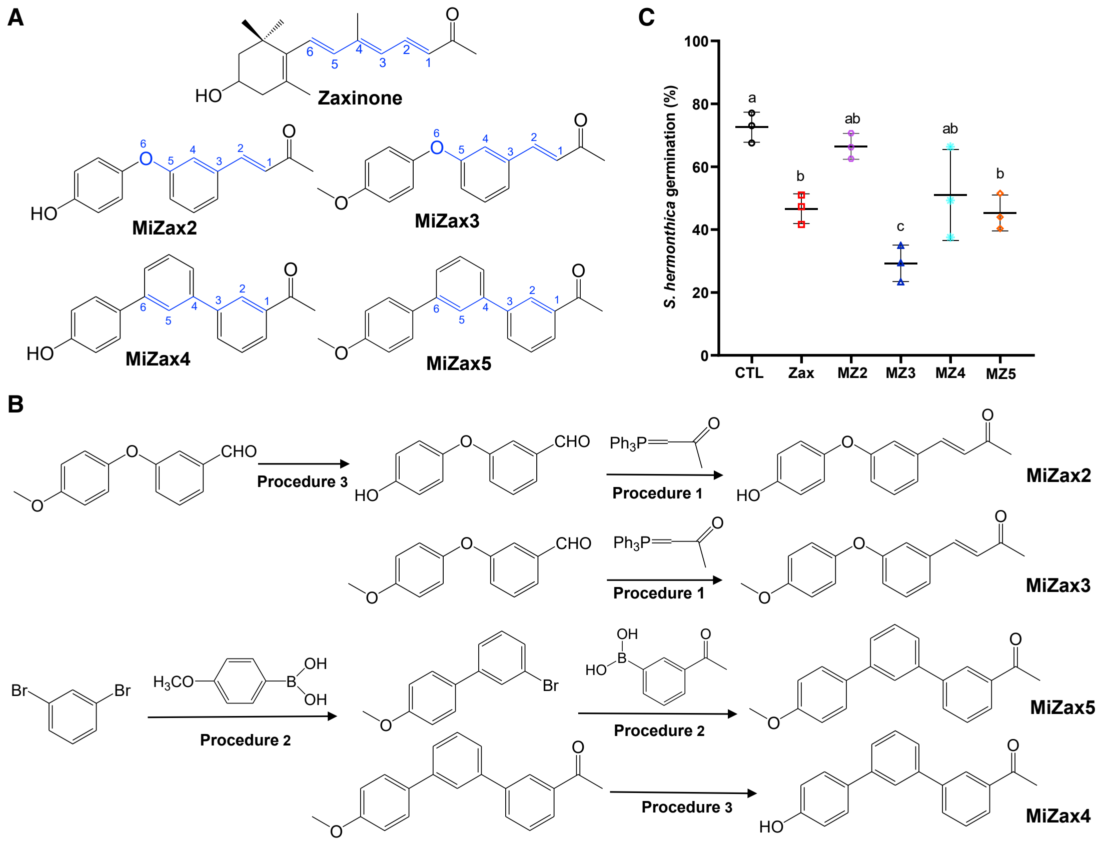

Figure 2. Synthesis and Screening of MiZax.
(A) Chemical structure of zaxinone and MiZax2-5.
(B) Synthesis scheme of MiZax, (*E*)-4-(3-(4-hydroxyphenoxy)phenyl)but-3-en-2-one (MiZax2), (*E*)-4-(3-(4- methoxyphenoxy)phenyl)but-3-en-2-one (MiZax3), 1-(4″-hydroxy-[1,1':3′,1″-terphenyl]-3-yl)ethan-1-one (MiZax4), and 1-(4″-methoxy-[1,1':3′,1″-terphenyl]-3-yl)ethan-1-one (MiZax5). Numbers in blue indicate the distance between phenyl ring and the ketone group. The detailed synthetic methods are provided in Supplemental Document S3.
(C) *Striga* seed germination activity of rice root exudates isolated from plants treated with zaxinone or MiZax2-5. Bars represent means ± SD; $n = 3$ biological replicates. Statistical analysis was performed using ANOVA and Tukey's *post hoc* test. Different letters denote significant differences ($P < 0.05$). CTL, control; Zax, zaxinone; MZ2, MiZax2; MZ3, MiZax3; MZ4, MiZax4; MZ5, MiZax5.

chain is substituted by two phenyl rings (MiZax4 and -5) or partially replaced by a phenoxy ring (MiZax2 and -3). The hydroxy group in MiZax3 and MiZax5 was methylated, to increase their hydrophobicity and account for methylation as a possible zaxinone modification *in planta* (Figure 2A). The four mimics were synthesized in one or two steps (Figure 2B), with yield rates ranging from 11% (MiZax4) to 81% (MiZax3).

To test the hypothesis on the effect of the chain length, we measured the SL content in root exudates of rice plants treated with MiZax1, MiZax2, or MiZax4. In comparison with MiZax1 and mock control and supporting our hypothesis, application of MiZax2 led to a significant decrease in SL level and *Striga* germination rate, while MiZax4 showed a tendency to reduce SLs, particularly orobanchol, release (Supplemental Figure 4). Besides a common chain length, MiZax3 and MiZax5 contain a methoxy group instead of the hydroxy group at C3 in zaxinone and the corresponding position in MiZax2 and MiZax4. Comparison of the effect of MiZax2 and MiZax3, and of MiZax4 and MiZax5, on *Striga* seed-germinating activity demonstrated that this methylation has a significant positive effect on the activity of zaxinone mimics (Figure 2C). Hence, we speculated that zaxinone is converted into methyl-zaxinone *in planta*. To test this possibility, we synthesized methyl-zaxinone and checked its presence *in planta* as well as its biological activity. However, we could not detect methyl-zaxinone in rice plants (data not shown). In addition, the biological efficiency of methyl-zaxinone in inducing *Striga* seed germination was similar to that of zaxinone (Supplemental Figure 5), indicating that the presence of the methoxy group *per se* is not the reason of the increased activity of MiZax3 and MiZax5 and that direct zaxinone methylation might not take place *in planta*. Supporting the latter conclusion, we did not detect a conversion of MiZax2 or MiZax4 into MiZax3 or MiZax5, respectively, in rice plants fed with the former two mimics (Supplemental Figure 6). These data indicate that the higher activity observed with MiZax3 and MiZax5 could be a result of increased hydrophobicity caused by the methyl group, which may improve their uptake and transport. Indeed, we detected MiZax3 and MiZax5 in shoots of rice plants fed with these compounds through roots using LC-MS analysis (Supplemental Figure 7). We also observed a positive effect of the presence of a phenoxy group in MiZax3 instead of the unmodified phenyl ring in

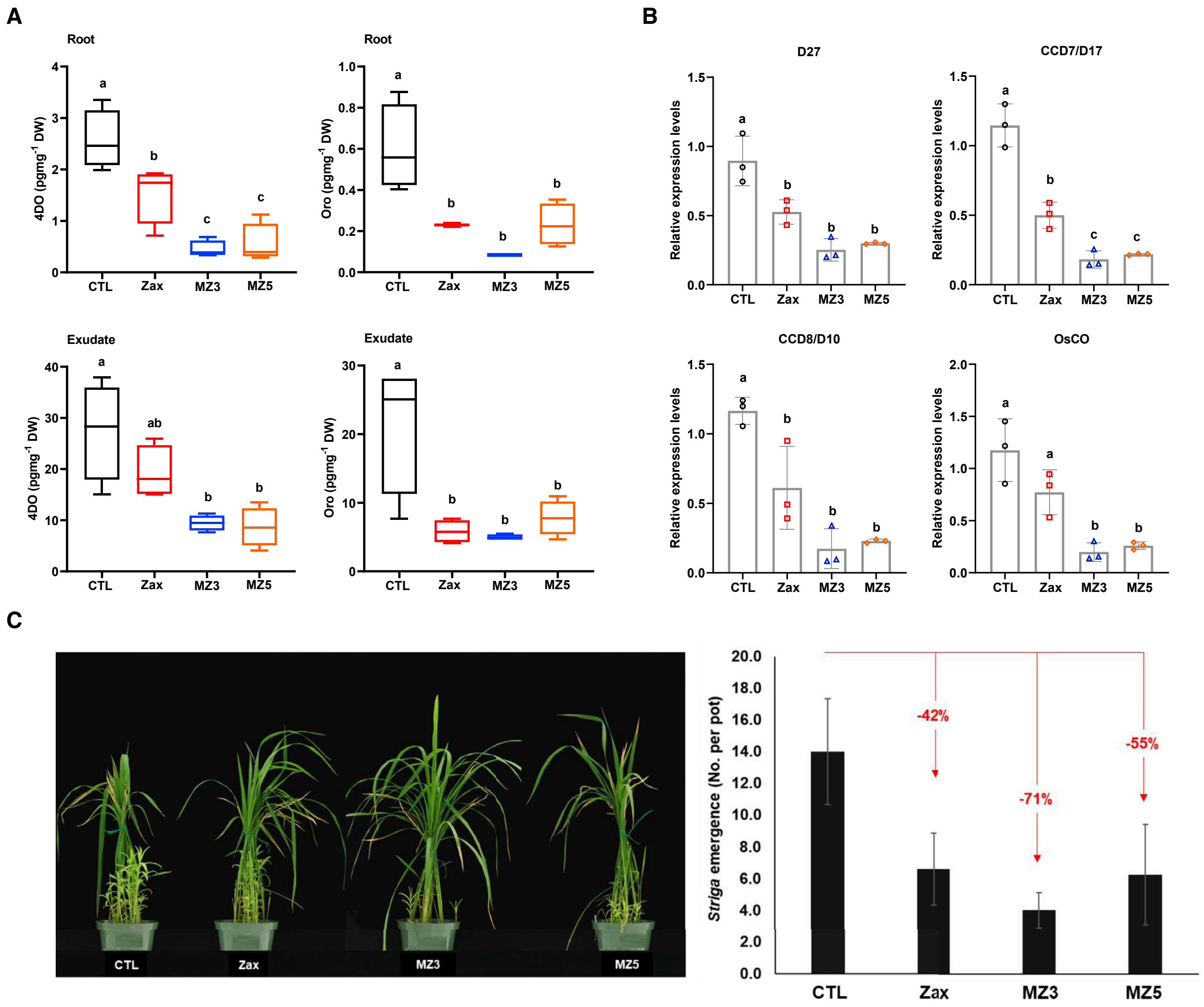

Figure 3. Effect of MiZax3 and MiZax5 on SL Biosynthesis and Release.

(A) Quantification of the SLs 4-deoxyorobanchol (4-DO) and orobanchol (Oro) in rice roots and root exudates in response to zaxinone, MZ3, and MZ5 application (5 μM) under Pi starvation. Bars represent means ± SD, *n* = 4 biological replicates.

(B) Relative transcript levels of SL biosynthesis genes (*D27*, *CCD7*, *CCD8*, and *CO*) in response to zaxinone, MZ3, and MZ5 application. Transcript levels in wild-type control samples were normalized to 1. Bars represent means ± SD, *n* = 3 biological replicates.

(C) *Striga* infestation in rice in response to zaxinone, MZ3, and MZ5 treatment (5 μM). Bars represent mean ± SE; *n* = 4 biological replicates. Statistical analysis was performed using ANOVA and Tukey's *post hoc* test. CTL, control; Zax, zaxinone; MZ3, MiZax3; MZ5, MiZax5.

MiZax5. This difference might be due to an increased stability caused by a shorter conjugated double bond system and/or the ether bond. To check this assumption, we determined the stability of MiZax3 and MiZax5. For this purpose, we monitored the degradation of these compounds for up to 2 weeks by high-performance liquid chromatography quantification of corresponding aqueous samples kept at room temperature. This study showed that MiZax3 is much more stable than MiZax5 (Supplemental Figure 8), which might explain its higher activity.

MiZax3 and MiZax5 Are Negative Regulators of Rice SL Biosynthesis and Release

We evaluated the zaxinone activity of the four mimics using *Striga* seed germination assay performed with root exudates of rice plants treated with 5 μM of each MiZax. Results obtained unraveled a significant negative impact of MiZax3 and MiZax5 treatment on *Striga* seed-germinating activity, which was not observed with MiZax2 and MiZax4 (Figure 2C). Exudates of MiZax3-treated plants showed lowest seed-germinating activity (29%) followed by zaxinone (45%) and MiZax5 (46%). Next, we measured orobanchol and 4-deoxyorobanchol content in root tissues and exudates of hydroponically grown, Pi-starved wild-type seedlings after treatment with 5 μM MiZax3 or MiZax5 for 6 h using LC-MS/MS. Application of both mimics decreased the level of the two SLs in both roots and root exudates (Figure 3A). The effect of MiZax3 or MiZax5 was similar to that of zaxinone and even significantly stronger in the case of 4-deoxyorobanchol. The two mimics, particularly MiZax3, rescued the high SL phenotype of the rice *zas* mutant (Figure 4A;

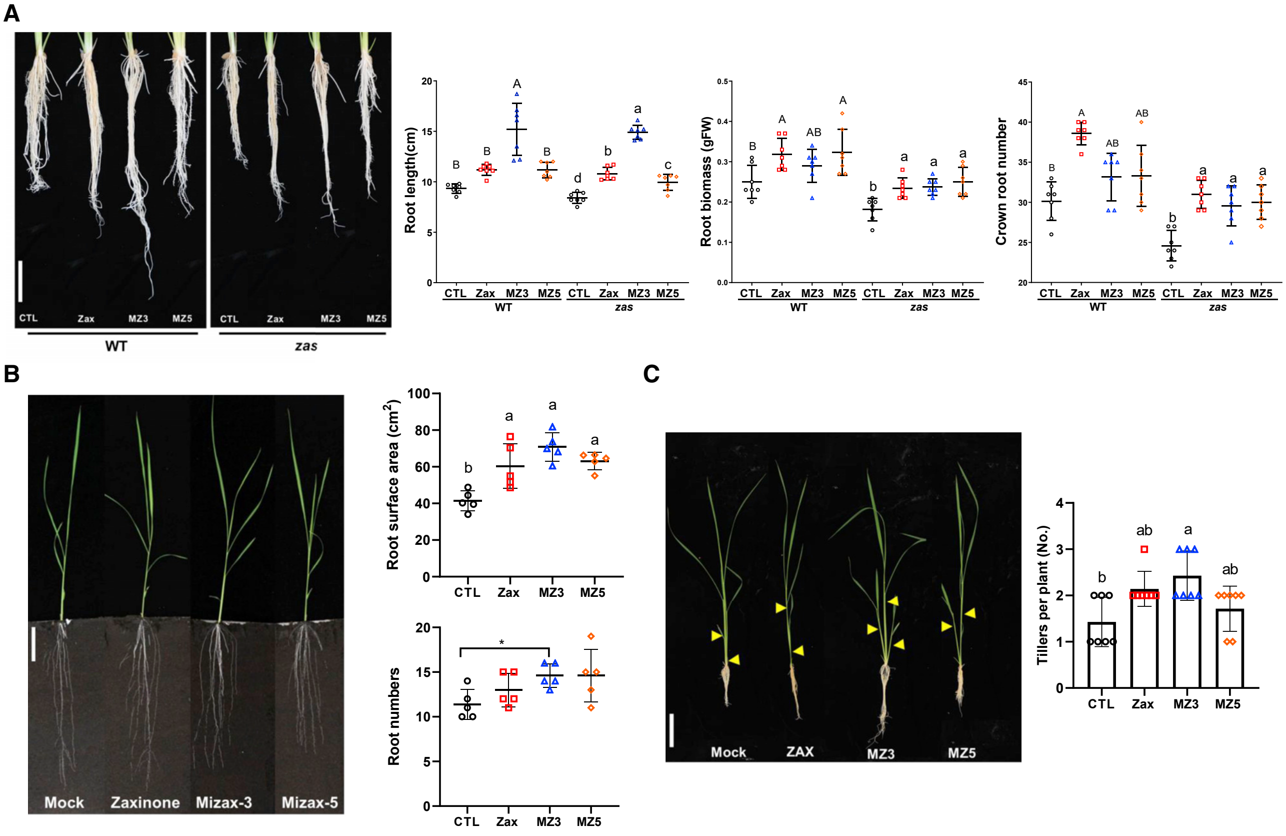

Figure 4. Effect of MiZax3 and MiZax5 on Rice Growth.

(A) Effect of zaxinone, MZ3, and MZ5 application (2.5 μM) on root growth of wild-type and *zas* mutant seedlings grown under hydroponic conditions. Scale bars correspond to 2 cm.

(B) Effect of zaxinone, MZ3, and MZ5 application (5 μM) on rice root growth under rhizotron conditions. Scale bars correspond to 8 cm.

(C) Effect of zaxinone, MZ3, and MZ5 application (2.5 μM) on rice tillering. Tillers are indicated by yellow arrows points. Scale bars correspond to 6 cm. Each data point represents one plant **(A)** $n = 6$; **(B)** $n = 5$; **(C)** $n = 7$. Data represent mean ± SD. Statistical analysis was performed using ANOVA and Tukey's *post hoc* test or *t*-test. Different letters denote significant differences ($P < 0.05$). CTL, control; Zax, zaxinone; MZ3, MiZax3; MZ5, MiZax5.

Supplemental Figure 9), similar to zaxinone (Wang et al., 2019). Subsequently, we determined the transcript level of the SL biosynthetic genes *D27*, *CCD7*, *CCD8*, and *CO* in roots from the same experiment. Application of MiZax3 and MiZax5 led to a pronounced decrease in the transcript level of the four enzymes, which was—at least in the case of *CCD7* and *CO* transcripts—significantly lower than that observed with zaxinone (Figure 3B).

The high activity of MiZax3 and MiZax5 in suppressing SL biosynthesis and release indicated their potential in combating *Striga* and other root parasitic weeds, similar to zaxinone. To test this hypothesis, we applied the two mimics at a 5 μM concentration to the *Striga* susceptible cv. IAC-165 rice plants grown in *Striga*-infested soil under greenhouse conditions. Treatment with these compounds led to a clear reduction in the number of emerging *Striga* plants, with the highest reduction observed with MiZax3 (71%), followed by MiZax5 (55%) and zaxinone (42%) (Figure 3C). Considering the important role of SL in the establishment of the AM symbiosis (Fiorilli et al., 2019), we checked the impact of MiZax on AM spore germination and on the colonization process. For this purpose, we treated *Gigaspora margarita* spores with MiZax3 and MiZax5 at a concentration of 5 μM or 50 nM, using the SL analog GR24 (10 nM) as a positive control. After 3 days incubation, GR24 induced, as expected, the germination rate, while no effect was observed for the two mimics (Supplemental Figures 10 and 11), as with zaxinone treatment (Supplemental Figure 12). We also did not detect any alteration in intraradical fungal structures or colonization rate (Supplemental Figure 13A and 13B). In line with this result, the expression levels of the AM marker genes *OsPt11* and *OsLysM* (Fiorilli et al., 2015), and the fungal housekeeping gene (*Fm18S rRNA*) did not show any significant difference between control and 5 μM treated plants. The 50nM treatment even induced a slight upregulation of these AM marker genes (Supplemental Figure 13C). These results indicate that the two mimics would not have a negative side effect on AM fungi and mycorrhization if applied 10 days after inoculation (see Supplemental Methods).

MiZax Exert Zaxinone Activity in Regulating Rice Growth and Development

Apart from regulating SL biosynthesis and release, zaxinone mimics should be able to rescue growth retardation of the rice *zas* mutant and promote the growth of wild-type plants. To check the capability of MiZax3 and MiZax5 in regulating rice growth, we

exposed hydroponically grown *zas* and wild-type (cv. Nipponbare) seedlings to 2.5 μM MiZax3, MiZax5, or zaxinone for 3 weeks. Similar to zaxinone, treatment with the two mimics promoted root growth in wild-type seedlings, by increasing root length and number of crown roots, and rescued root-related *zas* phenotypes, including root biomass (Figure 4A). The two MiZax also triggered root growth of wild-type plants. Moreover, MiZax3 was more active than zaxinone in increasing the root length of wild-type plants in the hydroponic system (Figure 4A). Next, we investigated the effect of the two MiZax in soil at a 5 μM concentration, using the rhizotron system and in comparison with zaxinone. MiZax3 and MiZax5 increased root surface area and the number of crown roots in wild-type plants, similar to zaxinone (Figure 4B). Tillering is a SL-dependent developmental process affected by zaxinone, as shown for the *zas* mutant (Wang et al., 2019). To check if MiZax3 and MiZax5 can also regulate tillering, we exposed rice Nipponbare and IAC-165 (a high SL-producing cultivar) seedlings to these two mimics for 14 days and determined tillers number. We observed a clear promotion of tillers number in both cultivars upon treatment with MiZax3 and a tendency toward more tillers with zaxinone and MiZax5 (Figure 4C and Supplemental Figure 14), which is in line with decreased SL production (Figure 3B). Although MiZax are promising candidates for promoting crop growth and alleviating *Striga* infestation, further studies about their health safety and environmental impact are needed.

In summary, we have developed two high-efficient mimics of zaxinone, which will pave the way for a better understanding of rice growth and development, and the role of zaxinone in this complex process. Moreover, the pronounced activity, simple synthesis (one step, Figure 2B) and relative stability of MiZax3 make it an excellent candidate for different sustainable agricultural applications, including the use of the beneficial AM fungi and the control of *Striga* that severely threatens global food security.

METHODS

Detailed methods are available in Supplemental Document 1.

SUPPLEMENTAL INFORMATION

Supplemental Information can be found online at *Molecular Plant Online*.

FUNDING

This work was supported by the Bill & Melinda Gates Foundation (grant no. OPP1194472) and a Competitive Research Grant (CRG2017) to S.A.-B. from King Abdullah University of Science and Technology (KAUST), and by grants from the Core Research for Evolutional Science and Technology (CREST) Program and the SATREPS Program of the Japan Science and Technology Agency (JST), and JSPS Grant-in-Aid for Scientific Research (grant no. 18H03939) to T.A.

AUTHOR CONTRIBUTIONS

S.A.-B., and T.A. proposed the concept and designed the experiments. J.Y.W., M.J., P.-Y.L., V.F., M.N., R.A.Z., and B.A.K. performed the experiments. T.A. designed and synthesized MiZax. T.O. and I.T. synthesized MiZax. C.M. and A.R.de.L. synthesized apocarotenoids used for the structure–activity relationship experiments. J.Y.W., V.F., L.L., P.B., T.A., and S.A.-B. analyzed the data. S.A.-B. and J.Y.W. wrote the manuscript. All authors read and approved the manuscript.

ACKNOWLEDGMENTS

We thank Dr. Abdel Gabar Babiker for providing *S. hermonthica* seeds, Dr. Ikram Blilou for supervising the rhizotron experiment, and to all the members of the BioActives lab at KAUST for their helpful discussions. We also thank the members of KAUST Plant Growth Core Lab and the Analytical Chemistry Core Lab for their kind support. King Abdullah University of Science and Technology (KAUST) and The University of Tokyo have applied for a patent on zaxinone mimics (MiZax) and their applications. No conflict of interest declared.

Received: March 8, 2020
Revised: July 7, 2020
Accepted: August 19, 2020
Published: August 20, 2020

REFERENCES

Abe, S., Sado, A., Tanaka, K., Kisugi, T., Asami, K., Ota, S., Kim, H.I., Yoneyama, K., Xie, X., Ohnishi, T., et al. (2014). Carlactone is converted to carlactonoic acid by MAX1 in *Arabidopsis* and its methyl ester can directly interact with AtD14 in vitro. Proc. Natl. Acad. Sci. U S A **111**:18084–18089.

Abuauf, H., Haider, I., Jia, K.P., Ablazov, A., Mi, J., Blilou, I., and Al-Babili, S. (2018). The *Arabidopsis* DWARF27 gene encodes an all-*trans*-/9-*cis*-β-carotene isomerase and is induced by auxin, abscisic acid and phosphate deficiency. Plant Sci. **277**:33–42.

Al-Babili, S., and Bouwmeester, H.J. (2015). Strigolactones, a novel carotenoid-derived plant hormone. Annu. Rev. Plant Biol. **66**:161–186.

Alder, A., Jamil, M., Marzorati, M., Bruno, M., Vermathen, M., Bigler, P., Ghisla, S., Bouwmeester, H., Beyer, P., and Al-Babili, S. (2012). The path from beta-carotene to carlactone, a strigolactone-like plant hormone. Science **335**:1348–1351.

Bartlett, D.W., Clough, J.M., Godwin, J.R., Hall, A.A., Hamer, M., and Parr-Dobrzanski, B. (2002). The strobilurin fungicides. Pest Manag. Sci. **58**:649–662.

Bonfante, P., and Genre, A. (2008). Plants and arbuscular mycorrhizal fungi: an evolutionary-developmental perspective. Trends Plant Sci. **13**:492–498.

Boyer, F.D., de Saint Germain, A., Pouvreau, J.B., Clavé, G., Pillot, J.P., Roux, A., Rasmussen, A., Depuydt, S., Lauressergues, D., and Frei Dit Frey, N. (2014). New strigolactone analogs as plant hormones with low activities in the rhizosphere. Mol. Plant **7**:675–690.

Brewer, P.B., Yoneyama, K., Filardo, F., Meyers, E., Scaffidi, A., Frickey, T., Akiyama, K., Seto, Y., Dun, E.A., Cremer, J.E., et al. (2016). LATERAL BRANCHING OXIDOREDUCTASE acts in the final stages of strigolactone biosynthesis in *Arabidopsis*. Proc. Natl. Acad. Sci. U S A **113**:6301–6306.

Bruno, M., Vermathen, M., Alder, A., Wüst, F., Schaub, P., Van-der-Steen, R., Beyer, P., Ghisla, S., and Al-Babili, S. (2017). Insights into the formation of carlactone from in-depth analysis of the CCD8-catalyzed reactions. FEBS Lett. **591**:792–800.

Bruno, M., and Al-Babili, S. (2016). On the substrate specificity of the rice strigolactone biosynthesis enzyme DWARF27. Planta **243**:1429–1440.

Bruno, M., Hofmann, M., Vermathen, M., Alder, A., Beyer, P., and Al-Babili, S. (2014). On the substrate- and stereospecificity of the plant carotenoid cleavage dioxygenase 7. FEBS Lett. **588**:1802–1807.

Chaiwanon, J., Wang, W., Zhu, J.Y., Oh, E., and Wang, Z.Y. (2016). Information integration and communication in plant growth regulation. Cell **164**:1257–1268.

Dickinson, A.J., Lehner, K., Mi, J., Jia, K.P., Mijar, M., Dinneny, J., Al-Babili, S., and Benfey, P.N. (2019). β-Cyclocitral is a conserved root growth regulator. Proc. Natl. Acad. Sci. U S A **116**:10563–10567.

Fiorilli, V., Wang, J.W., Bonfante, P., Lanfranco, L., and Al-Babili, S. (2019). Apocarotenoids: old and new mediators of the arbuscular mycorrhizal symbiosis. Front. Plant Sci. **10**:1186.

Fiorilli, V., Vallino, M., Biselli, C., Faccio, A., Bagnaresi, P., and Bonfante, P. (2015). Host and non-host roots in rice: cellular and molecular approaches reveal differential responses to arbuscular mycorrhizal fungi. Front. Plant Sci. **6**:636.

Gomez-Roldan, V., Fermas, S., Brewer, P.B., Puech-Pages, V., Dun, E.A., Pillot, J.P., Letisse, F., Matusova, R., Danoun, S., Portais, J.C., et al. (2008). Strigolactone inhibition of shoot branching. Nature **455**:189–194.

Guerrieri, A., Dong, L., and Bouwmeester, H.J. (2019). Role and exploitation of underground chemical signaling in plants. Pest Manag. Sci. **75**:2455–2463.

Gutjahr, C., and Parniske, M. (2013). Cell and developmental biology of arbuscular mycorrhiza symbiosis. Annu. Rev. Cell Dev. Biol. **29**:593–617.

Jamil, M., Kountche, B.A., Haider, I., Guo, X., Ntui, V.O., Jia, K.-P., Ali, S., Hameed, U.S., Nakamura, H., Lyu, Y., et al. (2018). Methyl phenlactonoates are efficient strigolactone analogs with simple structure. J. Exp. Bot. **69**:62319–62331.

Jamil, M., Kountche, B.A., Haider, I., Wang, J.Y., Aldossary, F., Zarban, R.A., Jia, K.P., Yonli, D., Shahul Hameed, U.F., Takahashi, I., et al. (2019). Methylation at the C-3'in D-ring of strigolactone analogs reduces biological activity in root parasitic plants and rice. Front. Plant Sci. **10**:353.

Jamil, M., Kountche, B.A., Wang, J.Y., Haider, I., Jia, K.-P., Takahashi, I., Ota, T., Asami, T., and Al-Babili, S. (2020). A new series of carlactonoic acid based strigolactone analogs for fundamental and applied research. Front. Plant Sci. **11**:434.

Jia, K.P., Kountche, B.A., Jamil, M., Guo, X.J., Ntui, V.O., Rufenacht, A., Rochange, S., Al-Babili, S., et al. (2016). Nitro-phenlactone, a carlactone analog with pleiotropic strigolactone activities. Mol. Plant **9**:1341–1344.

Jia, K.P., Baz, L., and Al-Babili, S. (2018). From carotenoids to strigolactones. J. Exp. Bot. **69**:2189–2204.

Jia, K.P., Dickinson, A.J., Mi, J., Cui, G., Kharbatia, N.M., Guo, X., Sugiono, E., Aranda, M., Blilou, I., Rueping, M., et al. (2019). Anchorene is a carotenoid-derived regulatory metabolite required for anchor root formation in *Arabidopsis*. Sci. Adv. **5**:eaaw6787.

Koprna, R., De Diego, N., Dundálková, L., and Spíchal, L. (2016). Use of cytokinins as agrochemicals. Bioorg. Med. Chem. **24**:484–492.

Kountche, B.,A., Jamil, M., Yonli, D., Nikiema, M.P., Blanco-Ania, D., Asami, T., Zwanenburg, B., and Al-Babili, S. (2019). Suicidal germination as a control strategy for *Striga hermonthica* (Benth.) in smallholder farms of sub-Saharan Africa. Plants People Planet. https://doi.org/10.1002/ppp3.32.

Lanfranco, L., Fiorilli, V., Venice, F., and Bonfante, P. (2018). Strigolactones cross the kingdoms: plants, fungi, and bacteria in the arbuscular mycorrhizal symbiosis. J. Exp. Bot. **69**:2175–2188.

Moise, A.R., Al-Babili, S., and Wurtzel, E.T. (2014). Mechanistic aspects of carotenoid biosynthesis. Chem. Rev. **114**:164–193.

Parker, C. (2012). Parasitic weeds: a world challenge. Weed Sci. **60**:269–276.

Pennisi, E. (2010). Armed and dangerous. Science **327**:804–805.

Rigal, A., Ma, Q., and Robert, S. (2014). Unraveling plant hormone signaling through the use of small molecules. Front. Plant Sci. **5**:373.

Samejima, H., Babiker, A.G., Takikawa, H., Sasaki, M., and Sugimoto, Y. (2016). Practicality of the suicidal germination approach for controlling *Striga hermonthica*. Pest Manag. Sci. **72**:2035–2042.

Screpanti, C., Fonné-Pfister, R., Lumbroso, A., Rendine, S., Lachia, M., and De Mesmaeker, A. (2016). Strigolactone derivatives for potential crop enhancement applications. Bioorg. Med. Chem. Lett. **26**:2392–2400.

Takeuchi, J., Jiang, K., Hirabayashi, K., Imamura, Y., Wu, Y., Xu, Y., Miyakawa, T., Nakamura, H., Tanokura, M., and Asami, T. (2018). Rationally designed strigolactone analogs as antagonists of the D14 receptor. Plant Cell Physiol. **59**:1545–1554.

Thind, B.B., and Edwards, J.P. (1986). Laboratory evaluation of the juvenile hormone analogue fenoxycarb against some insecticide-susceptible and resistant stored product beetles. J. Stored Prod. Res. **22**:235–241.

Umehara, M., Hanada, A., Yoshida, S., Akiyama, K., Arite, T., Takeda-Kamiya, N., et al. (2008). Inhibition of shoot branching by new terpenoid plant hormones. Nature **455**:195–200.

Vaidya, A.S., Helander, J.D.M., Peterson, F.C., Elzinga, D., Dejonghe, W., Kaundal, A., Park, S.Y., Xing, Z., Mega, R., Takeuchi, J., et al. (2019). Dynamic control of plant water use using designed ABA receptor agonists. Science **366**:eaaw8848.

Vurro, M., Prandi, C., and Baroccio, F. (2016). Strigolactones: how far is their commercial use for agricultural purposes? Pest Manag. Sci. **72**:2026–2034.

Wakabayashi, T., Hamana, M., Mori, M., Akiyama, R., Ueno, K., Osakabe, K., Osakabe, Y., Suzuki, H., Takikawa, H., Mizutani, M., et al. (2019). Direct conversion of carlactonoic acid to orobanchol by cytochrome P450 CYP722C in strigolactone biosynthesis. Sci. Adv. **5**:eaax9067.

Wang, J.Y., Haider, I., Jamil, M., Fiorilli, V., Saito, Y., Mi, J., Baz, L., Kountche, B.A., Jia, K.P., Guo, X., et al. (2019). The apocarotenoid metabolite zaxinone regulates growth and strigolactone biosynthesis in rice. Nat. Commun. **10**:810.

Waters, M.T., Gutjahr, C., Bennett, T., and Nelson, D.C. (2017). Strigolactone signaling and evolution. Annu. Rev. Plant Biol. **68**:291–322.

Xie, X., and Yoneyama, K. (2010). The strigolactone story. Annu. Rev. Phytopathol. **48**:93–117.

Yoneyama, K., Mori, N., Sato, T., Yoda, A., Xie, X., Okamoto, M., Iwanaga, M., Ohnishi, T., Nishiwaki, H., Asami, T., et al. (2018). Conversion of carlactone to carlactonoic acid is a conserved function of MAX1 homologs in strigolactone biosynthesis. New Phytol. **218**:1522–1533.

Zhang, Y., van Dijk, A.D., Scaffidi, A., Flematti, G.R., Hofmann, M., Charnikhova, T., Verstappen, F., Hepworth, J., van der Krol, S., Leyser, O., et al. (2014). Rice cytochrome P450 MAX1 homologs catalyze distinct steps in strigolactone biosynthesis. Nat. Chem. Biol. **10**:1028–1033.

Molecular Plant, Volume *13*

Supplemental Information

Efficient Mimics for Elucidating Zaxinone Biology and Promoting Agricultural Applications

Jian You Wang, Muhammad Jamil, Pei-Yu Lin, Tsuyoshi Ota, Valentina Fiorilli, Mara Novero, Randa A. Zarban, Boubacar A. Kountche, Ikuo Takahashi, Claudio Martínez, Luisa Lanfranco, Paola Bonfante, Angel R. de Lera, Tadao Asami, and Salim Al-Babili

Efficient Mimics for Elucidating Zaxinone Biology and Promoting Agricultural Applications

Jian You Wang, Muhammad Jamil, Pei-Yu Lin, Tsuyoshi Ota, Valentina Fiorilli, Mara Novero, Randa A. Zarban, Boubacar A. Kountche, Ikuo Takahashi, Claudio Martínez, Luisa Lanfranco, Paola Bonfante, Angel R. de Lera, Tadao Asami and Salim Al-Babili

Supplemental Document 1

Supplemental Methods

Plant material and growth conditions

Rice plants were grown according to Wang et al., 2019 under controlled conditions (a 12 h photoperiod, 200-µmol photons m^{-2} s^{-1} and day/night temperature of 27/25 ^{o}C) with half-strength modified Hoagland nutrient solution, which consisted of 5.6 mM NH_4NO_3, 0.8 mM $MgSO_4 \cdot 7H_2O$, 0.8 mM K_2SO_4, 0.18 mM $FeSO_4 \cdot 7H_2O$, 0.18 mM $Na_2EDTA \cdot 2H_2O$, 1.6 mM $CaCl_2 \cdot 2H_2O$, 0.8 mM KNO_3, 0.023 mM H_3BO_3, 0.0045 mM $MnCl_2 \cdot 4H_2O$, 0.0003 mM $CuSO_4 \cdot 5H_2O$, 0.0015 mM $ZnCl_2$, 0.0001 mM $Na_2MoO_4 \cdot 2H_2O$ and with or without 0.4 mM $K_2HPO_4 \cdot 2H_2O$, resulting in the +Pi and -Pi conditions, respectively with adjusted pH to 5.8.

For treatment of hydroponically grown plants with MiZax, we exposed one week-old seedlings were grown hydroponically containing 2.5 µM compounds (dissolved in 0.1% acetone) for 21 days. The solution was changed every other day, adding the chemical at each renewal.

Rhizotron (48cm x 24 cm x 5cm) experiments were conducted as following: three-day-old seedlings were grown in soil and treated with the compounds at a 5 µM concentration and dissolved in 1 µL/mL emulsifier (Atlas G1086, CRODA, Gouda, The Netherlands; prepared in cyclohexanone according to Kountche et al., 2019) with half-strength modified Hoagland nutrient solution for two weeks. The solution was added twice per week. Root surface area was analyzed with the ImageJ software.

For SL and transcript analysis, one-week-old seedlings were transferred into 50 mL falcon tubes (two seedlings per tube), containing half-strength Hoagland nutrient solution with $K_2HPO_4.2H_2O$ (+Pi), for one week. Rice seedlings were then subjected to phosphate deficiency (-Pi) for another one week. On the day of root exudates collection, rice seedlings were first treated with 5 µM compounds for 6 h, and then root exudates and root tissues were collected for analysis separately.

Synthesis of enantiopure zaxinone and analogues

The synthesis of the (iso)-zaxinone compounds followed the general strategies used for the synthesis of retinoids and carotenoids as described in Supplemental Document S2.

Synthetic methods for zaxinone-mimics (MiZax)

The chemical synthesis procedures and NMR characterization of MiZax are listed in Supplemental Document S3.

Quantitative analysis of SLs in rice root exudates and root tissues

Quantification of SLs in rice root exudates was followed by the protocol described previously by Wang et al., 2019. Briefly, 50 mL of root exudates spiked with 0.672 ng of D_6–5-deoxystrigol, was brought on a 500 mg/3 mL fast SPE C18 column preconditioned with 6 mL of methanol and 6 mL of water. After washing with 6 mL of water, SLs were eluted with 5 mL of acetone. The SLs fraction (acetone-water solution) was concentrated to SL aqueous solution (~1 mL), followed by the extraction with 1 mL of ethyl acetate. 750 µL of 4-deoxyorobanchol enriched organic phase was then transferred to 1.5 mL tube and evaporated to dryness under vacuum. The dried extract was dissolved in 100 µL of acetonitrile: water (25:75, v:v) and filtered through a 0.22 µm filter for LC-MS/MS analysis.

Quantification of SLs in rice root was followed the protocol described previously by Wang et al., 2019. 25 mg of lyophilized plant spiked with 0.672 ng of D_6–5-deoxystrigol were extracted twice with 2 mL of ethyl acetate in an ultrasound bath (Branson 3510 ultrasonic bath) for 15 min, followed by centrifugation for 8 min at 3800 rpm at 4 °C. The two supernatants were combined and dried under vacuum. The residue was dissolved in 100 µL of ethyl acetate and 2 mL of hexane following a Silica gel SPE column (500 mg/3 mL) purification and elution with 3 mL of ethyl acetate and evaporated to dryness under vacuum. The residue was re-dissolved in 200 µL of

acetonitrile: water (25:75, v:v) and filtered through a 0.22 μm filter for LC-MS/MS analysis. The characteristic MRM transitions (precursor ion → product ion) were 331→216, 331→97 for 4-deoxyorobanchol; 347.1→233, 347.1→97 for orobanchol; 337→222, 337→97 for D_6-5-deoxystrigol.

Striga hermonthica seed germination bioassays

Striga hermonthica seed germination bioassays were conducted according to Jamil et al., 2012. Briefly, the root exudates collected from treated and untreated rice, was applied at 50 μL on each six discs containing preconditioned *Striga* seeds. Sterile MilliQ water and standard SL analog GR24 (2.5 μM) were included as a negative and positive control, respectively. After application, *Striga* seeds were incubated in dark at 30 ºC for 24 hours. Germination (seeds with radicle emerging through seed coat) was scored under a binocular microscope, and germination rate (%) was calculated.

Striga hermonthica infection in rice

Striga pot experiment was adapted from Kountche et al., 2019. About 20 mg *Striga* seeds were thoroughly mixed in 1.5 L sand and soil mixture (1:1) and added in 3 L perforated plastic pot containing 0.5 L clean soil in the bottom. The pots were kept in greenhouse at 35 ºC under moisture to precondition *Striga* seeds for 10 days. On the 11th day, five one-week old rice seedlings (cv IAC165) were planted in each pot. After three days of rice planting, each pot was irrigated with 250 mL phosphorus deficient Hoaglands nutrient solution, followed after four hours by irrigation with 25 mL formulated MiZax3, MiZax5 and Zaxinone (100 μM) were sprayed. Next day, each pot was again supplied with 250 mL nutrient solution to adjust the final concentration of each compound to 5 μM and to move the compound to rice seedlings. Compounds were applied twice a week up to four weeks, and after three weeks the number of emerged *Striga* plants in each pot were counted.

Gene expression analysis

Rice roots were ground and homogenized in liquid nitrogen, and total RNA was isolated using a Direct-zol RNA Miniprep Plus Kit following the manufacturer's instructions (ZYMO RESEARCH; USA). cDNA was synthesized from 1 µg of total RNA using iScript cDNA Synthesis Kit (BIO-RAD Laboratories, Inc, 2000 Alfred Nobel Drive, Hercules, CA; USA). The

gene expression level was detected by real-time quantitative RT-PCR (qRT-PCR) which was performed using SYBR Green Master Mix (Applied Biosystems; www.lifetechnologies.com) in a CFX384 Touch™ Real-Time PCR Detection System (BIO-RAD Laboratories, Inc, 2000 Alfred Nobel Drive, Hercules, CA; USA). Primers used for qRT-PCR analysis are listed in Supplemental Table1. The gene expression level was calculated by normalization of a housekeeping gene in rice, Ubiquitin (OsUBQ). The relative gene expression level was calculated according to $2^{-\Delta\Delta}CT$ method (Livak and Schmittgen, 2001).

Chemical stability

MiZax3 and MiZax5 were tested for their chemical stability at 21±1°C with pH 5.0-6.0 aqueous solution following the protocol described by Jamil et al., 2019. 50 µL of each compound solution (1mg $mL^{-}1$) was prepared with 175 µL ethanol and 750 µL Mili-Q water. Thereafter, 25 µL Indanol (1mg $mL^{-}1$, internal standard) was spiked in 975 µL previous prepared solution. The degradation was monitored by UPLC using an Agilent HPLC ZORBAX Eclipse XDB-C_{18} column (3.5 µm, 4.6 × 150 mm), eluted first by 5% acetonitrile in water for 0.5min then by a gradient flow from 5% to 100% acetonitrile within 18 min, and by 100% acetonitrile for 5 min. The column was operated at 40°C at 0.35 mL $min^{-}1$ flow rate. Compounds eluted from the column were detected, and the relative quantity of non-degraded amount was calculated with Indanol.

Detection of MiZax in rice root and shoot tissues

Two-week-old rice plants were treated with the compounds at a 10 µM concentration for 6 h. Plant fresh tissues were then washed and grinded under liquid nitrogen. Separated root and shoot tissues were extracted with 1.5 mL of ethyl acetate in an ultrasound bath (Branson 3510 ultrasonic bath) for 15 min, followed by centrifugation for 8 min at 3800 rpm at 4 °C. The supernatants were transferred to new Eppendorf tubes and dried under vacuum. The residue was re-dissolved in 150 µL of acetonitrile and filtered through a 0.22 µm filter for LC-MS/MS analysis. The analysis of MiZax in rice tissues was performed by using UHPLC- Triple-Stage Quadrupole Mass Spectrometer (Thermo Scientific™ Altis™) with MRM mode. Chromatographic separation was achieved on a ZORBAX Eclipse plus C_{18} column (150 × 2.1 mm; 3.5 µm; Agilent) with mobile phases consisting of water (A) and acetonitrile (B), both containing 0.1% formic acid, and the following linear gradient (flow rate, 0.6 ml/min): 0–6 min, 5%–100 % B, 6–17.5 min, 100 % B,

followed by washing with 100 % B and equilibration with 5 % B. The injection volume was 10 µl, and the column temperature was maintained at 35 °C for each run. The MS parameters were set as follows: positive ion mode, ion source of H-ESI, ion spray voltage of 5000 V, sheath gas of 40 arb, aux gas of 15 arb, sweep gas of 20 arb, ion transfer tube gas temperature of 350 °C, vaporizer temperature of 350 °C, collision energy of 17 eV, CID gas of 2 mTorr. The characteristic MRM transitions (precursor ion → product ion) were 255.1→197.05, 255.1→133.1 for MiZax2; 269.1→211.1, 269.1→133.1 for MiZax3; 289.1→247.2, 289.1→226.9 for MiZax4; 303.1→261.1, 303.1→216.1 for MiZax5

Effect of zaxinone-mimics on *Gigaspora margarita* spores germination

Spores were sterilized in a solution of Streptomycine sulphate (0.03% W/V) and Chloramine T (3% W/V) and germinated in 200 µL of zaxinone, MiZax3, and MiZax5 at 5 µM or 50 nM, GR24 10^{-7} M or upon a mixed solution of MiZax3 (5 µM) + GR24 (10 nM) or MiZax5 (5 µM) + GR24 (10 nM) (12.5 µL acetone in 25 mL water). For each treatments 96 sterilized spores were placed individually in the wells of a multi-well plate and treated with freshly prepared solutions at the beginning of the experiment . Spores were germinated in the dark at 30°C and the germination rate was evaluated after 3 days.

Plant and fungal material and treatments

Seeds of WT plants cv Nipponbare were germinated in pots containing sand and incubated for 10 days in a growth chamber under a 14 h light (23°C)/10 h dark (21°C). Plants used for mycorrhization were inoculated with *Funneliformis mosseae* (BEG 12, MycAgroLab, France). Fungal inoculum (25%) were mixed with sterile quartz sand and used for colonization. Plants were watered with a modified Long-Ashton (LA) solution containing 3.2 µM $Na_2HPO_4 \cdot 12\ H_2O$ (Hewitt, 1966). Ten days after mycorrhizal inoculation, a set of WT plants were treated with 5 µM or 50 nM MiZax3 or MiZax5, by applying molecules twice a week directly in the nutrient solution.

Wild-type and WT treated mycorrhizal roots were stained with 0.1 % cotton blue in lactic acid and the estimation of mycorrhizal parameters was performed by Trouvelot method (Trouvelot et al., 1986). Four parameters were considered: F%, percentage of segments showing internal colonization (frequency of mycorrhization); M%, average percentage of colonization of root segments (intensity of mycorrhization); a%, percentage of arbuscules within infected areas; A%,

percentage of arbuscules in the whole root system. For the molecular analyses, roots were immediately frozen in liquid nitrogen and stored at -80 °C.

Gene expression analysis of mycorrhizal plants

Total genomic DNA was extracted from *F. mosseae* sporocarps and *O. sativa* roots using the DNeasy Plant Mini Kit (Qiagen), according to the manufacturer's instructions. Genomic DNAs were used to test each primers pair designed for real-time PCR to exclude cross hybridization. Total RNA was extracted from rice roots using the Plant RNeasy Kit (Qiagen), according to the manufacturer's instructions. Samples were treated with TURBO™ DNase (Ambion) according to the manufacturer's instructions. The RNA samples were routinely checked for DNA contamination by means of PCR analysis, using primers for OsRubQ1 (Güimil et al. 2005). For single-strand cDNA synthesis about 1000 ng of total RNA was denatured at 65°C for 5 min and then reverse-transcribed at 25°C for 10 min, 42°C for 50 min, and 70°C for 15 min. The reaction was carried out in a final volume of 20 µL containing 10 µM random primers, 0.5 mM dNTPs, 4 µL 5X buffer, 2 µL 0.1 M DTT and 1 µL Super-Script II (Invitrogen). Quantitative RT-PCR (qRT-PCR) was performed using a Rotor-Gene Q 5plex HRM Platform (Qiagen). Each PCR reaction was carried out in a total volume of 15 µL containing 2 µL diluted cDNA (about 10 ng), 7.5 µL 2X SYBR Green Reaction Mix, and 2.75 µL of each primer (3 µM). The following PCR program was used: 95°C for 90 sec, 40 cycles of 95°C for 15 sec, 60°C for 30 sec. A melting curve (80 steps with a heating rate of 0.5°C per 10 sec and a continuous fluorescence measurement) was recorded at the end of each run to exclude the generation of non-specific PCR products. All reactions were performed on at least three biological and three technical replicates. Baseline range and take off values were automatically calculated using Rotor-Gene Q 5plex software. Transcript level of *OsPT11* (Güimil et al. 2005); *OsLysM* (Fiorilli et al., 2015) and *Fm18S* (Balestrini et al., 2007) were normalized using OsRubQ1 housekeeping gene (Güimil et al. 2005). Primers used for qRT-PCR analysis are listed in Supplemental Table1. Only take off values leading to a Ct mean with a standard deviation below of 0.5 were considered. Statistical tests were carried out through one-way analysis of variance (one-way ANOVA) and Tukey's post hoc test, using a probability level of $p<0.05$. All statistical elaborations were performed using PAST statistical (version 2.16; Hammer et al. 2001). To analyze the fungal intraradical morphology root apparata were stained in

Cotton Blue (0,1% W/V) in lactic acid, cut in pieces 1cm long and observed under an optical microscope.

Supplemental Figures

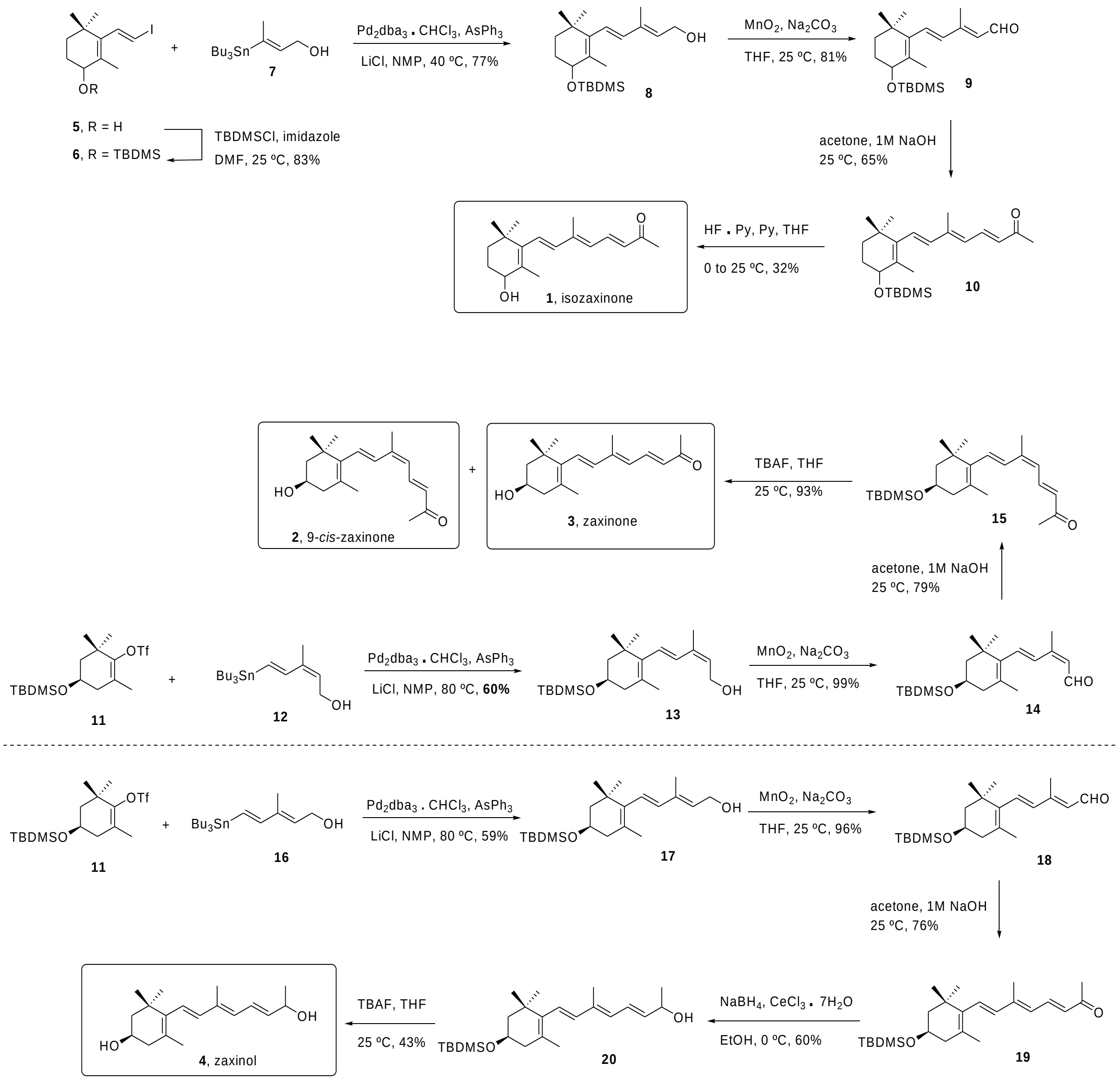

Supplemental Figure 1. Chemical Synthesis of Zaxinone.

The synthesis of the zaxinone and its analogs followed the general strategies used for the synthesis of retinoids and carotenoids as described in Supplemental Document S2.

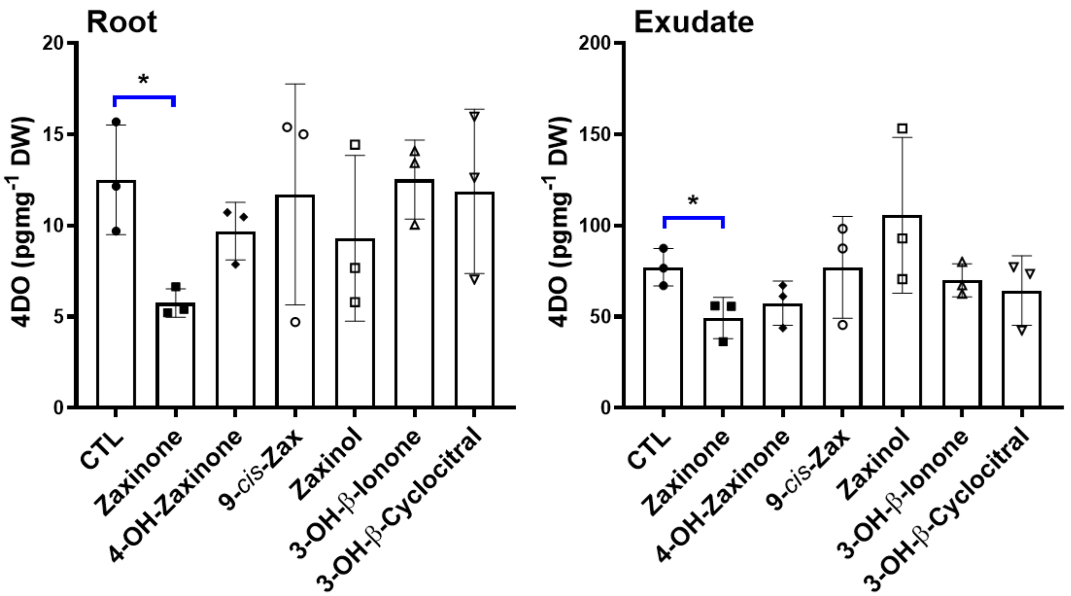

Supplemental Figure 2. Effect of Apocarotenoids on SL Content in Root Tissues and Exudates.

SL, 4-deoxyorobanchol (4-DO), quantification in wild-type roots and root exudates in response to zaxinone, 4-OH-zaxinone, 9-*cis*-Zaxinone, zaxinol, 3-OH-β-Ionone, and 3-OH-β-Cyclocitral at 5 µM under Pi starvation. Bars represent mean ± SD; *n*=3 biological replicates; Statistical analysis was performed using a two-tailed *t*-test. *, $P < 0.05$.

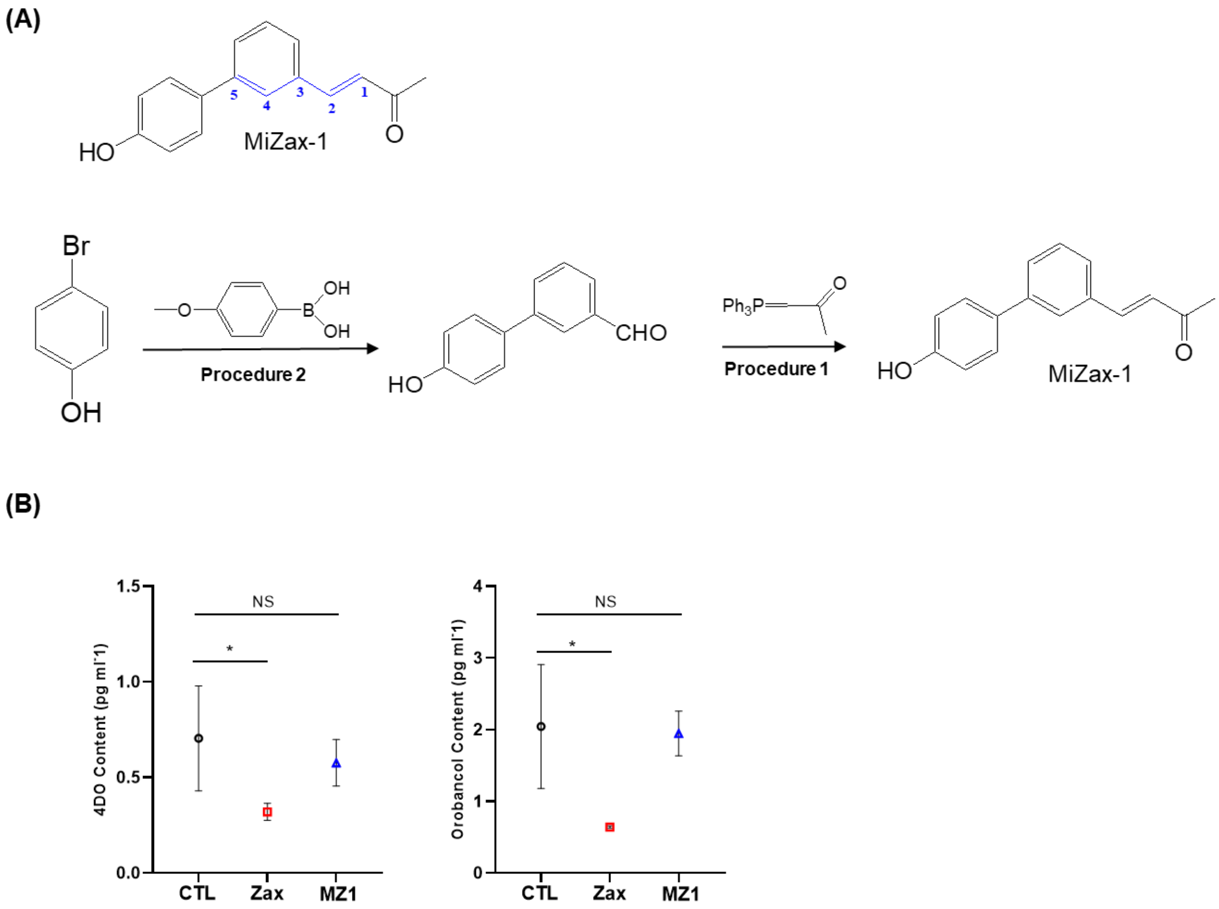

Supplemental Figure 3. Effect of MiZax1 on Rice SL Release.

(A) Chemical structure of MiZax1 and the corresponding synthesis scheme. Numbers in blue indicate the distance between phenyl ring and the ketone group.

(B) Quantification of SLs, 4-deoxyorobanchol (4-DO) and Orobanchol (Oro) , in wild-type root exudates in response to zaxinone, and MiZax1 at 5 µM under Pi starvation. Bars represent mean ± SD; *n*=3 biological replicates; statistical analysis was performed using a two-tailed *t*-test. *, P < 0.05. NS, non-significant; CTL, Control; Zax, Zaxinone; MZ1, MiZax1

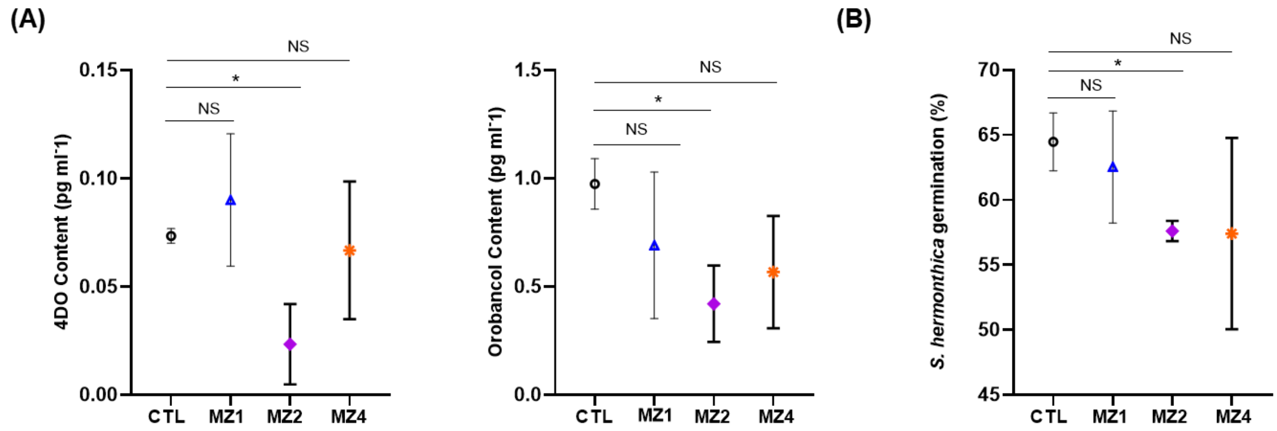

Supplementary Figure 4. Comparison of MiZax on rice SL release.

(A) Quantification of SLs, 4-deoxyorobanchol (4-DO) and Orobanchol (Oro) , in wild-type root exudates in response to MiZax1, MiZax2, or MiZax4 at 5 µM under Pi starvation.

(B) *Striga* seed germination activity of rice root exudates isolated from plants treated with MiZax1, MiZax2, or MiZax4 at 5 µM under Pi starvation.

Bars represent mean ± SD; *n*=3 biological replicates; statistical analysis was performed using *t*-test. *, $P < 0.05$. NS, non-significant;CTL, Control; Zax, Zaxinone; MZ1, MiZax1; MZ2, MiZax2; MZ4, MiZax4

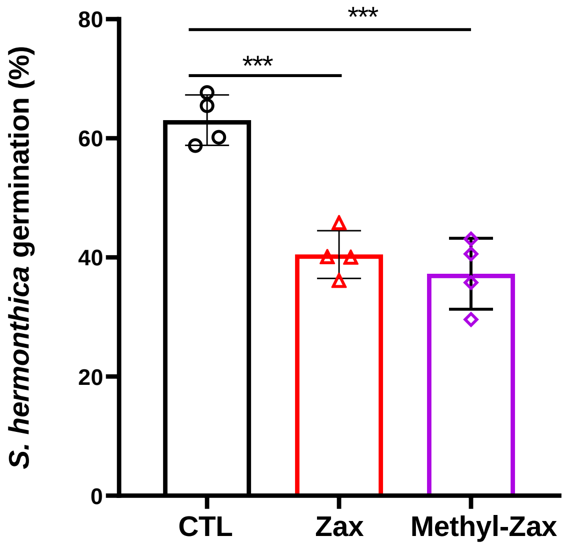

Supplementary Figure 5. Bioactivity of Methyl-Zax in rice.

Striga seed germination activity of rice root exudates isolated from plants treated with Zaxinone, Methyl-Zaxinone at 5 µM under Pi starvation. Bars represent mean ± SD; *n*=4 biological replicates; statistical analysis was performed using *t*-test. ***, P < 0.001. CTL, Control; Zax, Zaxinone; Methyl-Zax, Methyl-Zaxinone.

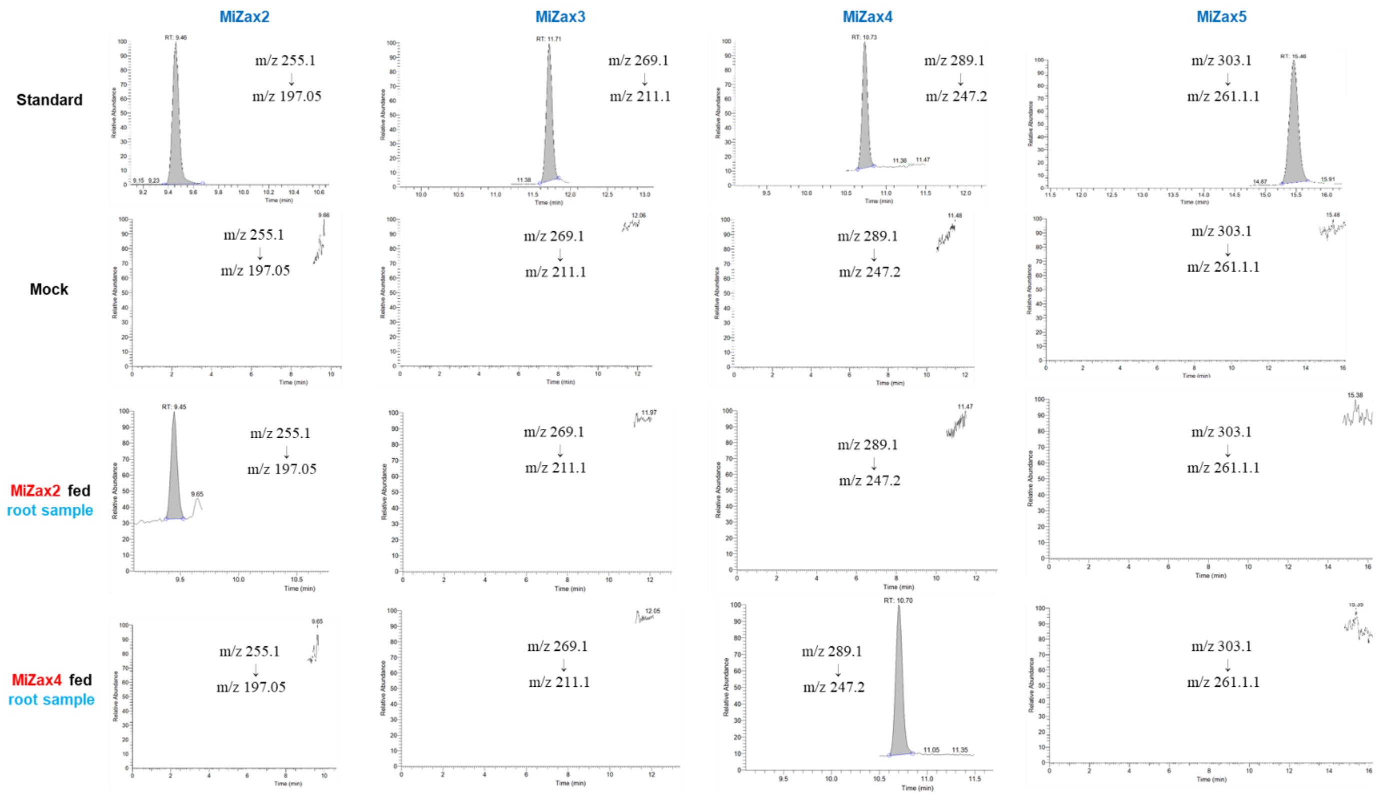

Supplementary Figure 6. LC-MS detection of MiZax2/4 in root tissues.

Roots of rice plants fed with MiZax2 or MiZax4 accumulated both mimics but did not convert them into MiZax3 or MiZax5, respectively.

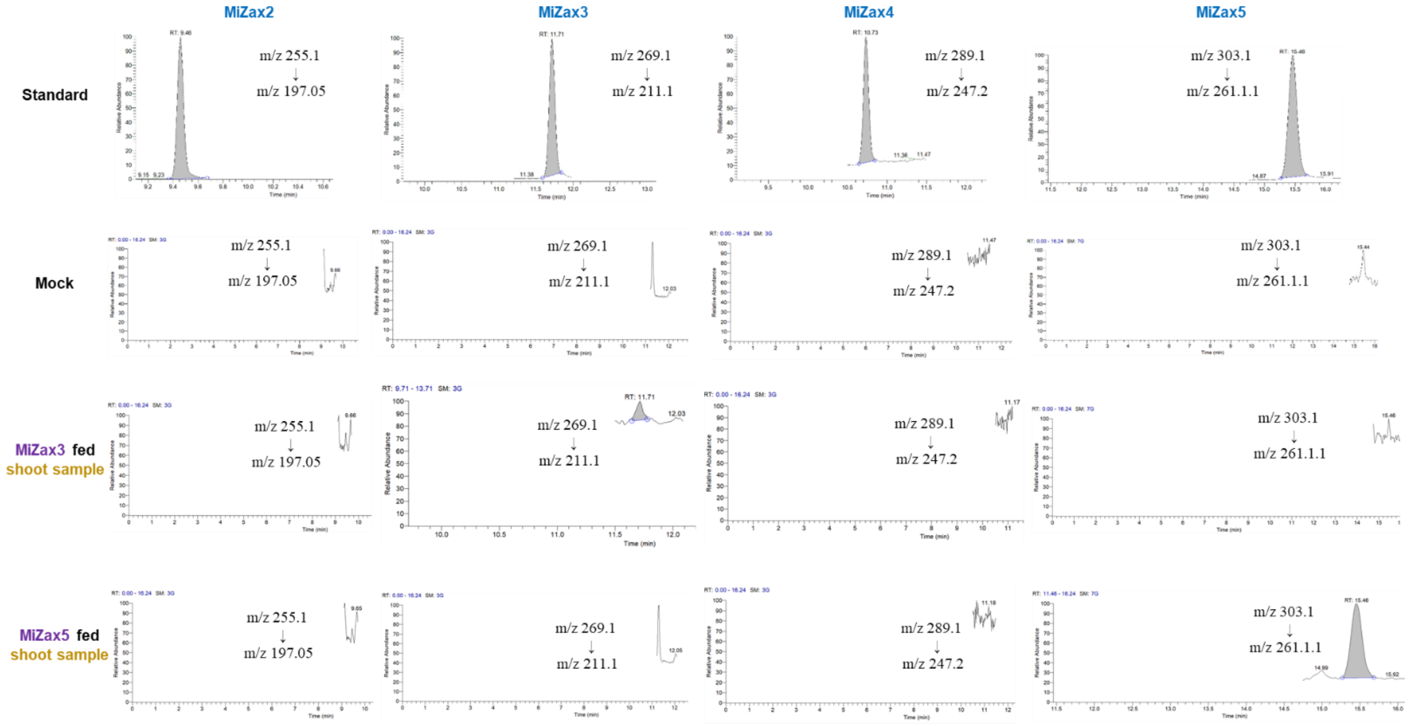

Supplementary Figure 7. LC-MS detection of MiZax3/5 in shoot tissues.

MiZax3 or MiZax5 fed through rice roots is transported to their shoots.

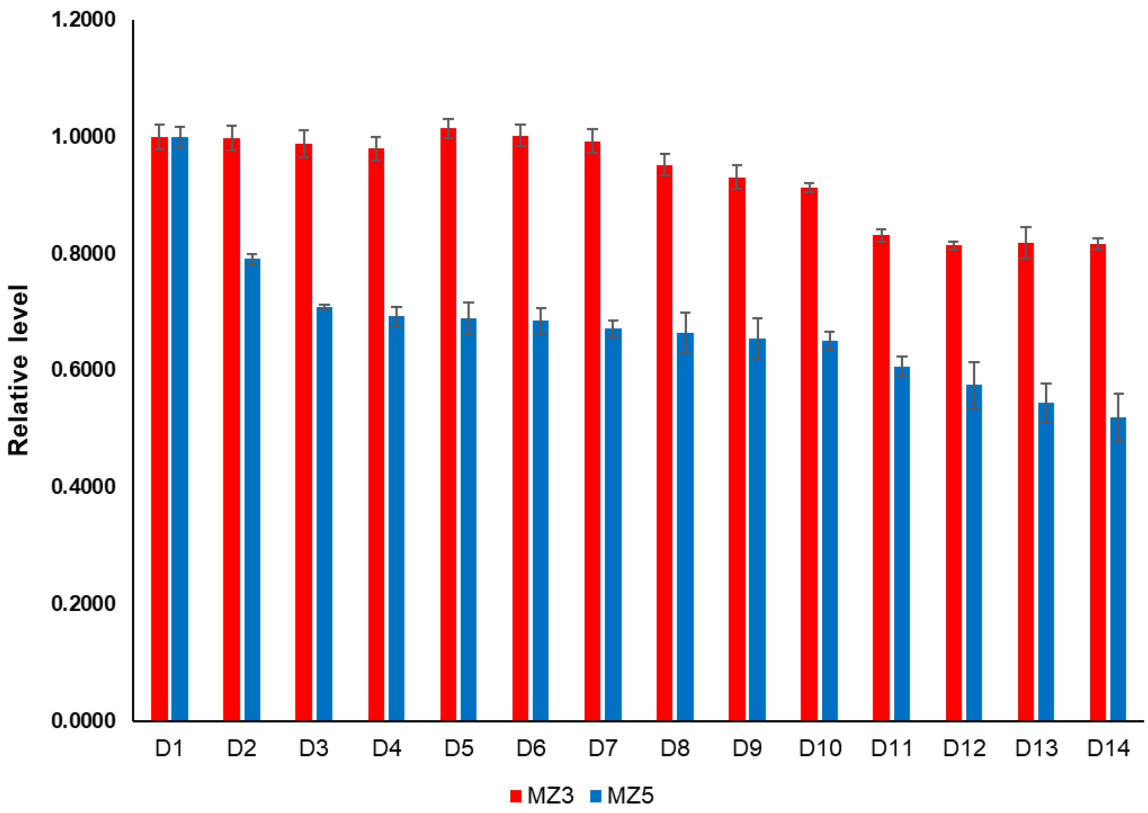

Supplemental Figure 8. Stability of MiZax by HPLC Analysis.

The relative amount of non-degraded analogs was monitored in HPLC for 14 days. Data are means ± SE (n = 3). X-axis: time (days); Y-axis: relative levels. MZ3, MiZax3; MZ5, MiZax5.

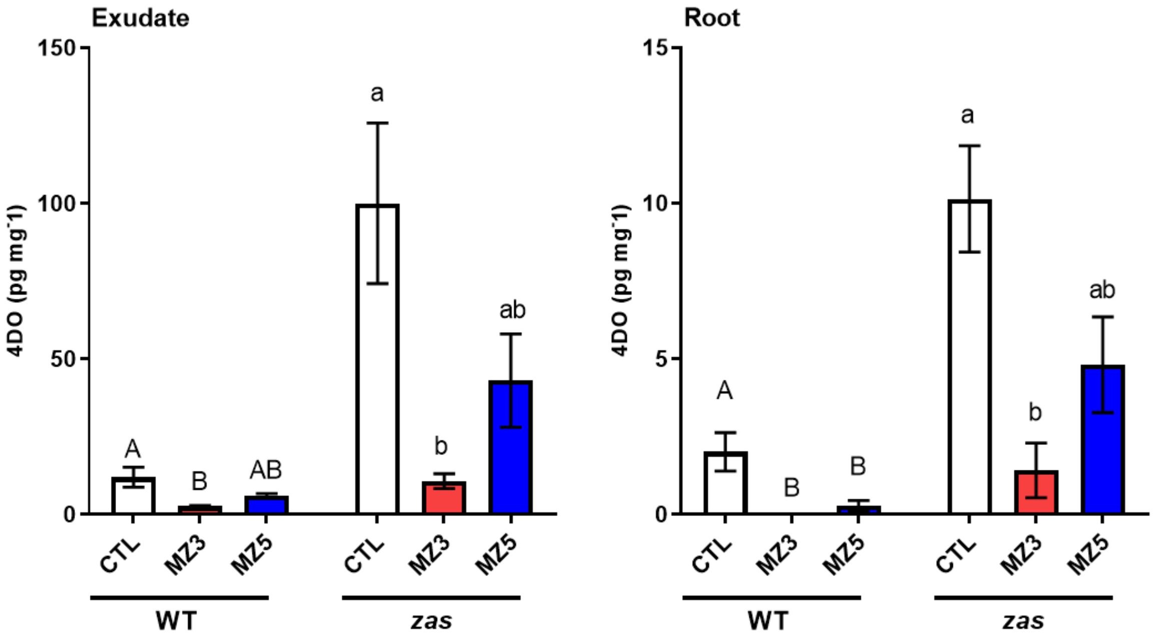

Supplemental Figure 9. Effect of MiZax on SL Content in Root Tissues and Exudates.

SL, 4-deoxyorobanchol (4-DO), quantification in wild-type and *zas* mutant root exudates (left) and roots (right) in response to MZ3 and MZ5 at 5 µM under Pi starvation. Bars represent mean ± SD; *n*=4 biological replicates; statistical analysis was performed using one-way analysis of variance (ANOVA) and Tukey's *post hoc* test. Different letters denote significant differences ($P < 0.05$). CTL, Control; MZ3, MiZax3; MZ5, MiZax5.

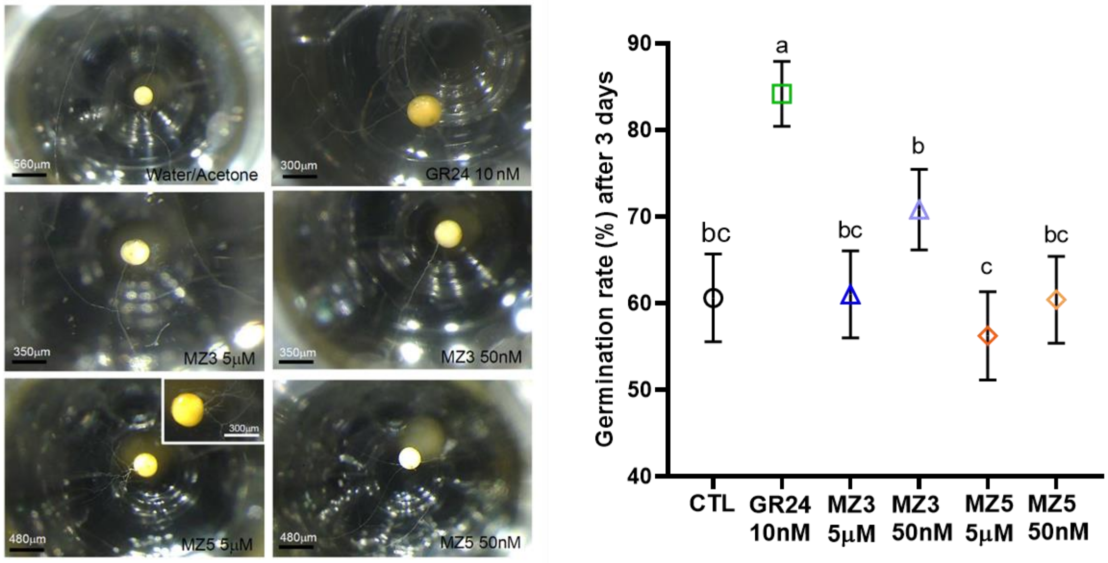

Supplemental Figure 10. Effect of MiZax on *Gigaspora margarita* Spores Germination.

Bars represent mean ± SE; n=96 biological replicates; Data indicated with different letters are statistically different according to the non-parametric Kruskal-Wallis test. Different letters denote significant differences ($P < 0.05$). CTL, Control (acetone); MZ3, MiZax3; MZ5, MiZax5; GR24, a synthetic SL analog.

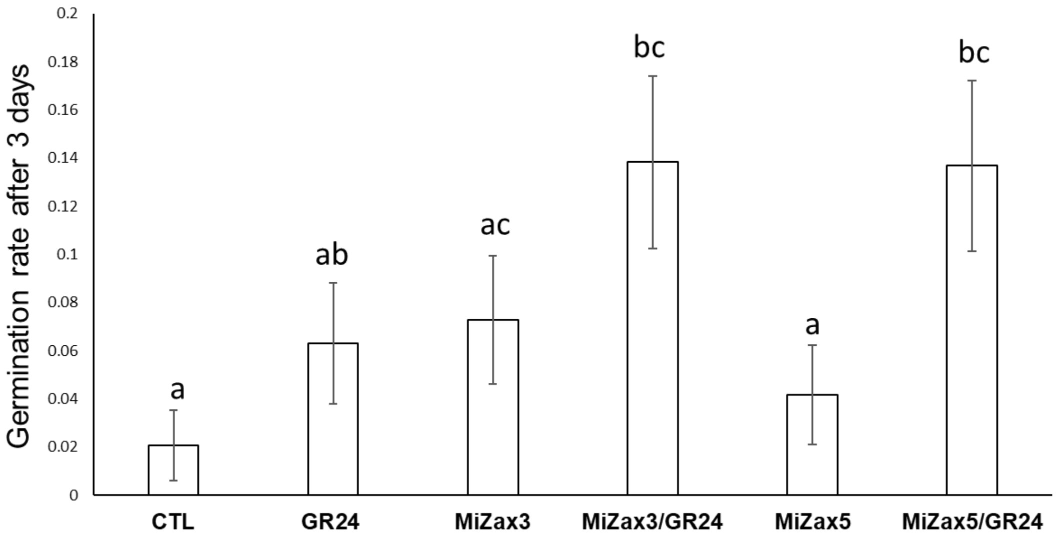

Supplemental Figure 11. Effect of MiZax on SL-mediated *Gigaspora margarita* spore germination. Bars represent mean ± SE; *n*=96 biological replicates; Data indicated with different letters are statistically different according to the non-parametric Kruskal-Wallis test ($p<0.05$). CLT: spores treated with acetone; GR24 (SL analog): spores treated with GR24 (10 nM); MiZax3: spores treated with MiZax3 (5 µM); MiZax3/GR24: spores treated with MiZax3 (5 µM) and GR24 (10 nM); MiZax5: spores treated with MiZax5 (5 µM); MiZax5/GR24: spores treated with MiZax5 (5 µM) and GR24 (10 nM).

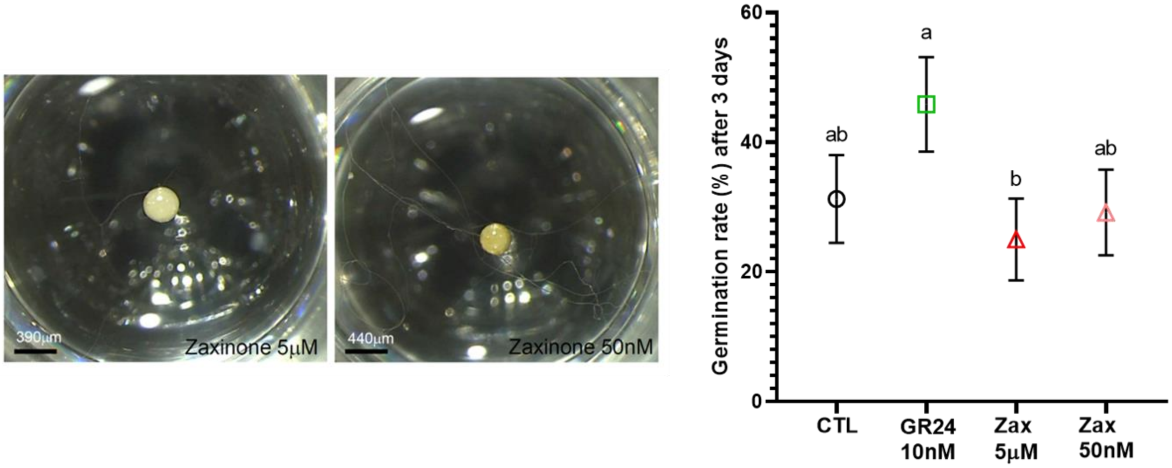

Supplemental Figure 12. Effect of Zaxinone on *Gigaspora margarita* Spores Germination. Bars represent mean ± SE; *n*=96 biological replicates; Data indicated with different letters are statistically different according to the non-parametric Kruskal-Wallis test ($p<0.05$). CTL, Control (acetone); Zax, Zaxinone; GR24, a synthetic SL analog.

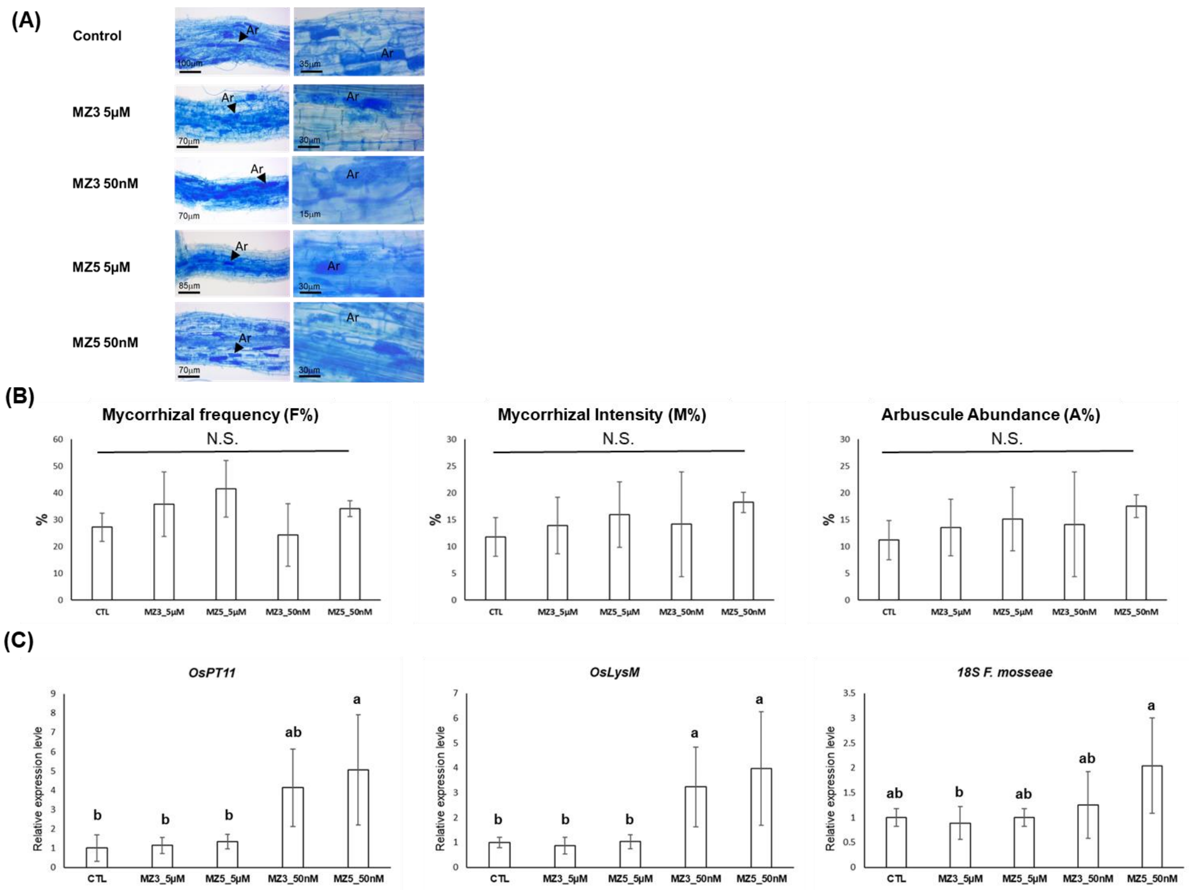

Supplemental Figure 13. Evaluation of MiZax Effects on the AM Symbiosis.

(A) Details of arbuscule morphology in roots stained with cotton blue. CTL, Control; MZ3, MiZax; MZ5, MiZax5. ; Ar, Arbuscule- containing cells.

(B) Mycorrhizal colonization in non-treated and treated plants by the AM fungi *Funneliformis mosseae* at 35 dpi. Degree of colonization expressed as mycorrhizal frequency (F %), intensity (M %) and arbuscule abundance (A %) in the root system of WT plants non treated and treated with 5µM and 50nM Mizax3 or Mizax5. Data are the average of four biological replicates ± SE. CTL, Control; MZ3, MiZax; MZ5, MiZax5; N.S., non-significant.

(C) Molecular evaluation of mycorrhization by qRT-PCR analysis of mRNA abundance of plant AM-responsive genes (*OsPT11*, *OsLysM*) and fungal housekeeping gene (*18S F. mosseae*) on mycorrhizal roots considering the whole root system. Bars represent mean ± SD; n=4 biological replicates. Statistical analysis was performed using one-way analysis of variance (ANOVA) and Tukey's *post hoc* test.

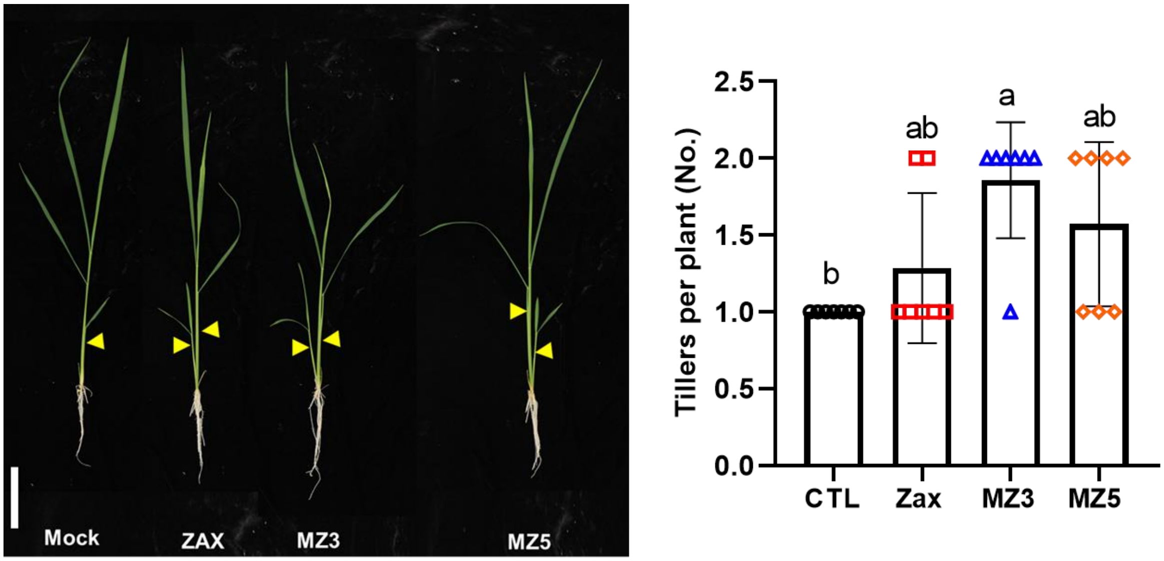

Supplemental Figure 14. Effect of Zaxinone, MZ3, and MZ5 at 2.5 µM on tillers of IAC165 seedlings grown hydroponically.

Tillers are indicated by yellow arrows points. Each data point represents one plant, *n*=7. Data represent mean ± SD. Statistical analysis was performed using one-way analysis of variance (ANOVA) and Tukey's *post hoc* test. Different letters denote significant differences ($P < 0.05$). Scale bars=8 cm. CTL, Control; Zax, Zaxinone; MZ3, MiZax3; MZ5, MiZax5.

Supplemental Table 1. Primer sequences used in this study

Experiment	Primer name	Sequence (5′–3′)
qRT-PCR	OsUbi-Q F	GCCCAAGAAGAAGATCAAGAAC
	OsUbi-Q R	AGATAACAACGGAAGCATAAAAG
	OsD27 F	CTTCCAAGCTACATCCTCAC
	OsD27 R	CCCAACCAACCAAGGAAA
	OsCCD7 F	CAGTCTCCAAGCACAGATG
	OsCCD7 R	GTTCTTTGGCACCTCTAGTT
	OsCCD8b F	TGGCGATATCGATGGTGA
	OsCCD8b R	GACCTCCTCGAACGTCTT
	OsMax1-900 F	ATTGTCAGCGATCCACTTC
	OsMax1-900 R	GCGCCGTTCTTGAAATTG
	OsRubQ1 F	GGGTTCACAAGTCTGCCTATTTG
	OsRubQ1 R	ACGGGACACGACCAAGGA
	OSPt11 F	GAGAAGTTCCCTGCTTCAAGCA
	OSPt11 R	CATATCCCAGATGAGCGTATCATG
	OsLysM F	CGCTGACATGCAACAAGGTG
	OsLysM R	CTTCGCGCAGTTGATGTTTGG
	18SF.m F	CCTTTTGAGCTCGGTCTCGTG
	18SF.m R	TGGTCCGTGTTTCAAGACG

Supplemental References

Balestrini, R., Go´mez-Ariza, J., Lanfranco, L., Bonfante, P. (2007). Laser microdissection reveals that transcripts for five plant and one fungal phosphate transporter genes are contemporaneously present in arbusculated cells. Mol. Plant Microbe. In. 20: 1055-1062.

Fiorilli, V., Vallino, M., Biselli, C., Faccio, A., Bagnaresi, P., and Bonfante, P. (2015).Host and non-host roots in rice: cellular and molecular approaches reveal differential responses to arbuscular mycorrhizal fungi. Front. Plant Sci. **6**:636.

Güimil, S., Chang, H.S., Zhu, T., Sesma, A., Osbourn, A., Roux, C., Ioannidis, V., Oakeley, E.J., Docquier, M., Descombes, P. et al. (2005). Comparative transcriptomics of rice reveals an ancient pattern of response to microbial colonization. Proc. Natl Acad. Sci. USA **102**: 8066-8070.

Hammer, Ø., Harper, D.A.T., and Ryan, P.D. (2001).PAST: paleontological statistics software package for education and data analysis. Palaeontol.Electron. **4**:9.

Hewitt, E.J. (1966). Sand and water culture methods used in the study of plant nutrition. Farnham Royal, UK: Commonwealth Agricultural Bureaux.

Trouvelot, A., Kough, J. L. and Gianinazzi-Pearson, V. in Mycorrhizae. Physiology and Genetics (eds Gianinazzi-Pearson, V. & Gianinazzi, S.) 217–221 (INRA Press, Paris, 1986)

Jamil, M., Kanampiu, F. K., Karaya, H., Charnikhova, T., and Bouwmeester, H. J. (2012). Striga hermonthica parasitism in maize in response to N and P fertilisers. Field Crops Res. **134**:1-10

Jamil, M., Kountche, B.A., Haider, I., Wang, J.Y., Aldossary, F., Zarban, R.A., et al (2019). Methylation at the C-3'in D-ring of strigolactone analogs reduces biological activity in root parasitic plants and rice. Front. Plant Sci. **10**: 353.

Kountche, B, A., Jamil, M., Yonli, D., Nikiema, M.P., Blanco-Ania, D., Asami, T., Zwanenburg, B., Al-Babili, S. (2019). Suicidal germination as a control strategy for *Striga* hermonthica (Benth.) in smallholder farms of sub-Saharan Africa. Plants, People, Planet doi.org/10.1002/ppp3.32

Livak, K. J. and Schmittgen, T. D. (2001). Analysis of relative gene expression data using real-time quantitative PCR and the $2^{\Delta\Delta}$CT Method. Methods **25(4)**:402-408.

Wang, J.Y., Haider, I., Jamil, M., Fiorilli, V., Saito, Y. et al. (2019). The apocarotenoid metabolite zaxinone regulates growth and strigolactone biosynthesis in rice. Nat. Commun. **10**:810. https://doi.org/10.1038/s41467-019-08461-1
